# Microbial Diversity and Antimicrobial Resistance Profile in Microbiota From Soils of Conventional and Organic Farming Systems

**DOI:** 10.3389/fmicb.2019.00892

**Published:** 2019-04-26

**Authors:** Julija Armalytė, Jūratė Skerniškytė, Elena Bakienė, Renatas Krasauskas, Rita Šiugždinienė, Violeta Kareivienė, Sigita Kerzienė, Irena Klimienė, Edita Sužiedėlienė, Modestas Ružauskas

**Affiliations:** ^1^Life Sciences Center, Institute of Biosciences, Vilnius University, Vilnius, Lithuania; ^2^Institute of Microbiology and Virology, Lithuanian University of Health Sciences, Kaunas, Lithuania

**Keywords:** organic and conventional farming, soil microbiota, antibiotic susceptibility, resistance genes, efflux pumps

## Abstract

Soil is one of the biggest reservoirs of microbial diversity, yet the processes that define the community dynamics are not fully understood. Apart from soil management being vital for agricultural purposes, it is also considered a favorable environment for the evolution and development of antimicrobial resistance, which is due to its high complexity and ongoing competition between the microorganisms. Different approaches to agricultural production might have specific outcomes for soil microbial community composition and antibiotic resistance phenotype. Therefore in this study we aimed to compare the soil microbiota and its resistome in conventional and organic farming systems that are continually influenced by the different treatment (inorganic fertilizers and pesticides vs. organic manure and no chemical pest management). The comparison of the soil microbial communities revealed no major differences among the main phyla of bacteria between the two farming styles with similar soil structure and pH. Only small differences between the lower taxa could be observed indicating that the soil community is stable, with minor shifts in composition being able to handle the different styles of treatment and fertilization. It is still unclear what level of intensity can change microbial composition but current conventional farming in Central Europe demonstrates acceptable level of intensity for soil bacterial communities. When the resistome of the soils was assessed by screening the total soil DNA for clinically relevant and soil-derived antibiotic resistance genes, a low variety of resistance determinants was detected (resistance to β-lactams, aminoglycosides, tetracycline, erythromycin, and rifampicin) with no clear preference for the soil farming type. The same soil samples were also used to isolate antibiotic resistant cultivable bacteria, which were predominated by highly resistant isolates of *Pseudomonas, Stenotrophomonas, Sphingobacterium* and *Chryseobacterium* genera. The resistance of these isolates was largely dependent on the efflux mechanisms, the soil *Pseudomonas* spp. relying mostly on RND, while *Stenotrophomonas* spp. and *Chryseobacterium* spp. on RND and ABC transporters.

## Introduction

Microbiota of the soil is greatly important for life on our planet, including its role in the cycling of carbon, nitrogen and other nutrients (Jansson and Hofmockel, 2018). Bacteria and other soil microorganisms are the agents of biotransformation of soil organic matter and nutrients and of most key soil processes. Their activities are influenced by both soil physico-chemical processes and ecological interactions (Powlson et al., 2001). As a habitat for microorganisms, soil is a very diverse and complex substrate on the planet. Conventional approaches based on isolation of the cultivable microbes and techniques based on the analysis of the total DNA in the soil show an enormous diversity in the microorganism composition (Torsvik et al., 1990). Culture-based methods suggest that a gram of soil contains for about one hundred species of microorganisms (Dunbar et al., 1999), but such data are underestimated because multiple lines of evidence indicate that fewer than 1% of the species in soil are presently cultivable (Amann et al., 1995). DNA based methods revealed that soils typically contain 10^9^ to 10^10^ microorganisms per gram, which may represent thousands of bacterial species (Gans et al., 2005). Therefore, metagenomic and other next-generation sequencing based studies might be very useful for the studying the soil microbiome for understanding soil microbial functioning (Baveye, 2009; Raynaud and Nunan, 2014; Mandal et al., 2015).

Soil serves a range of different functions and it is the basis for forestry and agriculture and the importance of this role to be expected to increase (Fischer and Heilig, 1997). Although it is important to keep the soil microbiome stable, agricultural intensification carries dangers including the possibility of damaging soil functions. Latest studies have shown that anthropogenic activities, such as intensification of agriculture and land use change, reduce bacterial numbers and the overall diversity of soil microorganisms. During the past years studies had largely focused on the effects of specific microbial groups, such as fungi, soil bacteria and soil fauna. However, interactions of soil organisms are very complex and therefore changes in diversity within one trophic group or functional guild may alter the diversity, prevalence and functioning of another (Wagg et al., 2014).

Antimicrobial resistance is one of the biggest problems in human and animal medicine at present. Since a high percentage of antibiotics are discharged from the human or animal body without degradation, this means that different habitats, from the human body to river water or soils, are polluted with antibiotics (Martínez, 2017). Antibiotics from treatment of farm animals can accumulate in the farm sludge, which is afterward spread as a fertilizer on the farmland (Larsson, 2014), however, there is limited knowledge of antimicrobial concentrations that might exert selection for resistant bacteria in the environment (Bengtsson-Palme and Larsson, 2016). The concentrations of antibiotics in soils usually are low in most ecosystems, but even low concentrations may trigger specific bacterial responses, and analysis of such responses is a topic of interest (Martínez, 2017). Even though the usage of antibiotics is considered one of the most important risk for the development of antimicrobial resistance, the emergence of the resistance in clinical environment can also be based on the theory about a pre-existing pool of antibiotic resistance genes in natural environmental reservoirs and a transferability of these genes (Nesme and Simonet, 2015).

The aims of this study were twofold: (1) to investigate and compare microbiomes in soils of organic and conventional farming systems and (2) to analyze antimicrobial resistance profiles in soil microbiota.

## Materials and Methods

### Soil Selection and Sampling

The soil samples were collected from six farming fields in Lithuania (located at the borderline of the zones Dfb and Cfb according to the Köppen climatic zones (Peel et al., 2007) during the year 2016. The six collection points of the soil represented two different types of farming, organic and conventional (intensive), and three agrocultures grown in the field during the year of collection (winter wheat, rapeseed, maize). The organic farming sites were known not to use inorganic fertilizers or pesticides for the time period of over 20 years and were fertilized only with organic fertilizers (farmyard manure and slurry). The conventional farming fields were fertilized with inorganic NPK fertilizers (3–4 times a year) and the cultures were regularly sprayed with herbicides, insecticides and fungicides. The pairs (organic and conventional) of farming soil samples were collected from two winter wheat fields, located 1.8 km apart (coordinates: 54.925416, 24.464575 and 54.933504, 24.488816) in October 2016; two rapeseed fields, located 17 km apart (54.921779, 24.463984 and 54.807963, 24.640339) and two maize fields, located 2.3 km apart (55.423267, 24.166897 and 55.41869, 24.202844) in December 2016. The type of the soil in the winter wheat and rapeseed fields was sandy loam whereas in the maize fields – sandy clay loam. In each field, samples were collected from 10 places all over the plot area from the depth of 20 cm using tubular soil sampler. Samples then were placed into sterile plastic bags and delivered to the laboratory during the time of 2 h, where the material was pooled and mixed. The samples were kept at +2°C until the next day for the cultivation of bacteria or aliquoted and frozen at -80°C for the DNA extraction.

### DNA Extraction

For microbial community analysis total DNA was extracted using Quick-DNA Fecal/Soil Microbe kit (Zymo Research, United States) according to the manufacturer’s instructions. For resistance gene detection by PCR total soil DNA was extracted by FastDNA^TM^ SPIN Kit for Soil (MP Biomedicals, United States), which was then additionally purified as described elsewhere (Young et al., 1993). DNA material for identification of species of cultivable soil bacteria and determination of antimicrobial resistance genes was obtained after bacterial lysis according to the extraction protocol prepared by the EU Community Reference Laboratory for Antimicrobial Resistance with modifications as described previously (Ruzauskas et al., 2014).

### Soil Microbial Community and Data Analysis

Metagenomic sequencing of 16S rRNA and microbial profiling analysis was performed as described previously (Merkeviciene et al., 2017). Alpha diversity indexes were calculated with EstimateS (v. 8.2). The prevalence of separate taxonomic units of bacteria in soils of organic and conventional farming was given as the percentage from the total number of DNA reads. The differences among the prevalence of bacteria of the most abundant taxonomic units in organic and conventional soils were compared using Fisher’s Exact Test for Count Data. Comparison of the taxonomic distribution of resistant isolates from organic and conventional farming was assessed using Fisher’s Exact Test for Count Data. Statistical analysis was performed using IBM SPSS Statistics 20 package. Results were considered statistically significant if *p* < 0.05.

### Selection of Resistant Isolates

For the isolation of antibiotic resistant bacteria the soil samples were suspended in water (1:2) and inoculated onto solid media Tryptone Soy Agar (Thermo Scientific, United Kingdom) supplemented with the following antimicrobial agents: ciprofloxacin, gentamicin, imipenem, trimethoprim, ceftazidime, and chloramphenicol. Only a single antibiotic was used per plate. As there are no clinical breakpoints set for most of the soil bacteria, the concentrations of antimicrobials in media were used as clinical breakpoints set by EUCAST for *Pseudomonas, Acinetobacter*, and *Enterobacteriaceae* for isolation and selection of Gram-negative bacteria as well as for *Enterococcus* in case of Gram-positive microbiota. The concentrations of antibiotics in media for resistance screening were as follows: ciprofloxacin – 2 μg/mL for gram-negatives and 8 μg/mL for gram-positives; gentamicin – 8 μg/mL; imipenem – 16 μg/mL; trimethoprim – 8 μg/mL; ceftazidime – 16 μg/mL and chloramphenicol, which breakpoint was taken from CLSI standard – 32 μg/mL. Plates were incubated for 72 h at + 22°C. After incubation, separate predominant colonies were selected for further purification to obtain pure cultures of different bacterial species from each soil sample.

### Antibiotic Susceptibility Testing

Antimicrobial susceptibility testing was performed on selected isolates by broth micro-dilution method suing Sensititre^®^plates and the ARIS 2X automated system (Thermo Scientific, United States). Interpretation of results was carried-out using manufacturers software (SWIN^®^). The minimum inhibitory concentrations (MIC) of tested antibiotics are presented in Supplementary Table S1.

### Identification of the Isolated Soil Bacteria

Identification of bacteria isolates was based on 16S rRNA fragment sequencing. For this purpose PCR using universal primers 27F and 515R (Supplementary Table S2) was performed as described previously (Kim et al., 2012) using DNA extracted from bacteria isolates. PCR products then were purified using DNA Clean and Concentrator-5 Kit (D4010, Zymo Research, United States) and identification of the isolates was performed after sequencing and analysis using Molecular Evolutionary Genetic Analysis software (MEGA, version 6). Basic local alignment search tool (BLAST) was used for comparison of obtained sequences with sequences in the database of National Center for Biotechnology Information (NCBI, United States). Species were identified by matching obtained sequences with a sequence showing the highest maximum identity score from the GenBank database. If the identity of the best match was < 99% and query cover < 96% only genus was assigned.

### Antibiotic Resistance Gene Detection

The presence of genes encoding antibiotic resistance determinants was assessed by PCR at the same conditions as described earlier (Seputiene et al., 2012). Two sets of genes were screened in this study: the first set included clinically relevant ARGs, that have been previously shown to be important in the antibiotic resistance of pathogenic bacteria (the genes tested and specific primers used are described in Supplementary Table S2).

The other set comprised ARGs, naturally occurring in soil bacteria and chosen for analysis (Supplementary Table S2) based on their reported occurrence in metagenomes of soil samples obtained from different geographical locations (Allen et al., 2009; Torres-Cortés et al., 2011; McGarvey et al., 2012; Wichmann et al., 2014) and on the abundance in different species (presence in minimum three different species, non-identical hits) according to the BLAST (NCBI BLASTN, Bacteria domain, Nucleotide collection (nr/nt)) search. Of 149 ARGs analyzed bioinformatically, 10 mostly widespread genes were selected for further analysis (Figure 2). Primers for amplification of their DNA were designed by the alignment of homologous sequences of different species using Clustal Omega and identification of the conservative regions. To expand the sensitivity of detection, degenerative primers were designed (Supplementary Table S2).

A PCR amplifying 16S rDNA fragment (primers Frrs/Rrrs) was used in parallel as amplification control.

### The Efflux Pump Activity Detection

To elucidate the contribution of multidrug resistance efflux pumps to bacteria antibiotic resistance, synergistic assays with antibiotics and specific efflux pump inhibitors were performed. First, the MICs of antibiotics and inhibitors was accessed by Broth microdilution method (Wiegand et al., 2008) for each isolate tested. Then MIC of the antibiotic was evaluated with 1/2 of inhibitor MIC present in the mix. Microtiter plates were incubated at 28°C for 19 h.

## Results

### Composition of Bacterial Community in Organic and Conventional Farming Soil

The organic and conventional winter wheat fields, located 1.8 km apart, were chosen for the analysis. Both soils had neutral pH (7.08 and 6.58 for organic and conventional farming), humus content of 2.8 and 1.5%, and amounts of phosphorus (P_2_O_5_) of 320 mg/kg and 130 mg/kg in organic conventional farming soils, respectively. Total DNA was extracted from both soils and used for 16S rRNA gene sequencing in order to analyze the microbial community composition. In total 93,212 and 192,939 sequences were obtained, with Good’s coverage indexes of 0.995 and 0.998, indicating that sufficient number of reads was obtained to evaluate the bacterial diversity for the both respective soils. Alpha diversity of the samples was: Shannon index 5.87 and 6.07, and Chao1 2364.04 and 2735.3 for organic and conventional wheat field soil, respectively.

**FIGURE 1:**
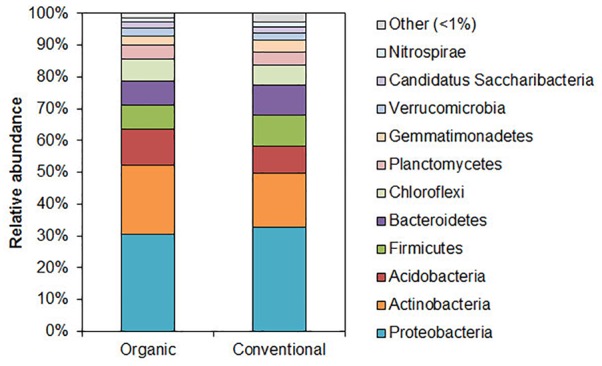
Relative abundance of bacterial phyla in organic and conventional wheat farming soils. Bacterial community composition determined using 16S rRNA sequencing-based analysis. Only the phyla that were present in relative abundance of > 1% are indicated.

The 97 and 98 % of sequences were identified as DNA belonging to kingdom *Bacteria* in both samples, respectively. The relative abundance of the main bacterial phyla (comprising > 1% of reads) is presented in Figure 1 (all the species detected are presented in Supplementary Data Sheets S1, S2). The predominant phylum in the soil samples from of organic and conventional wheat field was *Proteobacteria* (30–33%), followed by *Actinobacteria* (22–17%), *Acidobacteria* (11–9%), *Firmicutes* (8–10%) and *Bacteroidetes* (7–10%), respectively. No obvious differences could be detected among the main phyla.

Distributions of the most prevalent genera (with prevalence above 0.5 % from the total bacteria) in the soils of organic and conventional farming sites are presented in Supplementary Table S3. Although *Acidobacterium* and *Bacillus* statistically significantly were the most predominant genera (*p* < 0.001), their prevalence in general was under 5 % from a total population of microbiota in both soils. As could be seen from the Supplementary Table S3, the same genera were most prevalent in both soils and had only limited amount of difference in organic and conventional soils. The highest statistically reliable differences were among *Bacillus, Gemmatimonas* which prevalence was higher in the conventional soil as well as between *Holophaga, Acidobacteriaceae, Hyphomicrobium, Flavobacterium* and *Nocardioides* which were more abundant in the organic soil (*p* < 0.05).

As an increase in the relative abundance of phylum *Actinobacteria* could be observed in the organic wheat soil, we therefore checked which of the lower taxa were contributing most to the change. The more abundant (over 1% relative abundance) orders of *Actinobacteria, Rubrobacterales* (with the most abundant family *Gaiellaceae*), *Acidimicrobiales* (family *Acidimicrobiaceae*) and *Solirubrobacterales* (family *Conexibacteraceae*) constituted 5.83% in organic farming soil, which was two-fold higher than in conventional soil. The more abundant genera (Supplementary Table S3) in the organic farming soil that were overrepresented comparing to conventional farming soil were also mostly of phylum *Actinobacteria* (genera *Gaiella, Ilumatobacter, Iamia*), but also *Holophaga* of phylum *Acidobacteria* was also abundant. In conventional farming soil an increase in the abundance of order *Sphingobacteriales* (with the most abundant family *Sphingobacteriaceae*) was observed. Several genera were also more abundant, *Rhodanobacter* was only detected in conventional soil, while genera *Rhizobium, Agrobacterium, Devosia* (phylum *Alphaproteobacteria*) and genus *Paenibacillus* (phylum *Firmicutes*) were more abundant in the conventional farming soil.

### Detection of Antibiotic Resistance Genes (ARGs) in the Soil DNA

The differences in the microbial community composition of the two farming type soils were observed only between the smaller taxa. The overall composition was comparable between the tested soils, as well as similar to the composition of various soils around the world (Fierer et al., 2009). However, we were interested if the prevalence of ARGs in the soils of different farming systems differed. Genes, commonly found in the clinically important bacteria and conferring resistance to the different classes of antibiotics used in the human and veterinary medicine, were included in the study. In addition, ARGs, naturally found in the soil bacteria and conferring resistance to β-lactams, aminoglycosides, tetracycline and rifampicin were screened.

**FIGURE 2:**
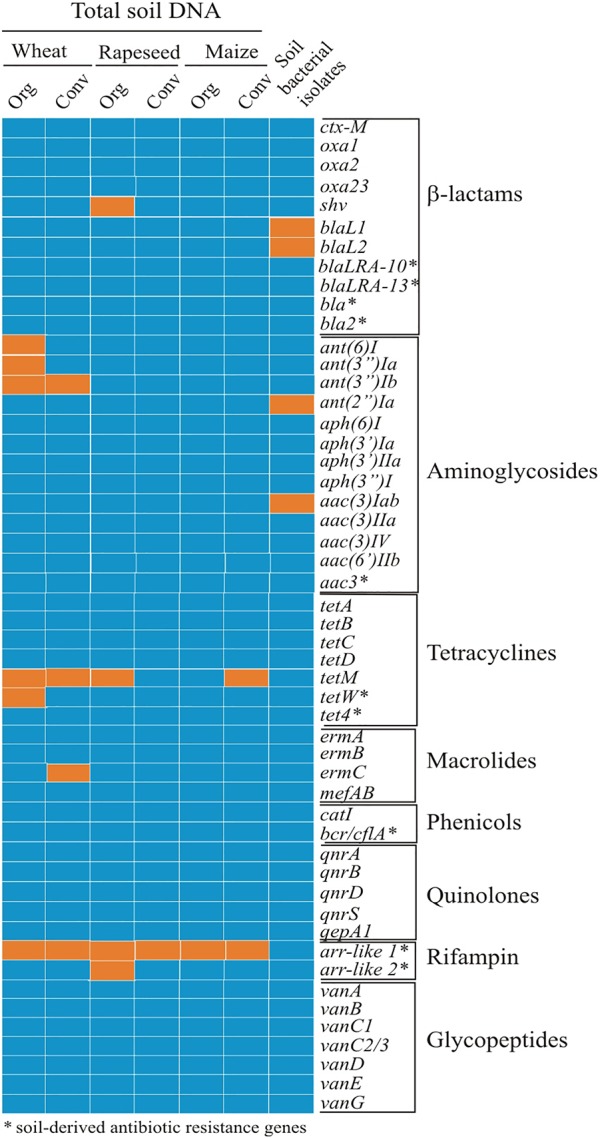
The resistance genes in the total DNA and in bacterial isolates obtained from organic and conventional farming soils. The names of genes tested are listed on the right in groups regarding their mediated resistance to specific antibiotic class. Total DNA from soils of organic (Org) and conventional (Conv) farming sites and cultivable bacterial isolates (shown in a single column) were screened. Orange panel denotes gene present; blue panel – gene not detected. Resistance genes identified in soil bacteria were found as follows: blaL1–in a single S. *maltophilia* isolate from the soil of conventional rapeseed farming; blaL2–in four S. *maltophilia* isolates from the same field; ant(2″)Ia – in four *Pseudomonas* spp. (one organic wheat and organic rapeseed soil and two from conventional maize soil) and one Sphingobacter sp. isolate (conventional rapeseed soil); aac(3)Iab – in five *Pseudomonas* spp. from organic maize field.

The total DNA was purified from the six soils of organic and conventional farming type, as described in “Materials and Methods.” Winter wheat soils, described previously, were used and in addition organic and conventional pairs of rapeseed and maize soils were selected. The measured pH of the soils was 7.16 and 7.95 for rapeseed, and 8.15 and 7.81 for respective farming types of maize. The purified DNA was used for PCR with the gene-specific primers listed in the Supplementary Table S2. Primers targeting soil bacteria-specific resistance genes were designed as described in “Materials and Methods.” The gene screen identified the extended spectrum β-lactamase (ESBL) coding gene *shv* in the organic farming rapeseed field soil (Figure 2). No other clinically relevant β-lactamase coding genes were observed. From the genes of known clinical relevance, only those coding for aminoglycoside modifying enzymes were found. The *ant*(*6*)*I, ant*(*3*″)*Ia* and *ant*(*3*″)*Ib*, genes, coding for streptomycin modifying nucleotidyltransferases and conferring streptomycin resistance (Vakulenko et al., 2003) were detected in the organic farming wheat field soil. The *ant*(*3*″)*Ib* gene was also found in a soil DNA from conventional farming field, together with the *ermC* gene coding for rRNA methylase conferring erythromycin resistance. Tetracycline resistance gene *tetM* encoding ribosome protection protein (Burdett et al., 1982) was more common and found in the soil of four fields out of six tested (Figure 2).

In the next series of the soil resistance gene screen, we targeted the genes, which were previously detected by screening the metagenomic libraries constructed using DNA from a broad range of geographic locations and several types of environmental sources (soil and manure). The 10 selected genes (Figure 2) coded for the proteins of five families, including aminoglycoside acetyltransferases, β-lactamases, rifampin ADP-ribosyltransferases, transporters of tetracyclines and chloramphenicol. Aminoglycoside 3-N-acetyltransferase coding gene *aac3* (resistance to gentamicin), β-lactamase gene *bla* (resistance to ampicillin) and *bcr/cfl* gene coding efflux pump (resistance to chloramphenicol), were obtained from metagenomics libraries from agricultural soils from Spain (Torres-Cortés et al., 2011). Two *arr*-like genes (named here *arr*-like 1 and *arr*-like 2) coding for rifampin ADP-ribosyltransferase variants(rifampin resistance) showing highest similarity to the homologs from *Oscillatoria* sp. isolate and *tet4*gene (tetracycline resistance), coding for ABC transporter with the highest similarity to a homolog from *Paenibacillus curdlanolyticus* were identified in metagenomic libraries of soil from urban environment in Seattle, United States (McGarvey et al., 2012). Screening of the metagenomic libraries from a dairy cow manure (United States) (Wichmann et al., 2014) revealed *bla2* gene (resistance to carbenicillin) showing high sequence identity to a β-lactamase previously found only in *Firmicutes*. Ribosome modifying *tetW* gene demonstrated resistance to tetracycline and had homologs in both *Firmicutes* and *Actinobacteria*. And finally, functional metagenominc library from DNA extracted from the remote Alaskan soil (Allen et al., 2009) discovered *bla_LRA-10_* and *bla_LRA-13_* genes, which demonstrated highest homology to a class C β-lactamases from *Mycobacterium smegmatis* and *Shewanella baltica*, respectively.

Our PCR screening of this gene set in DNA from all soils identified *arr*-like gene variant 1, coding for rifampin-modifying ADP-ribosyltransferase and conferring resistance to rifampicin. Other above listed genes were not detected with the exception of another *arr-*like gene variant 2 and *tetW* gene in single soil (Figure 2).

### The Abundance of Antibiotic Resistant Species in the Soils

**FIGURE 3:**
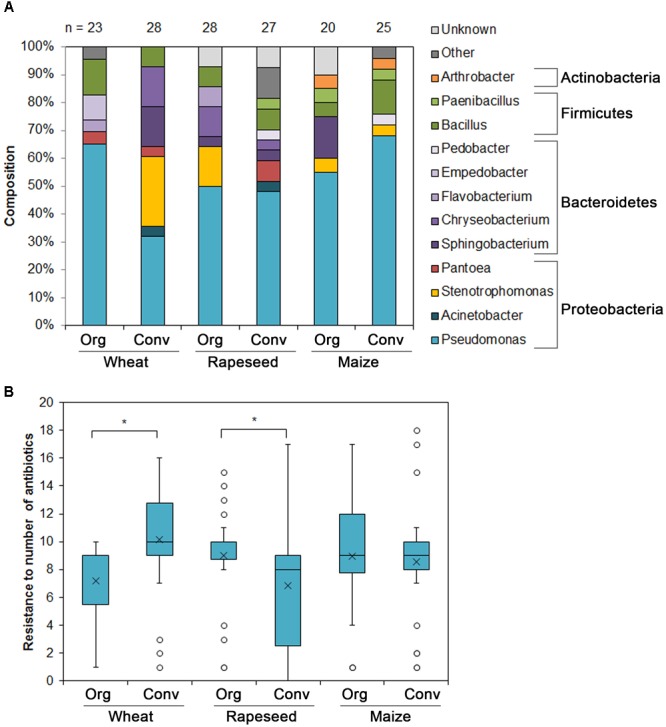
Cultivable antibiotic resistant soil bacteria isolated from six soils under different farming styles. **(A)** The abundance of resistant bacterial genera isolated from soils under different farming styles. **(B)** The abundance of antibiotic resistant bacteria isolated from various soils. Bacteria were designated resistant if the MIC values met with EUCAST clinical breakpoints. Boxes indicate upper and lower quartiles, whiskers indicate minimum and maximum values excluding outliers, circles depict outliers and crosses indicate mean values. ^∗^Indicates statistical significance calculated as non-parametric Mann-Whitney test for two independent samples (*p* < 0.05; one-tailed).

To further access the prevalence of the antibiotic resistance in bacteria from soils of organic and conventional farming, we have isolated cultivable resistant bacteria as described in section “Materials and Methods.” In total 151 isolates were recovered from the six soils. The majority of the isolates in all the soils belonged to the genus *Pseudomonas* (*n* = 79). Other more abundant genera included *Stenotrophomonas* (*n* = 13), *Bacillus* (*n* = 13), *Sphingobacterium* (*n* = 9) and *Cryseobacterium* (*n* = 8) (Figure 3A).

The MIC values were calculated as described in section “Materials and Methods.” The isolate was designated as resistant if MIC value matched EUCAST clinical breakpoints (v. 7.0, 2017) for the bacteria belonging to *Pseudomonas, Acinetobacter* genera and *Enterobacteriaceae*. If the breakpoints were not available, the PK/PD (non-species related) breakpoints were assigned. The majority of the strains showed resistance to more than one antibiotic tested or even to several antibiotic classes. We calculated the average number of antibiotics, to which isolates recovered from the each soil, were resistant (Figure 3B). The bacteria from the conventional farming wheat field soil were more antibiotic resistant compared with those recovered from the organic farming site and the difference was significant. On the contrary, the bacteria isolated from the rapeseed field soil of organic farming were more antibiotic resistant compared with those recovered from the soil of conventional farming site. The differences between the soils where maize was cultivated were not significant.

### Detection of Clinically Relevant ARGs in Cultivable Bacteria

The resistant isolates were screened by PCR for the presence of clinically relevant ARGs. The results in Figure 2 show that only genes responsible for aminoglycoside resistance were found. Interestingly, *aac*(*3*)*Iab* gene, coding for the member of N-acethyltransferase superfamily, was found in five *Pseudomonas* sp. isolates, all derived from ecological maize field soil. Different MIC profiles indicated they are not the same strain. The other aminoglycoside resistance gene *ant*(*2”*)*Ia*, coding for aminoglycoside O-nucleotidyltransferase, commonly encoded in transposons and plasmids (Vakulenko and Mobashery, 2003), was found in four *Pseudomonas* sp. and one *Sphingobacterium* isolate from soils of various origins (Figure 2). The aminoglycoside resistance genes observed in isolated bacteria differed from the ones found in total soil DNA. We also checked for species specific *Stenotrophomonas maltophilia* gene *bla_L1_* coding for metallo-β-lactamase and the gene *bla_L2,_* coding for serine-β-lactamase (Flores-Treviño et al., 2014) in isolates identified as the latter species (*n* = 6) (Supplementary Table S1). The *bla_L2_* gene was present in four *S. maltophilia* isolates, all recovered from intensive wheat farming soil; one of the four also had *bla_L1_* gene. None of cultivable bacterial isolates contained naturally occurring antibiotic resistance-related genes (Figure 2).

### Resistance Due to Efflux Pumps

Our observation, that most abundant groups of soil bacterial isolates, exhibiting a high antibiotic resistance, carried rather a limited number of genes coding for modifying enzyme-based resistance mechanisms, prompted as to test the impact of efflux pumps (EPs) on the resistance displayed by these isolate groups. Most research has been focused upon *P. aeruginosa* and resistance nodulation-cell division (RND) superfamily exporters, which play the major role in the drug expulsion (Li X.Z. et al., 2015). As the majority of cultivable antibiotic resistant isolates from the soil in this study were of the genus *Pseudomonas*, we firstly investigated the impact of RND EPs.

Twenty four *Pseudomonas* spp. isolates from wheat farming soils were examined for the resistance to chloramphenicol, which is known as a substrate of RND EP (Li X.Z. et al., 2015). To access the influence of EPs we have used specific inhibitors and examined their impact on the antibiotic MIC value as described in “Materials and Methods.” The phenylalanine-arginine-β-naphthylamide (PAβN) it is most active and best studied inhibitor of RND EPs (Rampioni et al., 2017).

**FIGURE 4:**
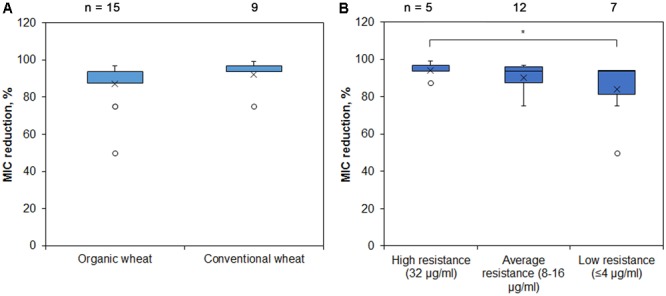
MIC reduction of *Pseudomonas* spp. of different soil origin after addition of RND EP inhibitor PAβN. *Pseudomonas* spp. isolates were grown with or without RND EP inhibitor PAβN and their MIC of chloramphenicol was assessed. Blue boxes indicate upper and lower quartiles, whiskers indicate minimum and maximum values excluding outliers, circles depict outliers and crosses indicate mean values. ^∗^ Indicates statistical significance calculated as non-parametric Mann-Whitney test for two independent samples (*p* < 0.05; one-tailed). **(A)** *Pseudomonas* spp. isolates from two farming sites of different style did not show significant differences in MIC reduction after addition of RND EP inhibitor. **(B)** *Pseudomonas* spp. with the higher initial resistance to chloramphenicol were more dependent on EP than the isolates with low initial resistance.

The initial chloramphenicol MIC varied between 0.5 and 32 μg/ml, and the difference of MIC values between the *Pseudomonas* spp. isolates of different soil origin was not statistically significant (data not shown). However, all the isolates tested showed drastic reduction of resistance to chloramphenicol after addition of PAβN, the average MIC reduction being 89 % (the least reduction of MIC was 50%, while the highest -99%), indicating the major role of RND EPs (Figure 4A). We then checked how the initial resistance is related to the RND activity and observed that isolates with high initial chloramphenicol MIC were more RND-EPs-dependent compared with those with low initial resistance level and this difference was significant (Figure 4B).

Investigation of the impact of EPs on *Pseudomonas* spp. resistance to ampicillin, again, showed a considerable reduction of antibiotic MIC levels in the presence of PAβN in all bacterial isolates, clearly demonstrating an important role of RND pumps. However, as the resistance of the isolates to ampicillin was often very high (unmeasurable under the protocol used), therefore it was impossible to calculate MIC reduction accurately (data not shown).

Next, we accessed the role of other prominent efflux system, ABC transporters, in the bacterial susceptibility to chloramphenicol by using an inhibitor of ABC EPs verapamil (Li et al., 2016). The decrease of chloramphenicol MIC after addition of verapamil was low to absent (data not shown), indicating that ABC efflux transporters are not the main cause of antibiotic resistance in *Pseudomonas* spp. recovered from soil. However, a substantial synergistic effect of combined action of PAβN and verapamil on antibiotic MIC was observed, suggesting that operation of low-efficient ABC pumps may be masked in the background of active RND pumps (data not shown).

**FIGURE 5:**
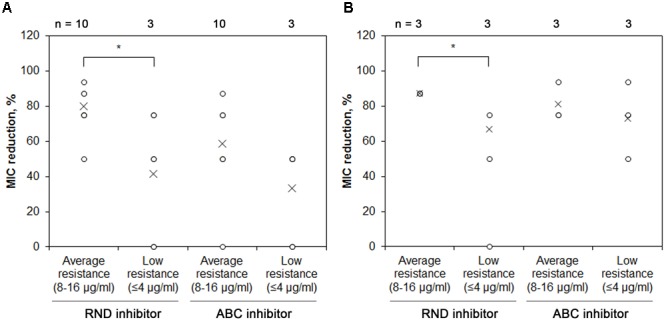
MIC reduction of *Stenotrophomonas* spp. and *Chryseobacterium* spp. after addition of RND or ABC efflux pump inhibitors. **(A)** *Stenotrophomonas spp*. or **(B)** *Chryseobacterium* spp. isolates were grown with or without RND EP inhibitor PAβNor ABC inhibitor verapamil and their MIC of chloramphenicol was assessed. Each value is indicated as a circle, crosses indicate mean values.^∗^ Indicates statistical significance calculated as non-parametric Mann-Whitney test for two independent samples (*p* < 0.05; two-tailed).

Other clinically relevant bacteria of the soil origin (*Stenotrophomonas* spp. and *Chryseobacterium* spp.) which showed resistance to a high number of antimicrobials (Supplementary Table S1) were checked for the activity of RND and ABC types of EPs by using pump-specific inhibitors. *Stenotrophomonas* spp. were affected by inhibition of RND pumps (average reduction being 62%), especially when initial chloramphenicol MIC values for isolates were high (Figure 5A). However, some isolates exhibited MIC reduction comparable to the *Pseudomonas* spp. (up to 94 %), while one did not show any chloramphenicol MIC changes after EP inhibitor addition. Similar tendency of greater importance of RND efflux pumps could be observed for the more initially resistant isolates. Inhibition of ABC EP also substantially affected the resistance to chloramphenicol (average MIC reduction being 59 %, and maximum reduction of 87%) (Figure 5A). Two strains did not show a change in chloramphenicol MIC after addition of verapamil, one of them was the same strain that exhibited the trait with PAβN. Similar effect was also observed for *Chryseobacterium* spp. (Figure 5B). Therefore, we show that antimicrobial resistance in the most prevalent cultivable soil bacteria is largely mediated by the efflux pumps.

## Discussion

Soil is a very complex structure which includes organic particles as well as thousands of living organisms from different taxa including worms, arthropods, fungi, bacteria and some other eukaryotic and prokaryotic organisms. Bacteria are one of the most important living parts of the soil ecosystem (Fierer, 2017; Sun et al., 2017). Many of them are decomposers, the other helps to assimilated nitrogen for plants as well as they serve as a food for protists. Recent study demonstrates that high abundances of beneficial bacteria are related with soil quality, which is indicated by better plant growth, lower outbreaks of diseases, higher soil pH and better nutrient activities (Wang et al., 2017). The findings also suggest that soil pH is the primary determinant and it is more important factor than addition of nutrients for bacterial community (Wu et al., 2017; Zhang et al., 2017). We have investigated near-neutral soils (pH 6.58–7.08) and found a wide variety but similar microbial composition in soils of different farming types. The relative abundance of most bacterial phyla is higher in near-neutral than in acidic or alkaline soils (Zhang et al., 2017). *Proteobacteria, Actinobacteria, Acidobacteria, Firmicutes* and *Bacteroidetes* were the most abundant phyla in our study. The recent data demonstrates that those bacteria are more prevalent in near-neutral pH except *Acidobacteria* which are diverse and specific acidobacterial subgroups are adapted to distinct pH conditions (Lauber et al., 2009; Bartram et al., 2014; Zhang et al., 2017). The chemical soil composition, particularly the amount of phosphorus is also important factor for microbial load (Liu et al., 2013) but it is unclear the relation between amount of phosphorus and microbial variety. In our experiments we did not detect any significant changes in microbial composition at the genera level when different amount of phosphorus (130 mg/kg vs. 320 mg/kg) was presented in a soil of different farming. Within the most prevalent genera the highest difference was among the prevalence of the genus *Holophaga* which number was almost two times higher in the soil of organic crops. *Holophaga* are homoacetogenic bacteria that degrades methoxylated aromatic compounds which are natural products of plants, animals and microorganisms (Liesack et al., 1994), however, more investigations are necessary to determine the reason of such difference. The stability of soil microbiome composition is very important for N and S cycles but certain pesticides and other chemicals may affect the composition of bacteria therefore, making serious ecological disturbances in living ecosystems (García-Delgado et al., 2018; Karas et al., 2018). At the same time there are some data that application of different herbicides including glyphosate, glufosinate, paraquat, paraquat-diquat and triasulfuron had no effect on the diversity and structure of soil bacteria and archaea (Pose-Juan et al., 2017; Dennis et al., 2018).

In this study we aimed to analyze the soils from two farming systems: conventional and organic (which were certified as organic farming for at least 20 years). Both conventional and to a lesser extent organic farming depend on pesticides, though the systems are subjected to different regulations. Organic farming exclusively allows the use of pesticides which are of natural origin, whereas synthetically produced products may be applied in conventional farming systems (Lori et al., 2017). Analysis of the bacterial diversity in soils from different farming systems showed only slight differences among the main taxonomical units of microorganisms. The main prevalent phyla included *Proteobacteria, Actinobacteria, Acidobacteria, Firmicutes* and *Bacteroidetes* in the soils from both farming systems. Both soils had a similar composition to the soil detected all around the globe (Fierer et al., 2009).

Only rarely detected lower taxons were different between the soils. From the genera that were present in significantly different quantities (higher in conventional farming), *Sphingomonas* and *Gemmatimonas* were observed previously to be increased in farming with mineral fertilizers (Ma et al., 2018). We also found *Rhodanobacter* genus, which was previously connected with denitrification of soil (Green et al., 2012), present only in conventional farming soil (0.37% relative abundance) and absent from organic farming soil.

*Acidobacteria* are related to nutrient-wise poor soil (Chaudhry et al., 2012), and therefore their abundance would be an indicator of poor quality of soil. The relative abundance of *Acidobacteria* was not high in both soils we have investigated, indicating both farming systems are able to retain soil quality. A relative abundance of *Firmicutes* has been previously connected with manure application to the soil (Hartmann et al., 2015; Wepking et al., 2017). Yet in our analysis we have also found higher relative abundance of *Firmicutes* in the conventional farming soil.

We also observed that the continuous pesticide use on the field did not affect the soil community composition, confirming a similar observation made previously (Hartmann et al., 2015). Increased diversity and richness of the microbial community has been previously observed in the organic farming, which is mostly due to the fertilization using manure, while continuous fertilization using mineral fertilizers decreases the diversity (Li et al., 2012; Hartmann et al., 2015; Lupatini et al., 2016). In our case, we did not observe significant differences between the two types of farming soils.

High variety and similarities of microorganisms in the soils from different farming systems indicates the stability of microbial populations that might be associated with the evolutionary ability of soil microorganisms to adapt the different environment and to survive among other organisms and different chemical substances which usually are originated from microorganisms like fungi, themselves.

This study also indicates the high diversity of microorganisms in soil as the highest number of the most predominant genus distribution was less than 5%. The presence of multiple genera and high diversity of the species within the soil could be one of the reasons for high soil sustainability as an external or internal influence, for instance, suppression of one or few bacterial genera probably will not affect the whole microbiome itself.

Soil is one of the most favorable settings for acquisition and selection of antimicrobial resistance, due to the abundance of antibiotics-producing microorganisms. Chemicals that are used in conventional farming have potential to induce resistance development (Kleiner et al., 2007). On the other hand, during organic farming manure as a fertilizer is used, therefore antimicrobial resistant bacteria originated from gut of the animals may spread into soil ecosystems and increase resistance (Li B. et al., 2015; McKinney et al., 2018). Different animal pathogens as well as commensal microbiota have potential for horizontal transmission of the resistance genes (von Wintersdorff et al., 2016) therefore, resistance transfer of antimicrobial resistant bacteria may occur in both directions – from animals to soil and vice versa – from soil to animals because soils also contain an autochtonous bacterial microbiota which harbors resistance genes (Rizzo et al., 2013; Marti et al., 2014). Once bacteria have acquired ARGs, they may exist in the environment for a long time, even after the selection pressure (Tamminen et al., 2012).

In this study we have detected only single genes encoding antimicrobial resistance from the DNA of soil microbiomes in all tested samples regardless of the farming system. They conferred resistance mechanisms to β-lactams, aminoglycosides, tetracycline and erythromycin. All these antimicrobials are used in human and veterinary medicine and our previous studies demonstrated that animal microbiota contain a wide variety of clinically important genes encoding antimicrobial resistance (Seputiene et al., 2012; Klimiene et al., 2016). There was no recorded history about the origin of the manure in the organic farming fields, therefore we could expect the variety of resistance genes to differ between the various animal farms depending on the treatment of animals, which could be reflected in the amount of resistance genes reaching the fields with manure.

The recent data from functional metagenomics reveals novel genetic determinants that could be potentially foreseen as indicators of soil resistome and its dynamics (Torres-Cortés et al., 2011; McGarvey et al., 2012; Wichmann et al., 2014). We have shown in our study that *arr*-like 1 gene conferring rifampin resistance was present in all soils, whereas other determinants were sporadic or absent. Moreover, all soil samples except two contained *tetM* gene, which has been reported to be abundantly present in the microbiomes of various origin and the gene was proposed to be an indicator for the co-occurrence of other antibiotic resistance genes (Li B. et al., 2015).

Recent soil metagenome studies show the relative dominance of determinants encoding bacterial efflux systems among ARGs compared to other resistance mechanisms such as enzyme-mediated drug modification or drug target binding (Li B. et al., 2015; Van Goethem et al., 2018). We therefore analyzed the EP activity of cultivable isolates of three genera (*Pseudomonas, Stenotrophomonas* and *Chryseobacterium*). The genera were chosen as they are increasingly associated with infections and raise a threat due to their high intrinsic resistance (Ho et al., 2010; Brooke, 2012; Mukerji et al., 2016). *Pseudomonas aeruginosa* has been continuously shown to use RND EPs to counteract antibiotics, the presence of the same mechanisms are also shown for environmental *Pseudomonas* strains (Poole, 2001). Our research confirms that resistant isolates of soil origin also efficiently use RND EP. *Stenotrophomonas* spp. environmental strains have been demonstrated to possess similar ARGs as clinical strains (Youenou et al., 2015; Wang et al., 2018). In our EP inibition test we have observed similar action of EP in *S. maltophilia* and *Stenotrophomonas* of other species, indicating the EP that are present (the RND and ABC in our study) are able to cause resistance. Interestingly, we have found that efflux is also used by *Chryseobacterium* spp. of soil origin, thought these bacteria were mostly know to be resistant by drug modification mechanisms (Lin et al., 2012).

Hence, our resistance mechanisms studies of the most prevalent groups of soil cultivable bacteria from soils of different farming systems support the significant role of RND and ABC EPs in mediating resistance. The efficient efflux-mediated mechanisms in soil bacteria, therefore, might present a source for multidrug resistance spread including horizontal transfer (Dolejska et al., 2013; Walsh and Duffy, 2013).

According to this study it may be outlined that soil microbiota is a stable component as it were detected similar composition of microorganisms in soil both in organic as well as in conventional farming systems with similar soil structure and pH. The different amount of phosphorus in soils had no influence on bacterial variety at a genera level although more investigations would be useful to investigate changes among separate species. During evolution microorganisms adapted to survive in ecosystems independently of certain changes and probably serve as a buffer for ecological niches. It is unclear, however, what level of intensity can change microbial composition but current conventional farming in Central Europe demonstrates acceptable level of intensity for one of the most important ecological component of soils. Analysis of antimicrobial resistance in soils demonstrates that microorganisms did not acquire a plethora of genetic determinants encoding resistance mechanisms to the antimicrobials used in human and animal medicine as only a small number and low variety of clinically important genes encoding resistance to those antimicrobials were detected. However, the antibiotic resistance of the cultivable agricultural soil bacteria, including clinically relevant species, is largely mediated by the drug efflux mechanisms.

## Author Contributions

JA, JS, MR, EB, ES, IK, and VK designed the experiments. JA, JS, RK, EB, and RŠ performed the experiments. JA, JS, RK, EB, and SK analyzed the data. JA, ES, EB, and MR wrote the manuscript.

## Conflict of Interest Statement

The authors declare that the research was conducted in the absence of any commercial or financial relationships that could be construed as a potential conflict of interest.
