# Supplementary material for: Microbial Diversity and Antimicrobial Resistance Profile in Microbiota From Soils of Conventional and Organic Farming Systems
Source: Front Microbiol. 2019 Apr 26;10:892. doi: 10.3389/fmicb.2019.00892 (PMC6498881; doi:10.3389/fmicb.2019.00892)
Supplement: Supplementary file 2 [file Table_2.DOCX]

Supplementary Material

**Microbial diversity and antimicrobial resistance profile in microbiota from soils of conventional and organic farming systems**

Julija Armalytė, Jūratė Skerniškytė, Elena Bakienė, Renatas Krasauskas, Rita Šiugždinienė, Violeta Kareivienė, Sigita Kerzienė, Irena Klimienė, Edita Sužiedėlienė, Modestas Ružauskas*

*** Correspondence:** modestas.ruzauskas@lsmuni.lt

**Table S2.** Primers used in the study.

| **Gene** | **Primer** | **Primer sequence 5‘-3‘** | **Amplicon size, bp** | **Reference** |
| --- | --- | --- | --- | --- |
| **rRNA** |  |  |  |  |
| 16S rRNA | 27F | AGAGTTTGATYMTGGCTCAG | 525 | (Kim et al., 2012) |
|  | 515R | TTACCGCGGCKGCTGGCAC |  |  |
|  | Frrs | GATTAGATACCCTGGTAGTCC | 319 | This work |
|  | Rrrs | GTTGCGGGACTTAACCCAAC |  |  |
|  |  |  |  |  |
| **β lactams** |  |  |  |  |
| *ctx-M* | FCTXU | ATG TGC AGY ACC AGT AAR GTK ATG GC | 593 | (Eckert et al., 2006) |
|  | RCTXU | TGG GTR AAR TAR GTS ACC AGA AYC AGC GG |  |  |
| *oxa1* | F1OXA1 | GAT ATC TCT ACT GTT GCA TCT C | 619 | (Fang et al., 2008) |
|  | R1OXA1 | AAT AAA CCC TTC AAA CCA TCC G |  |  |
| *oxa2* | FOXA2 | GCC AAA GGC ACG ATA GTT GT | 600 | (Fang et al., 2008) |
|  | ROXA2 | TCA TCC ATC CTG TTT GGC GT |  |  |
| *oxa23* | FOXA23 | TTA GCA CCT ATG GTA ATG CTC T | 526 | (Fang et al., 2008) |
|  | ROXA23 | TCC ACC CAA CCA GTC AAC CA |  |  |
| *shv* | F1SHV | AGG ATT GAC TGC CTT TTT GCG | 392 | (Fang et al., 2008) |
|  | R1SHV | ATT TGC TGA TTT CGC TCG GC |  |  |
| *blaL1* | blaL1_Sm_F | ACCACACCTGGCAGATCGG | 346 | This work |
|  | blaL1_Sm_R | TCGCCATCCATGATGATGCG |  |  |
| *blaL2* | blaL2_Sm_F | GAGCGCTTCCCGATGTGCA | 526 | This work |
|  | blaL2_Sm_R | CATTGCTGCCGGTCTTGTC | 346 |  |
|  |  |  |  |  |
| **Aminoglycosides** |  |  |  |  |
| *ant(6)-I (aadE)* | FANT-6 | AGCCGGAGGATATGGAATTAT | 463 | (Ramirez and Tolmasky, 2010) |
|  | RANT-6 | TTCATAGGAATCCATCCGGTA |  |  |
| *ant(3”)-Ia (aadA1)* | FANT-3 | CGC CGA AGT ATC GAC TCA AC | 559 | (Chen et al., 2004) |
|  | RANT-3 | GCG GGA CAA CGT AAG CAC TA |  |  |
| *ant(3‘‘)Ib (aadA2)* | FAAD2 | GCTCAATGACCTTATGAAGGC | 379 | (Šeputiene et al., 2006) |
|  | RAAD2 | GCGGGACAACGTAAGCACTA |  |  |
| *ant(2’’)Ia (aadB)* | FANTIa | GAGCGAAATCTGCCGCTCTG | 320 | (Vakulenko et al., 2003) |
|  | RANTIa | CTGTTACAACGGACTGGCC |  |  |
| *aph(6)-I (strB)* | FAPH -6 | ATC GTC AAG GGA TTG AAA CCT A | 510 | (Madsen et al., 2000) |
|  | RAPH -6 | GGA TCG TAG AAC ATA TTG GCG |  |  |
| *aph(3‘)Ia (aphA1)* | FAPHI | ATGGGCTCGCGATAATGTC | 634 | Sheryl et al., 2008 |
|  | RAPHI | CTCACCGAGGCAGTTCCAT |  |  |
| *aph(3‘)IIa (aphA2)* | FAPHII | GAACAAGATGGATTGCACGC | 680 | (Maynard et al., 2003) |
|  | RAPHII | GCTCTTCAGCAATATCACGG |  |  |
| *aph(3”)-I* | F1APH-3 | CTT GGT GAT AAC GGC AAT TCC | 547 | (Madsen et al., 2000) |
|  | R1APH-3 | CCA ATC GCA GAT AGA AGG CAA |  |  |
| *aac(3)Iab(aacC1)* | FAAC3I | AGCAGCAACGATGTTAACGCA | 470 | (Ramirez and Tolmasky, 2010) |
|  | RAAC3I | CTGCGGGATCGTCACCGTA |  |  |
|  |  |  |  |  |
|  |  |  |  |  |
| *aac(6’)-Ib(aadA4)* | FAAC6b | AGT ACA GCA TCG TGA CCA ACA | 500 | (Machado et al., 2006) |
|  | RAAC6b | ATG TAC ACG GCT GGA CCA TC |  |  |
| *aac(3)IIa (aacC3)* | FAAC3IIa | GGTT CGG CCT GCT GAA TCA | 442 | (Ramirez and Tolmasky, 2010) |
|  | RAAC3IIa | AA GCC CAC GAC ACC TTC TC |  |  |
| *aac(3)IV* | FAAC3IV | GATGGGCCACTTGGACTGAT | 462 | (Chen et al., 2005) |
|  | RAAC3IV | GCGCTCACAGCAGTGGTCAT |  |  |
| *aac(6‘)Ib (aadA4)* | FAAC6b | AGTACAGCATCGTGACCAACA | 500 | (Galimand et al., 1993) |
|  | RAAC6b | ATGTACACGGCTGGACCATC |  |  |
|  |  |  |  |  |
| **Tetracyclines** |  |  |  |  |
| *tetA* | FTETA | GCT ACA TCC TGC TTG CCT TC | 210 | (Ng et al., 2001) |
|  | RTETA | CAT AGA TCG CCG TGA AGA GG |  |  |
| *tetB* | FTETB | TTG GTT AGG GGC AAG TTT TG | 659 | (Ng et al., 2001) |
|  | RTETB | GTA ATG GGC CAA TAA CAC CG |  |  |
| *tetC* | FTETC | CTT GAG AGC CTT CAA CCC AG | 418 | (Ng et al., 2001) |
|  | RTETC | ATG GTC GTC ATC TAC CTG CC |  |  |
| *tetD* | FTETD | AAA CCA TTA CGG CAT TCT GC | 787 | (Ng et al., 2001) |
|  | RTETD | GAC CGG ATA CAC CAT CCA TC |  |  |
| *tetM* | FTETM | GTG GAC AAA GGT ACA ACG AG | 406 | (Ng et al., 2001) |
|  | RTETM | CGG TAA AGT TCG TCA CAC AC |  |  |
|  |  |  |  |  |
| **Macrolides** |  |  |  |  |
| *ermA* | FermA | GAAGCGGTAAACCCCTCTG | 216 | (Seputiene et al., 2012) |
|  | RermA | ACCCAAAGCTCGTTGCAGAT |  |  |
| *ermB* | FermB | ATTGGAACAGGTAAAGGGCAT | 447 | (Seputiene et al., 2012) |
|  | RermB | ATCTGGAACATCTGTGGTATG |  |  |
| *ermC* | FermC | GAAATCGGCTCAGGAAAAGG | 293 | (Seputiene et al., 2012) |
| *mefAB* | FmefAB | AGTATCATTAATCACTAGTGCC | 347 | (Seputiene et al., 2012) |
|  | RmefAB | GTTCTTCTGGTACTAAAAGTGG |  |  |
|  |  |  |  |  |
| **Chloramphenicol** |  |  |  |  |
| *catI* | FCATI | CT ATA ACC AGA CCG TTC AGC T | 499 | This work |
|  | RCATI | TAA GCA TTC TGC CGA CAT GGA |  |  |
|  |  |  |  |  |
| **Quinolones** |  |  |  |  |
| *qnrA* | FqnrA | ATTTCTCACGCCAGGATTTG | 516 | (Gay et al., 2006) |
|  | RqnrA | GATCGGCAAAGGTTAGGTCA |  |  |
| *qnrB* | FqnrB | GATCGTGAAAGCCAGAAAGG | 469 | (Gay et al., 2006) |
|  | RqnrB | ACGATGCCTGGTAGTTGTCC |  |  |
| *qnrS* | FqnrS | ACGACATTCGTCAACTGCAA | 417 | (Gay et al., 2006) |
|  | RqnrS | TAAATTGGCACCCTGTAGGC |  |  |
| *qnrD* | FqnrD | CGAGATCAATTTACGGGGAAT | 582 | (Xia et al., 2010) |
|  | RqnrD | AACAAGCTGAAGCGCCTG |  |  |
| *qepA1* | FqepA1 | GCAGGTCCAGCAGCGGGTAG | 218 | (Yamane et al., 2008) |
|  | RqepA1 | CTTCCTGCCCGAGTATCGTG |  |  |
|  |  |  |  |  |
| **Glycopeptides** |  |  |  |  |
| *vanA* | FvanA | TCAGCTTTGCATGGCAAGTC | 520 | (Dutka-Malen et al., 1995) |
|  | RvanA | GCTCCTCTGCTGAAAGGTCT |  |  |
| *vanB* | FvanB | CGGCAGGACAATATGATGGA | 419 | (Dutka-Malen et al., 1995) |
|  | RvanB | GCTGTCAATCAGTGCAGGAA |  |  |
| *vanC1* | FvanC1 | GGTATCAAGGAAACCTC | 822 | (Dutka-Malen et al., 1995) |
|  | RvanC1 | CTTCCGCCATCATAGCT |  |  |
| *vanC2/3* | FvanC23 | CTCCTACGATTCTCTTG | 439 | (Dutka-Malen et al., 1995) |
|  | RvanC23 | CGAGCAAGACCTTTAAG |  |  |
| *vanD* | FvanD | CATCAGGAAGCACAGCC | 235 | (Domingo et al., 2005) |
|  | RvanD | GCTGCTGTCATCATGCG |  |  |
| *vanE* | FvanE | TGTAGGTTGTGGTATCGGA | 515 | (Domingo et al., 2005) |
|  | RvanE | ATTCTCGCTAATCCTTTGCA |  |  |
| *vanG* | FvanG | GATGAAATCGAACTGTCAAG | 270 | (Domingo et al., 2005) |
|  | RvanG | AATGCCTTTCATCATATTTGG |  |  |
|  |  |  |  |  |
| **Resistance genes of soil origin** |  |  |  |  |
|  |  |  |  |  |
| **β lactams** |  |  |  |  |
| *blaLRA-10^2^* | F408357 | TGCTBVAHCTSGGCACCTA | 639 | This work |
|  | R408357 | GTCGAGCCGGTCRMGBTG |  |  |
| *blaLRA-13^2^* | F408352 | TCGGYTCSHTSAGCAAGAC | 634 | This work |
|  | R408352 | TGYTCCCARATCAGRTCCTG |  |  |
| *bla*^3^ | F640467 | GARMTSGGWTCVATCAGCAA | 779 | This work |
|  | R640467 | GTBGARCCRGTYTTGTTGAT |  |  |
| *bla2*^4^ | F512902 | ACGCCGTTGACATGGAGAAT | 532 | This work |
|  | R512902 | TGCGGAGTTTCTGCCTTGAT |  |  |
|  |  |  |  |  |
| **Aminoglycosides** |  |  |  |  |
| *aac3*^1^ | F640468 | CTCAGCACCGCTCGACAC | 129 | This work |
|  | R640468 | ACGATNNBSGCGAAATARTC |  |  |
|  |  |  |  |  |
| **Tetracyclines** |  |  |  |  |
| *tetW*^4^ | F512988 | CAGAGCGTGGTTCAGTCTGT | 728 | This work |
|  | R512988 | TCCACTTCGCAACGCAAAAG |  |  |
| *tet4*^5^ | F924904 | RKCVGADCTGCTBGAGGA | 238 | This work |
|  | R924904 | SAGRTAYTGSGTBGTBAGCA |  |  |
|  |  |  |  |  |
| **Chloramphenicol** |  |  |  |  |
| *bcr/cfl*^6^ | F640469 | ACATGDWCGGBCGCAAGC | 577 | This work |
|  | R640469 | AABSHCASSGCRTTSAYCGAGAA |  |  |
|  |  |  |  |  |
| **Rifampicin** |  |  |  |  |
| *arr-like 1*^5^ | F924874 | ATBGCHAARTGCTAYGARGAHTTA | 230 | This work |
|  | R924874 | GCWAGTCCHGCYCCATT |  |  |
| *arr-like 2*^5^ | F924876 | GCMAARTSSTAYGARGAHTTA | 329 | This work |
|  | R924876 | CCYGGRAATTTYTTRTCKGTAA |  |  |
|  |  |  |  |  |
|  |  |  |  |  |
|  |  |  |  |  |
|  |  |  |  |  |

^1^PI: CBI71190.1, FN640468 (Torres-Cortés et al., 2011); ^2^(Allen et al., 2009); ^3^ PI: CBI71185.1, FN640467 (Torres-Cortés et al., 2011); ^4^(Wichmann et al., 2014); ^5^PI:AEI30227.1, JF924904; PI: AEI30165.1, JF924874; PI: AEI30168.1, JF924876 (McGarvey et al., 2012);

## References

Allen, H. K., Moe, L. A., Rodbumrer, J., Gaarder, A., and Handelsman, J. (2009). Functional metagenomics reveals diverse beta-lactamases in a remote Alaskan soil. *ISME J.* 3, 243–251. doi:10.1038/ismej.2008.86.

Chen, S., Zhao, S., McDermott, P. F., Schroeder, C. M., White, D. G., and Meng, J. (2005). A DNA microarray for identification of virulence and antimicrobial resistance genes in Salmonella serovars and Escherichia coli. *Mol. Cell. Probes* 19, 195–201. doi:10.1016/j.mcp.2004.11.008.

Chen, S., Zhao, S., White, D. G., Schroeder, C. M., Lu, R., Yang, H., et al. (2004). Characterization of Multiple-Antimicrobial-Resistant Salmonella Serovars Isolated from Retail Meats. *Appl. Environ. Microbiol.* 70, 1–7. doi:10.1128/AEM.70.1.1-7.2004.

Domingo, M.-C., Huletsky, A., Giroux, R., Boissinot, K., Picard, F. J., Lebel, P., et al. (2005). High prevalence of glycopeptide resistance genes vanB, vanD, and vanG not associated with enterococci in human fecal flora. *Antimicrob. Agents Chemother.* 49, 4784–4786. doi:10.1128/AAC.49.11.4784-4786.2005.

Dutka-Malen, S., Evers, S., and Courvalin, P. (1995). Detection of glycopeptide resistance genotypes and identification to the species level of clinically relevant enterococci by PCR. *J. Clin. Microbiol.* 33, 1434.

Eckert, C., Gautier, V., and Arlet, G. (2006). DNA sequence analysis of the genetic environment of various blaCTX-M genes. *J. Antimicrob. Chemother.* 57, 14–23. doi:10.1093/jac/dki398.

Fang, H., Ataker, F., Hedin, G., and Dornbusch, K. (2008). Molecular Epidemiology of Extended-Spectrum β-Lactamases among Escherichia coli Isolates Collected in a Swedish Hospital and Its Associated Health Care Facilities from 2001 to 2006. *J. Clin. Microbiol.* 46, 707–712. doi:10.1128/JCM.01943-07.

Galimand, M., Lambert, T., Gerbaud, G., and Courvalin, P. (1993). Characterization of the aac(6’)-Ib gene encoding an aminoglycoside 6’-N-acetyltransferase in Pseudomonas aeruginosa BM2656. *Antimicrob. Agents Chemother.* 37, 1456–1462.

Gay, K., Robicsek, A., Strahilevitz, J., Park, C. H., Jacoby, G., Barrett, T. J., et al. (2006). Plasmid-mediated quinolone resistance in non-Typhi serotypes of Salmonella enterica. *Clin. Infect. Dis. Off. Publ. Infect. Dis. Soc. Am.* 43, 297–304. doi:10.1086/505397.

Kim, S. H., Jeong, H. S., Kim, Y. H., Song, S. A., Lee, J. Y., Oh, S. H., et al. (2012). Evaluation of DNA Extraction Methods and Their Clinical Application for Direct Detection of Causative Bacteria in Continuous Ambulatory Peritoneal Dialysis Culture Fluids from Patients with Peritonitis by Using Broad-Range PCR. *Ann. Lab. Med.* 32, 119–125. doi:10.3343/alm.2012.32.2.119.

Machado, E., Coque, T. M., Cantón, R., Baquero, F., Sousa, J. C., Peixe, L., et al. (2006). Dissemination in Portugal of CTX-M-15-, OXA-1-, and TEM-1-producing Enterobacteriaceae strains containing the aac(6’)-Ib-cr gene, which encodes an aminoglycoside- and fluoroquinolone-modifying enzyme. *Antimicrob. Agents Chemother.* 50, 3220–3221. doi:10.1128/AAC.00473-06.

Madsen, L., Aarestrup, F. M., and Olsen, J. E. (2000). Characterisation of streptomycin resistance determinants in Danish isolates of Salmonella Typhimurium. *Vet. Microbiol.* 75, 73–82. doi:10.1016/S0378-1135(00)00207-8.

Maynard, C., Fairbrother, J. M., Bekal, S., Sanschagrin, F., Levesque, R. C., Brousseau, R., et al. (2003). Antimicrobial resistance genes in enterotoxigenic Escherichia coli O149:K91 isolates obtained over a 23-year period from pigs. *Antimicrob. Agents Chemother.* 47, 3214–3221.

McGarvey, K. M., Queitsch, K., and Fields, S. (2012). Wide variation in antibiotic resistance proteins identified by functional metagenomic screening of a soil DNA library. *Appl. Environ. Microbiol.* 78, 1708–1714. doi:10.1128/AEM.06759-11.

Ng, L. K., Martin, I., Alfa, M., and Mulvey, M. (2001). Multiplex PCR for the detection of tetracycline resistant genes. *Mol. Cell. Probes* 15, 209–215. doi:10.1006/mcpr.2001.0363.

Ramirez, M. S., and Tolmasky, M. E. (2010). Aminoglycoside modifying enzymes. *Drug Resist. Updat. Rev. Comment. Antimicrob. Anticancer Chemother.* 13, 151–171. doi:10.1016/j.drup.2010.08.003.

Seputiene, V., Bogdaite, A., Ruzauskas, M., and Suziedeliene, E. (2012). Antibiotic resistance genes and virulence factors in Enterococcus faecium and Enterococcus faecalis from diseased farm animals: pigs, cattle and poultry. *Pol. J. Vet. Sci.* 15, 431–438.

Šeputiene, V., Ružauskas, M., Žlabys, P., and Sužiedėlienė, E. (2006). Characterisation of streptomycin resistance determinants in Lithuanian Escherichia coli isolates. *Biologija* 2, 14–17.

Torres-Cortés, G., Millán, V., Ramírez-Saad, H. C., Nisa-Martínez, R., Toro, N., and Martínez-Abarca, F. (2011). Characterization of novel antibiotic resistance genes identified by functional metagenomics on soil samples. *Environ. Microbiol.* 13, 1101–1114. doi:10.1111/j.1462-2920.2010.02422.x.

Vakulenko, S. B., Donabedian, S. M., Voskresenskiy, A. M., Zervos, M. J., Lerner, S. A., and Chow, J. W. (2003). Multiplex PCR for detection of aminoglycoside resistance genes in enterococci. *Antimicrob. Agents Chemother.* 47, 1423–1426.

Wichmann, F., Udikovic-Kolic, N., Andrew, S., and Handelsman, J. (2014). Diverse antibiotic resistance genes in dairy cow manure. *mBio* 5, e01017. doi:10.1128/mBio.01017-13.

Xia, L.-N., Li, L., Wu, C.-M., Liu, Y.-Q., Tao, X.-Q., Dai, L., et al. (2010). A survey of plasmid-mediated fluoroquinolone resistance genes from Escherichia coli isolates and their dissemination in Shandong, China. *Foodborne Pathog. Dis.* 7, 207–215. doi:10.1089/fpd.2009.0378.

Yamane, K., Wachino, J., Suzuki, S., and Arakawa, Y. (2008). Plasmid-mediated qepA gene among Escherichia coli clinical isolates from Japan. *Antimicrob. Agents Chemother.* 52, 1564–1566. doi:10.1128/AAC.01137-07.
