# Supplementary material for: Microbial Diversity and Antimicrobial Resistance Profile in Microbiota From Soils of Conventional and Organic Farming Systems
Source: Front Microbiol. 2019 Apr 26;10:892. doi: 10.3389/fmicb.2019.00892 (PMC6498881; doi:10.3389/fmicb.2019.00892)
Supplement: Supplementary file 3 [file Table_3.DOCX]

Supplementary Material

Microbial diversity and antimicrobial resistance profile in microbiota from soils of conventional and organic farming systems

JulijaArmalytė, Jūratė Skerniškytė, Elena Bakienė, Renatas Krasauskas, Rita Šiugždinienė, Violeta Kareivienė, Sigita Kerzienė, Irena Klimienė, Edita Sužiedėlienė, Modestas Ružauskas*

*** Correspondence:** modestas.ruzauskas@lsmuni.lt

**Table S3.** The most prevalent genera (> 0.5 %) of bacteria from conventional and organic wheat farming soils.

| Genus | Wheat farming soil | |
| --- | --- | --- |
|  | **Organic** | **Conventional** |
| *Acidobacterium** | 4.06% | 3.57% |
| *Bacillus** | 2.55% | 3.28% |
| unclassified  *Alphaproteobacteri** | 1.92% | 1.74% |
| unclassified   *Acidobacteriaceae** | 1.72% | 1.39% |
| unclassified Candidatus *Saccharibacteria** | 1.72% | 1.82% |
| unclassified *Betaproteobacteria** | 1.64% | 1.67% |
| *Holophaga** | 1.59% | 0.87% |
| *Hyphomicrobium** | 1.51% | 1.09% |
| *Arthrobacter** | 1.49% | 1.71% |
| *Gemmatimonas** | 1.49% | 1.91% |
| *Flavobacterium** | 1.47% | 1.11% |
| *Nocardioides** | 1.45% | 1.04% |
| unclassified *Deltaproteobacteria* | 1.25% | 1.14% |
| unclassified *Planctomycetales* | 1.12% | 0.97% |
| *Clostridium* | 1.00% | 0.70% |
| *Geobacter* | 1.00% | 0.65% |
| unclassified *Actinobacteria* | 0.99% | 0.83% |
| *Ilumatobacter* | 0.97% | 0.40% |
| *Nitrospira** | 0.97% | 1.17% |
| unclassified *Gammaproteobacteria* | 0.95% | 0.99% |
| *Sphingomonas** | 0.93% | 1.17% |
| *Mycobacterium* | 0.86% | 0.77% |
| *Chitinophaga* | 0.82% | 0.63% |
| unclassified *Acidobacteriales* | 0.82% | 0.48% |
| *Longilinea* | 0.81% | 0.74% |
| unclassified *Conexibacteraceae* | 0.81% | 0.33% |
| unclassified *Sphaerobacteridae* | 0.80% | 0.69% |
| unclassified *Holophagaceae* | 0.78% | 0.52% |
| *Gaiella* | 0.75% | 0.23% |
| unclassified *Actinobacteridae* | 0.75% | 0.41% |
| unclassified *Myxococcales* | 0.75% | 0.65% |
| unclassified *Rubrobacteridae* | 0.75% | 0.36% |
| unclassified *Bacteria* | 0.65% | 1.40% |
| unclassified *Verrucomicrobiales* | 0.65% | 0.63% |
| *Knoellia* | 0.61% | 0.36% |
| *Iamia* | 0.57% | 0.27% |
| *Bradyrhizobium* | 0.56% | 0.64% |
| *Chloroflexus* | 0.55% | 0.37% |
| unclassified *Gemmatimonadales* | 0.55% | 0.80% |
| *Rhodoplanes* | 0.54% | 0.43% |
| *Streptomyces* | 0.54% | 0.82% |
| unclassified *Pirellulaceae* | 0.54% | 0.77% |
| unclassified *Chthoniobacteraceae* | 0.53% | 0.41% |
| unclassified *Chitinophagaceae* | 0.44% | 0.76% |
| unclassified *Gemmatimonadaceae* | 0.41% | 0.61% |
| *Pedobacter* | 0.38% | 0.95% |
| unclassified *Rhizobiales* | 0.38% | 0.55% |
| *Paenibacillus* | 0.30% | 0.64% |
| *Rhizobium* | 0.26% | 0.54% |
| *Devosia* | 0.18% | 0.89% |
| *Sporosarcina* | 0.17% | 0.53% |
| *Agrobacterium* | 0.13% | 0.63% |
| *Ferruginibacter* | 0.12% | 0.53% |

* The genera were found to be significantly different in the two soils (*p* < 0.001)
