# Supplementary material for: Microbial Diversity and Antimicrobial Resistance Profile in Microbiota From Soils of Conventional and Organic Farming Systems
Source: Front Microbiol. 2019 Apr 26;10:892. doi: 10.3389/fmicb.2019.00892 (PMC6498881; doi:10.3389/fmicb.2019.00892)
Supplement: Supplementary file 4 [file Data_Sheet_1.PDF]

All bacterial species from the soil of organic farming

Species	Number of reads	Relative abundance
unclassified Acidobacterium	3792	4.06%
unclassified Acidobacteriaceae	1609	1.72%
unclassified Holophaga	1483	1.59%
unclassified Gemmatimonas	1398	1.49%
unclassified Betaproteobacteria	1385	1.48%
unclassified Hyphomicrobium	1374	1.47%
unclassified Alphaproteobacteria	1325	1.42%
unclassified Candidatus Saccharibacteria	1247	1.33%
unclassified Deltaproteobacteria	1165	1.24%
unclassified Bacillus	1126	1.2%
unclassified Planctomycetales	1047	1.12%
unclassified Geobacter	928	0.99%
unclassified Ilumatobacter	907	0.97%
unclassified Actinobacteria	905	0.97%
unclassified Gammaproteobacteria	839	0.9%
unclassified Nitrospira	808	0.86%
unclassified Acidobacteriales	769	0.82%
unclassified Conexibacteraceae	760	0.81%
unclassified Chitinophaga	760	0.81%
unclassified Longilinea	756	0.81%
unclassified Sphaerobacteridae	750	0.8%
unclassified Holophagaceae	736	0.78%
unclassified Arthrobacter	716	0.76%
unclassified Myxococcales	707	0.75%
unclassified Rubrobacteridae	701	0.75%
unclassified Actinobacteridae	698	0.74%
unclassified Gaiella	697	0.74%
Arthrobacter pascens	653	0.7%
unclassified Nocardioides	613	0.65%
unclassified Bacteria	609	0.65%
unclassified Verrucomicrobiales	606	0.65%
Bacillus longiquaesitum	579	0.62%
unclassified rosids	566	0.6%
unclassified Plectosphaerellaceae	552	0.59%
unclassified Iamia	534	0.57%
unclassified Chloroflexus	521	0.55%
unclassified Gemmatimonadales	514	0.55%
unclassified Flavobacterium	512	0.54%
unclassified Pirellulaceae	508	0.54%
unclassified Rhodoplanes	507	0.54%
unclassified Chthoniobacteraceae	499	0.53%
Flavobacterium succinicans	495	0.53%
unclassified Gaiellaceae	492	0.52%
unclassified Candidatus Solibacter	480	0.51%
unclassified Mycobacterium	474	0.5%
alpha proteobacterium	465	0.49%
unclassified Caldilinea	462	0.49%
unclassified Sphingomonas	447	0.47%
unclassified Holophagales	444	0.47%
Bradyrhizobium elkanii	433	0.46%

Species	Number of reads	Relative abundance
unclassified Pseudonocardia	430	0.46%
unclassified Pedomicrobium	425	0.45%
unclassified Conexibacter	414	0.44%
unclassified Chitinophagaceae	412	0.44%
Knoellia subterranea	396	0.42%
unclassified Gemmatimonadaceae	390	0.41%
unclassified Solirubrobacter	388	0.41%
unclassified Frankia	383	0.41%
unclassified Actinomycetales	379	0.4%
unclassified Hypocreomycetidae	359	0.38%
metal-contaminated soil	357	0.38%
unclassified Rhizobiales	355	0.38%
unclassified Anaerolinaceae	346	0.37%
unclassified Burkholderia	343	0.36%
unclassified Desulfuromonadales	335	0.35%
Unclassified	332	0.35%
unclassified Pirellula	324	0.34%
unclassified Terrimonas	318	0.34%
unclassified Gemmata	313	0.33%
unclassified Anaerolineae	308	0.33%
unclassified Flexibacter	307	0.32%
Clostridium bowmanii	300	0.32%
unclassified Aciditerrimonas	298	0.31%
unclassified Clostridium	294	0.31%
unclassified Planctomycetaceae	293	0.31%
unclassified Burkholderiaceae	290	0.31%
unclassified Levilinea	284	0.3%
unclassified Anaerolineaceae	281	0.3%
unclassified Acidobacteriia	279	0.29%
unclassified Acidimicrobidae	271	0.29%
unclassified Geobacteraceae	267	0.28%
unclassified Cytophagaceae	266	0.28%
unclassified Steroidobacter	266	0.28%
unclassified Nitrosospira	264	0.28%
unclassified Clostridia	256	0.27%
unclassified Syntrophobacteraceae	255	0.27%
Nocardioides mesophilus	255	0.27%
unclassified Iamiaceae	254	0.27%
unclassified Ferrimicrobium	252	0.27%
unclassified Saccharibacillus	245	0.26%
unclassified Candidatus Nitrososphaera	245	0.26%
unclassified Clostridiales	243	0.26%
unclassified Verrucomicrobiaceae	240	0.25%
unclassified Thermoanaerobacterales	236	0.25%
unclassified Lysobacter	235	0.25%
unclassified Rhizobium	226	0.24%
unclassified Alcaligenaceae	224	0.24%
unclassified Vitaceae	223	0.23%
unclassified Anaerolineales	222	0.23%
unclassified Nitrosomonadaceae	218	0.23%

Species	Number of reads	Relative abundance
unclassified Acidimicrobiaceae	213	0.22%
unclassified Chlamydiales	213	0.22%
unclassified Pseudomonadales	209	0.22%
unclassified Methylibium	209	0.22%
unclassified Dehalococcoidaceae	208	0.22%
unclassified Burkholderiales	207	0.22%
unclassified Roseiflexus	206	0.22%
unclassified Nocardioidaceae	206	0.22%
unclassified Niastella	206	0.22%
unclassified Gemmataceae	204	0.21%
unclassified Chondromyces	202	0.21%
unclassified Nitrospirales	201	0.21%
unclassified Pseudolabrys	201	0.21%
unclassified Pseudomonadaceae	200	0.21%
unclassified Arenimonas	200	0.21%
unclassified Gemmatimonadetes	199	0.21%
unclassified Thermoleophilales	198	0.21%
Sphingomonas jaspsi	196	0.21%
unclassified Opitutus	195	0.2%
unclassified Verticillium	195	0.2%
unclassified Solirubrobacteraceae	195	0.2%
unclassified Acidothermus	194	0.2%
unclassified Methylosinus	193	0.2%
Bacillus muralis	192	0.2%
unclassified Sphaerobacter	191	0.2%
unclassified Flavisolibacter	190	0.2%
unclassified Planctomycetia	185	0.19%
unclassified Thermoleophilum	184	0.19%
unclassified Citricoccus	181	0.19%
unclassified Planctomyces	179	0.19%
unclassified Caldilineaceae	179	0.19%
Flavobacterium hydatis	178	0.19%
unclassified Kouleothrix	177	0.18%
unclassified Rhodocyclales	176	0.18%
unclassified Bacillales	176	0.18%
unclassified Knoellia	173	0.18%
unclassified Chloroflexales	173	0.18%
unclassified Thiorhodospira	173	0.18%
unclassified Haliangium	172	0.18%
unclassified Pedobacter	171	0.18%
unclassified Actinomyces	169	0.18%
unclassified Haliangiaceae	167	0.17%
unclassified Chloroflexi	165	0.17%
unclassified Marmoricola	165	0.17%
unclassified Bacilli	163	0.17%
unclassified Comamonadaceae	161	0.17%
unclassified Byssovorax	161	0.17%
unclassified Hydrogenophaga	160	0.17%
unclassified Rhodospirillaceae	160	0.17%
unclassified Adhaeribacter	159	0.17%

Species	Number of reads	Relative abundance
unclassified Dongia	157	0.16%
unclassified Phycisphaerales	156	0.16%
unclassified Haliscomenobacter	154	0.16%
unclassified Sphingobacteriales	151	0.16%
beta proteobacterium	150	0.16%
unclassified Solirubrobacterales	150	0.16%
Streptomyces reticuliscabiei	149	0.15%
unclassified Polyangiaceae	148	0.15%
unclassified Dehalococcoidetes	148	0.15%
Bacillus flexus	146	0.15%
unclassified Paenibacillus	145	0.15%
unclassified Dehalococcoidales	144	0.15%
unclassified Hyphomicrobiaceae	136	0.14%
unclassified Rhizobiaceae	133	0.14%
Streptomyces mirabilis	132	0.14%
unclassified Nordella	132	0.14%
unclassified Gemmatales	131	0.14%
unclassified Roseiflexales	131	0.14%
unclassified Geodermatophilaceae	129	0.13%
unclassified Microvirga	128	0.13%
unclassified Cyclobacteriaceae	128	0.13%
unclassified Pirellulales	127	0.13%
unclassified Flavobacteriales	127	0.13%
unclassified Pedosphaerales	124	0.13%
unclassified Mesorhizobium	122	0.13%
Nocardioides islandensis	122	0.13%
unclassified Xanthomonadaceae	121	0.12%
unclassified Bacillaceae	120	0.12%
unclassified Oxalobacteraceae	119	0.12%
Mesorhizobium amorphae	118	0.12%
unclassified Streptomyces	117	0.12%
unclassified Ferruginibacter	117	0.12%
unclassified Xanthomonadales	116	0.12%
Variovorax paradoxus	114	0.12%
Pseudomonas veronii	114	0.12%
unclassified Sphaerobacteraceae	114	0.12%
unclassified Sphingobacteriaceae	112	0.12%
unclassified Nakamurella	112	0.12%
unclassified Planctomycetes	112	0.12%
Kaistobacter terrae	111	0.11%
unclassified Opitutaceae	110	0.11%
unclassified Sporichthya	109	0.11%
unclassified Filomicrobium	107	0.11%
Povalibacter uvarum	104	0.11%
Bosea genosp.	103	0.11%
unclassified Legionellales	98	0.1%
unclassified Geothrix	98	0.1%
unclassified Janthinobacterium	98	0.1%
unclassified Solibacterales	97	0.1%
unclassified Thermomicrobia	96	0.1%

Species	Number of reads	Relative abundance
Rhizocola hellebori	95	0.1%
unclassified Fibrobacteria	95	0.1%
unclassified Bellilinea	95	0.1%
unclassified Bradyrhizobium	93	0.09%
unclassified Variovorax	92	0.09%
unclassified Nitrosomonadales	92	0.09%
unclassified Rubrivivax	91	0.09%
Sphingomonas kaistensis	91	0.09%
unclassified Luteolibacter	91	0.09%
Terrisporobacter glycolicus	90	0.09%
Nitrospira japonica	90	0.09%
unclassified Geothermobacter	89	0.09%
unclassified Syntrophobacterales	89	0.09%
unclassified Neisseriaceae	89	0.09%
Agromyces ramosus	89	0.09%
unclassified Sphingobacteriia	89	0.09%
unclassified Prolixibacter	88	0.09%
unclassified Desulfovibrio	88	0.09%
unclassified Cytophagales	86	0.09%
Clostridium perfringens	85	0.09%
Agrobacterium larrymoorei	85	0.09%
unclassified Rhodospirillales	85	0.09%
unclassified Coxiellaceae	84	0.09%
Nocardioides halotolerans	84	0.09%
unclassified Devosia	83	0.08%
unclassified Sordariomycetes	83	0.08%
unclassified Kribbella	83	0.08%
Bacillus thuringiensis	82	0.08%
unclassified Spartobacteria	82	0.08%
unclassified Pseudomonas	82	0.08%
unclassified Thermoleophilaceae	82	0.08%
unclassified Povalibacter	80	0.08%
unclassified Ohtaekwangia	80	0.08%
unclassified Pedosphaeraceae	79	0.08%
Mycobacterium vaccae	79	0.08%
unclassified Rubrobacterales	78	0.08%
unclassified Flavitalea	78	0.08%
unclassified Pelobacter	78	0.08%
Skermanella aerolata	78	0.08%
unclassified Streptophyta	78	0.08%
unclassified Azohydromonas	77	0.08%
unclassified Thiobacter	77	0.08%
Kurthia gibsonii	76	0.08%
unclassified Bdellovibrionaceae	76	0.08%
unclassified Anaeromyxobacter	74	0.07%
Nakamurella flavida	74	0.07%
unclassified Acetobacteraceae	74	0.07%
unclassified Flavobacteriaceae	73	0.07%
unclassified Sphingomonadales	72	0.07%
Janthinobacterium agaricidamnosum	72	0.07%

Species	Number of reads	Relative abundance
unclassified Thiotrichaceae	71	0.07%
unclassified Afifella	71	0.07%
Chelatococcus asaccharovorans	70	0.07%
unclassified Saprospiraceae	70	0.07%
unclassified Acidobacteria	70	0.07%
unclassified Micromonosporaceae	67	0.07%
unclassified Lysinibacillus	66	0.07%
Microbacterium chocolatum	65	0.06%
unclassified Bauldia	65	0.06%
unclassified Sinobacteraceae	65	0.06%
unclassified Bdellovibrionales	64	0.06%
Clostridium ruminantium	63	0.06%
unclassified Alteromonadales	63	0.06%
unclassified Paenibacillaceae	63	0.06%
unclassified Entotheonellaceae	63	0.06%
unclassified Thiobacillus	63	0.06%
unclassified Leptothrix	62	0.06%
unclassified Flavobacteriia	62	0.06%
unclassified Caldilineales	62	0.06%
unclassified Rhodocyclaceae	62	0.06%
Streptosporangium amethystogenes	62	0.06%
unclassified Desulfobacteraceae	62	0.06%
unclassified Massilia	62	0.06%
unclassified Nitrospiraceae	60	0.06%
unclassified Nannocystis	60	0.06%
unclassified Rhodopirellula	59	0.06%
unclassified Georgfuchsia	59	0.06%
Mycobacterium frederiksbergense	58	0.06%
Agromyces humatus	58	0.06%
unclassified Zavarzinella	58	0.06%
unclassified Rickettsiales	58	0.06%
unclassified Cupriavidus	57	0.06%
unclassified Desulfitobacterium	57	0.06%
Devosia ginsengisoli	57	0.06%
unclassified Streptomycetaceae	56	0.06%
unclassified Solibacteraceae	56	0.06%
unclassified Beggiatoa	56	0.06%
unclassified Cystobacteraceae	56	0.06%
unclassified Prosthecobacter	56	0.06%
unclassified Blastochloris	55	0.05%
unclassified Cryomorphaceae	55	0.05%
Ensifer adhaerens	55	0.05%
unclassified Rhodococcus	54	0.05%
unclassified Amaricoccus	54	0.05%
unclassified Modestobacter	54	0.05%
unclassified Blastococcus	53	0.05%
Nocardioides iriomotensis	53	0.05%
unclassified Methylobacterium	53	0.05%
unclassified Polyangium	53	0.05%
unclassified Cellvibrio	53	0.05%

Species	Number of reads	Relative abundance
unclassified Sporosarcina	53	0.05%
unclassified Thiohalospira	53	0.05%
Cellulomonas xylanilytica	52	0.05%
Solirubrobacter ginsenosidimutans	52	0.05%
unclassified Derxia	52	0.05%
unclassified Bosea	52	0.05%
unclassified Sporichthyaceae	52	0.05%
unclassified Kaistobacter	52	0.05%
unclassified Micrococcaceae	51	0.05%
Bacillus fumarioli	51	0.05%
Flavobacterium limicola	51	0.05%
unclassified Verrucomicrobium	50	0.05%
unclassified Acidovorax	50	0.05%
unclassified Caldilineae	50	0.05%
unclassified Acidimicrobiales	50	0.05%
unclassified Pilimelia	50	0.05%
Lysobacter oligotrophicus	49	0.05%
unclassified Dokdonella	49	0.05%
unclassified Chloroflexaceae	49	0.05%
unclassified Chthoniobacter	49	0.05%
Marmoricola bigeumensis	48	0.05%
unclassified Microbacterium	48	0.05%
unclassified Kouleothrixaceae	48	0.05%
gamma proteobacterium	48	0.05%
unclassified Bacteriovoracaceae	48	0.05%
Marmoricola aequoreus	48	0.05%
unclassified Legionellaceae	48	0.05%
unclassified Phaselicystidaceae	47	0.05%
unclassified Saprospirales	47	0.05%
unclassified Legionella	47	0.05%
unclassified Cytophagia	47	0.05%
unclassified Mycobacteriaceae	47	0.05%
unclassified Methylocystis	46	0.04%
unclassified Ruminococcaceae	46	0.04%
unclassified Saprospirae	46	0.04%
Clostridium butyricum	46	0.04%
Salinibacterium amurskyense	46	0.04%
unclassified Hirschia	46	0.04%
unclassified Bdellovibrio	46	0.04%
Blastococcus aggregatus	45	0.04%
unclassified Syntrophus	45	0.04%
unclassified Pasteuriaceae	45	0.04%
unclassified Porphyrobacter	45	0.04%
unclassified Microbacteriaceae	45	0.04%
unclassified Singulisphaera	44	0.04%
Nocardioides sediminis	44	0.04%
Micromonospora saelicesensis	44	0.04%
Sporosarcina soli	43	0.04%
Sphingomonas faeni	43	0.04%
unclassified Ferrithrix	43	0.04%

Species	Number of reads	Relative abundance
unclassified Oceanicella	43	0.04%
unclassified Clostridiaceae	42	0.04%
unclassified Phycisphaera	42	0.04%
unclassified Myxococcus	41	0.04%
unclassified Desulfovirga	41	0.04%
unclassified Roseomonas	41	0.04%
unclassified Parachlamydiaceae	41	0.04%
unclassified Micromonospora	40	0.04%
Flavobacterium tiangeerense	40	0.04%
unclassified Methylobacteriaceae	40	0.04%
unclassified Herpetosiphonales	40	0.04%
unclassified Alicyclobacillus	39	0.04%
unclassified Lachnoclostridium	39	0.04%
unclassified Sphingomonadaceae	38	0.04%
Kribbella karoonensis	38	0.04%
unclassified Ornithinicoccus	38	0.04%
unclassified Agromyces	38	0.04%
unclassified Opitutales	38	0.04%
Bacillus nealsonii	38	0.04%
unclassified Caldanaerobacter	38	0.04%
unclassified Verrucosispora	38	0.04%
Desmonostoc muscorum	38	0.04%
unclassified Cyanobacteria	37	0.03%
unclassified Dactylosporangium	37	0.03%
unclassified Holophagae	37	0.03%
unclassified Blastocatella	37	0.03%
unclassified Aquabacterium	36	0.03%
unclassified Turicibacter	36	0.03%
unclassified Anaerolinea	36	0.03%
unclassified Chelatococcus	36	0.03%
unclassified Luedemannella	36	0.03%
unclassified Acidothermaceae	36	0.03%
unclassified Mucilaginibacter	36	0.03%
Bacillus niacini	36	0.03%
Pseudomonas frederiksbergensis	36	0.03%
unclassified Dehalococcoides	35	0.03%
unclassified Chromatiales	35	0.03%
Pseudomonas viridiflava	35	0.03%
unclassified Desulfosporosinus	35	0.03%
Agromyces iriomotensis	35	0.03%
Turicibacter sanguinis	35	0.03%
unclassified Bacteroidales	35	0.03%
Phenylobacterium muchangponense	35	0.03%
Microvirga aerilata	34	0.03%
unclassified Pseudoruegeria	34	0.03%
Skermanella stibiiresistens	34	0.03%
Pseudoxanthomonas ginsengisoli	34	0.03%
Pedobacter cryoconitis	34	0.03%
Sporosarcina ginsengisoli	34	0.03%
Pedobacter panaciterrae	34	0.03%

Species	Number of reads	Relative abundance
unclassified Terrisporobacter	34	0.03%
unclassified Thermoleophilia	34	0.03%
unclassified Rikenellaceae	34	0.03%
unclassified Bartonella	34	0.03%
Clostridium cellulovorans	34	0.03%
unclassified Phenylobacterium	34	0.03%
Mycobacterium cosmeticum	33	0.03%
unclassified Intrasporangiaceae	33	0.03%
unclassified Pezizales	33	0.03%
Sphingomonas wittichii	33	0.03%
unclassified Cystobacter	33	0.03%
unclassified Frankiaceae	33	0.03%
unclassified Nannocystaceae	33	0.03%
Nocardioides jensenii	33	0.03%
unclassified Proteobacteria	33	0.03%
unclassified Acidimicrobium	33	0.03%
Pseudomonas mandelii	33	0.03%
unclassified Vitis	32	0.03%
unclassified Chlamydiia	32	0.03%
unclassified Lishizhenia	32	0.03%
Phycicola gilvus	32	0.03%
Lysobacter niabensis	32	0.03%
unclassified Microthrixaceae	32	0.03%
unclassified Sphingobacterium	32	0.03%
Kribbella swartbergensis	31	0.03%
unclassified Chthoniobacterales	31	0.03%
Nitrospira cf.	31	0.03%
unclassified Syntrophaceae	31	0.03%
unclassified Actinoplanes	30	0.03%
Kribbella catacumbae	30	0.03%
Streptomyces radiopugnans	30	0.03%
unclassified Rhizocola	30	0.03%
Solitalea koreensis	30	0.03%
unclassified Pelobacteraceae	30	0.03%
unclassified Cytophaga	30	0.03%
unclassified Catellatospora	30	0.03%
unclassified Aquicella	29	0.03%
unclassified Psychrosinus	29	0.03%
Nocardioides koreensis	29	0.03%
Paenibacillus chondroitinus	29	0.03%
Pseudoxanthomonas yeongjuensis	29	0.03%
unclassified Myxococcaceae	29	0.03%
unclassified Dechloromonas	29	0.03%
Streptomyces atratus	29	0.03%
Hyphomicrobium facile	28	0.03%
unclassified Paracoccus	28	0.03%
Desulfosporosinus meridiei	28	0.03%
Pedobacter agri	28	0.03%
unclassified Agrobacterium	27	0.02%
unclassified Verrucomicrobiae	27	0.02%

Species	Number of reads	Relative abundance
Microlunatus aurantiacus	27	0.02%
unclassified Epsilonproteobacteria	27	0.02%
unclassified Rhizobacter	27	0.02%
Agromyces ulmi	26	0.02%
Rhizobacter fulvus	26	0.02%
unclassified Pseudonocardiaceae	26	0.02%
unclassified Spirobacillales	26	0.02%
Bosea thiooxidans	26	0.02%
unclassified Solibacillus	26	0.02%
unclassified Carboxydothermus	26	0.02%
unclassified Caulobacter	26	0.02%
Flavobacterium hibernum	26	0.02%
Microbacterium aurum	26	0.02%
Methylibium petroleiphilum	26	0.02%
unclassified Fimbriimonadaceae	26	0.02%
unclassified Dehalogenimonas	25	0.02%
unclassified Thermomonas	25	0.02%
Pedobacter steynii	25	0.02%
unclassified Chloracidobacteria	25	0.02%
unclassified Stenotrophomonas	25	0.02%
Bacillus firmus	25	0.02%
unclassified Rhodobacteraceae	25	0.02%
unclassified Chthonomonadetes	25	0.02%
unclassified Desulfovibrionaceae	24	0.02%
Nordella oligomobilis	24	0.02%
unclassified Sporocytophaga	24	0.02%
unclassified Kofleriaceae	24	0.02%
Massilia aurea	24	0.02%
unclassified Propionivibrio	24	0.02%
unclassified Piscirickettsiaceae	24	0.02%
unclassified Rubrobacteria	24	0.02%
Rubrivivax gelatinosus	24	0.02%
unclassified Chlorellaceae	24	0.02%
Nitrosovibrio tenuis	24	0.02%
Lysobacter dokdonensis	24	0.02%
unclassified Ectothiorhodospiraceae	24	0.02%
unclassified Pasteuria	23	0.02%
unclassified Malonomonas	23	0.02%
Phyllobacterium myrsinacearum	23	0.02%
unclassified Phycisphaerae	23	0.02%
Psychrobacillus psychrodurans	23	0.02%
Leptothrix ginsengisoli	23	0.02%
unclassified Rhodoligotrophos	23	0.02%
Rummeliibacillus pycnus	23	0.02%
crenarchaeote enrichment	23	0.02%
unclassified Desulfuromonadaceae	23	0.02%
unclassified Hyphomonadaceae	22	0.02%
unclassified Acidisphaera	22	0.02%
unclassified Pedosphaera	22	0.02%
Rhodococcus kunmingensis	22	0.02%

Species	Number of reads	Relative abundance
Mesorhizobium albiziae	22	0.02%
Nocardioides furvisabuli	22	0.02%
unclassified Telluria	22	0.02%
unclassified Chlorellales	22	0.02%
unclassified Dryopteridaceae	22	0.02%
Oryzihumus leptocrescens	22	0.02%
unclassified Actinoallomurus	22	0.02%
unclassified Fimbriimonas	22	0.02%
Clostridium maritimum	22	0.02%
Agromyces cerinus	22	0.02%
unclassified Novosphingobium	22	0.02%
unclassified Pontibacter	21	0.02%
unclassified Desulfobacterales	21	0.02%
unclassified Rhodobacterales	21	0.02%
Pseudomonas umsongensis	21	0.02%
Virgisporangium ochraceum	21	0.02%
unclassified Schlegelella	21	0.02%
unclassified Bradyrhizobiaceae	21	0.02%
unclassified Fluviicola	21	0.02%
unclassified Niabella	21	0.02%
Methylotenera mobilis	21	0.02%
unclassified Plesiocystis	21	0.02%
unclassified Ericaceae	21	0.02%
Thermoactinomyces vulgaris	21	0.02%
Rhodococcus globerulus	21	0.02%
Mycobacterium hodleri	20	0.02%
unclassified Tetracoccus	20	0.02%
Nonomuraea candida	20	0.02%
Curtobacterium plantarum	20	0.02%
Hamadaea tsunoensis	20	0.02%
unclassified Herbaspirillum	20	0.02%
unclassified Terriglobus	20	0.02%
unclassified Solibacteres	20	0.02%
Stenotrophomonas rhizophila	20	0.02%
unclassified Chlorella	20	0.02%
unclassified Dyadobacter	20	0.02%
unclassified Flammeovirgaceae	19	0.02%
unclassified Candidatus Scalindua	19	0.02%
Couchioplanes caeruleus	19	0.02%
Promicromonospora sukumoe	19	0.02%
Catellatospora citrea	19	0.02%
Sphingomonas humi	19	0.02%
Williamsia faeni	19	0.02%
unclassified Aeromicrobium	19	0.02%
cf. Chryseobacterium	19	0.02%
Rhodoferax ferrireducens	19	0.02%
unclassified Crenarchaeota	19	0.02%
Paenibacillus amylolyticus	19	0.02%
unclassified Skermanella	19	0.02%
Sphingomonas asaccharolytica	19	0.02%

Species	Number of reads	Relative abundance
unclassified Dehalococcoidia	19	0.02%
unclassified Kineosporia	19	0.02%
Stenotrophomonas maltophilia	19	0.02%
unclassified Desulfoglaeba	18	0.01%
unclassified Pedosphaerae	18	0.01%
Sporosarcina globispora	18	0.01%
Pedomicrobium australicum	18	0.01%
unclassified Gracilibacteraceae	18	0.01%
unclassified Sphingobium	18	0.01%
Rhodococcus tukisamuensis	18	0.01%
unclassified Salinibacterium	18	0.01%
unclassified Synechococcophycideae	18	0.01%
unclassified Microlunatus	18	0.01%
Flavobacterium frigidarium	18	0.01%
unclassified Gallionellales	18	0.01%
Agromyces fucosus	18	0.01%
Spirilliplanes yamanashiensis	18	0.01%
unclassified Thermoanaerobacteraceae	18	0.01%
Nocardia cummidelens	17	0.01%
unclassified Taibaiella	17	0.01%
unclassified Peptococcaceae	17	0.01%
unclassified Phaeodactylum	17	0.01%
unclassified Pseudoxanthomonas	17	0.01%
Clostridium pasteurianum	17	0.01%
Sphingopyxis alaskensis	17	0.01%
Flectobacillus lacus	17	0.01%
proteobacterium enrichment	17	0.01%
Kribbella albertanoniae	17	0.01%
Nocardioides alpinus	17	0.01%
unclassified Streptosporangiaceae	17	0.01%
unclassified Fimbriimonadales	17	0.01%
unclassified Enhygromyxa	17	0.01%
unclassified Lachnospiraceae	17	0.01%
unclassified Brocadiaceae	17	0.01%
unclassified Xanthomonas	17	0.01%
unclassified Rubrobacteraceae	16	0.01%
unclassified Aridibacter	16	0.01%
unclassified Altererythrobacter	16	0.01%
Dactylosporangium darangshiense	16	0.01%
unclassified Nocardiaceae	16	0.01%
Janthinobacterium lividum	16	0.01%
unclassified Ktedonobacteraceae	16	0.01%
unclassified Rhodobium	16	0.01%
unclassified Flavihumibacter	16	0.01%
unclassified Paucimonas	16	0.01%
Nocardioides caricicola	16	0.01%
unclassified Chloroflexia	15	0.01%
Nonomuraea maheshkhaliensis	15	0.01%
unclassified Defluviicoccus	15	0.01%
unclassified Kurthia	15	0.01%

Species	Number of reads	Relative abundance
unclassified Paludibacter	15	0.01%
unclassified Veillonellaceae	15	0.01%
Pantoea vagans	15	0.01%
unclassified Phyllobacterium	15	0.01%
unclassified Gaiellales	15	0.01%
Microbacterium lacticum	15	0.01%
unclassified Hymenobacter	15	0.01%
unclassified Planococcaceae	15	0.01%
Lysinibacillus boronitolerans	15	0.01%
unclassified Tissierella	15	0.01%
Paenisporosarcina quisquiliarum	15	0.01%
unclassified Ignavibacteria	15	0.01%
unclassified Rhodobiaceae	14	0.01%
unclassified Candidatus Koribacter	14	0.01%
unclassified Ramlibacter	14	0.01%
unclassified Labilitrichaceae	14	0.01%
unclassified Roseiflexaceae	14	0.01%
Nocardioides oleivorans	14	0.01%
Caulobacter henricii	14	0.01%
unclassified Marinicellaceae	14	0.01%
unclassified Blastomonas	14	0.01%
Microbacterium maritypicum	14	0.01%
Mycobacterium murale	14	0.01%
unclassified Rhodoferax	14	0.01%
unclassified Patulibacteraceae	14	0.01%
Flavobacterium psychrophilum	14	0.01%
Aeromicrobium ginsengisoli	14	0.01%
Mycobacterium monacense	14	0.01%
unclassified Tumebacillus	13	0.01%
Kaistia granuli	13	0.01%
unclassified Rhodobacter	13	0.01%
unclassified Ensifer	13	0.01%
unclassified Campylobacterales	13	0.01%
unclassified Polaromonas	13	0.01%
Laceyella sacchari	13	0.01%
unclassified Caloramator	13	0.01%
unclassified Hydrogenophilales	13	0.01%
unclassified Rubrobacter	13	0.01%
unclassified Erythrobacter	13	0.01%
Pantoea agglomerans	13	0.01%
Sanguibacter inulinus	13	0.01%
Pedobacter caeni	13	0.01%
marine actinobacterium	13	0.01%
unclassified Actinomycetaceae	13	0.01%
unclassified Caryophanon	13	0.01%
unclassified Desulfovibrionales	13	0.01%
unclassified Acetivibrio	13	0.01%
Novosphingobium aromaticivorans	13	0.01%
Nocardioides aestuarii	13	0.01%
unclassified Prosthecomicrobium	13	0.01%

Species	Number of reads	Relative abundance
unclassified Stella	13	0.01%
Methylosinus trichosporium	13	0.01%
unclassified Cellulosilyticum	13	0.01%
unclassified Allocatelliglobosispora	13	0.01%
Archangium gephyra	12	0.01%
unclassified Gracilibacter	12	0.01%
Mesorhizobium plurifarium	12	0.01%
unclassified Caldicoprobacter	12	0.01%
Dyadobacter alkalitolerans	12	0.01%
unclassified Archangium	12	0.01%
Bacillus circulans	12	0.01%
Labrys wisconsinensis	12	0.01%
Flavobacterium psychrolimnae	12	0.01%
unclassified Methylocystaceae	12	0.01%
Mesorhizobium chacoense	12	0.01%
unclassified Nitrosovibrio	12	0.01%
unclassified Sanguibacter	12	0.01%
Pseudomonas punonensis	12	0.01%
unclassified Brevundimonas	12	0.01%
unclassified Crocinitomix	12	0.01%
unclassified Nakamurellaceae	12	0.01%
unclassified Phyllobacteriaceae	12	0.01%
Mycobacterium bacteremicum	12	0.01%
Lysobacter ginsengisoli	12	0.01%
unclassified Methylotenera	12	0.01%
unclassified Beijerinckiaceae	12	0.01%
Acetivibrio cellulolyticus	12	0.01%
unclassified Desulforegula	12	0.01%
unclassified Caulobacteraceae	12	0.01%
unclassified Rubritalea	12	0.01%
Streptomyces rubrolavendulae	12	0.01%
unclassified Catenulisporaceae	11	0.01%
unclassified Cellulomonas	11	0.01%
unclassified Oscillatoriophycideae	11	0.01%
unclassified Nostoc	11	0.01%
unclassified Staphylococcus	11	0.01%
Pseudomonas caeni	11	0.01%
Phenylobacterium immobile	11	0.01%
unclassified Humibacillus	11	0.01%
Bacillus murimartini	11	0.01%
unclassified Cyanobacteriaceae	11	0.01%
unclassified Agrococcus	11	0.01%
Kineosporia rhamnosa	11	0.01%
unclassified Alteromonadaceae	11	0.01%
unclassified Thermus	11	0.01%
unclassified Azospirillum	11	0.01%
Cystobacter ferrugineus	11	0.01%
unclassified Phycicoccus	11	0.01%
Sporacetigenium mesophilum	11	0.01%
Kurthia huakuii	11	0.01%

Species	Number of reads	Relative abundance
Pseudorhodoferax aquiterrae	11	0.01%
unclassified Sedimentibacter	11	0.01%
unclassified Chthonomonadaceae	11	0.01%
unclassified Nocardia	11	0.01%
unclassified Gordonibacter	11	0.01%
unclassified Thermoflavimicrobium	11	0.01%
Pseudomonas putida	11	0.01%
unclassified Isosphaeraceae	11	0.01%
Rhodococcus opacus	11	0.01%
unclassified Cryocola	11	0.01%
unclassified Anaeroplasmataceae	11	0.01%
unclassified Pinaceae	11	0.01%
Psychrobacillus psychrotolerans	11	0.01%
unclassified Pseudosporangium	11	0.01%
unclassified Rhodomicrobium	10	0.01%
unclassified Rhodothermi	10	0.01%
unclassified Anoxybacillus	10	0.01%
unclassified Candidatus Microthrix	10	0.01%
unclassified Tissierella_Soehngenia	10	0.01%
Massilia timonae	10	0.01%
Leptothrix mobilis	10	0.01%
Rhodococcus coprophilus	10	0.01%
Dryopteris environmental	10	0.01%
unclassified Opitutae	10	0.01%
unclassified Tepidamorphus	10	0.01%
Bacillus foraminis	10	0.01%
Sphingobacterium anhuiense	10	0.01%
Cryobacterium psychrophilum	10	0.01%
Dietzia lutea	10	0.01%
unclassified Armatimonadetes	10	0.01%
Glycomyces harbinensis	10	0.01%
unclassified Thermoanaerobacter	10	0.01%
Mycobacterium rhodesiae	10	0.01%
Massilia niabensis	10	0.01%
unclassified Elusimicrobia	10	0.01%
unclassified Oxobacter	10	0.01%
Cylindrospermum muscicola	10	0.01%
unclassified Fusobacterium	10	0.01%
unclassified Hydrogenophilaceae	10	0.01%
Clostridium favososporum	10	0.01%
unclassified Acidimicrobiia	10	0.01%
Corallococcus macrosporus	10	0.01%
unclassified Actinomycetospora	10	0.01%
Paenibacillus algorifonticola	10	0.01%
Caulobacter mirabilis	10	0.01%
Sphingomonas oryziterrae	10	0.01%
unclassified Polypodiidae	9	0%
Actinoplanes philippinensis	9	0%
Bacillus gibsonii	9	0%
Kaistia terrae	9	0%

Species	Number of reads	Relative abundance
unclassified Rhodothermales	9	0%
unclassified Paradevosia	9	0%
Mycobacterium marinum	9	0%
unclassified Phycisphaeraceae	9	0%
unclassified Thiorhodovibrio	9	0%
Duganella zoogloeoides	9	0%
unclassified Zhihengliuella	9	0%
Aetherobacter rufus	9	0%
Clostridium sulfidigenes	9	0%
Bacillus drentensis	9	0%
unclassified Psychrobacillus	9	0%
Verrucosispora gifhornensis	9	0%
unclassified Cohnella	9	0%
unclassified Desulfobulbus	9	0%
unclassified Cyanobacterium	9	0%
unclassified Thioalkalivibrio	9	0%
Pseudoduganella violaceinigra	9	0%
unclassified Jishengella	9	0%
Paenisporosarcina macmurdoensis	9	0%
unclassified Rhodocista	9	0%
unclassified Couchioplanes	9	0%
Actinoplanes xinjiangensis	9	0%
Nocardioides ganghwensis	9	0%
unclassified Candidatus Xiphinematobacter	9	0%
unclassified Thiobacteraceae	9	0%
unclassified Eubacteriaceae	9	0%
Mycobacterium sediminis	9	0%
Sporosarcina luteola	9	0%
unclassified Corallococcus	8	0%
unclassified Caulobacterales	8	0%
Paenibacillus terrae	8	0%
Paenibacillus castaneae	8	0%
unclassified Filimonas	8	0%
unclassified Hydrogenedentes	8	0%
Fictibacillus phosphorivorans	8	0%
unclassified Caldimonas	8	0%
Mycobacterium goodii	8	0%
unclassified Erythromicrobium	8	0%
Bacillus selenatarsenatis	8	0%
Mycobacterium tuberculosis	8	0%
unclassified Thermoactinomycetaceae	8	0%
Clostridium bifermentans	8	0%
unclassified Lactobacillales	8	0%
unclassified Bacteroidaceae	8	0%
Paenibacillus pectinilyticus	8	0%
Nostoc ellipsosporum	8	0%
unclassified Leucobacter	8	0%
Ammoniphilus oxalaticus	8	0%
unclassified Asteroleplasma	8	0%
Nitrospira marina	8	0%

Species	Number of reads	Relative abundance
unclassified Anaeroplasmatales	8	0%
unclassified Candidatus Chloracidobacterium	8	0%
unclassified Mollicutes	8	0%
Actinoallomurus iriomotensis	8	0%
Dyadobacter hamtensis	8	0%
unclassified Rhizomicrobium	8	0%
Nostoc punctiforme	8	0%
Rhodococcus maanshanensis	8	0%
unclassified Brocadiales	8	0%
Actinophytocola xinjiangensis	8	0%
Corynebacterium glutamicum	8	0%
unclassified Litorilinea	8	0%
unclassified Kineosporiaceae	8	0%
unclassified Ochrobactrum	8	0%
unclassified Koribacteraceae	8	0%
unclassified	8	0%
unclassified Isosphaera	8	0%
Brevibacterium flavum	8	0%
actinobacterium SCGC	7	0%
Microbacterium foliorum	7	0%
unclassified Thiotrichales	7	0%
unclassified Chlorobi	7	0%
unclassified Promicromonospora	7	0%
Clostridium vincentii	7	0%
unclassified Oryzihumus	7	0%
unclassified Shimazuella	7	0%
unclassified Cystobacterineae	7	0%
Comamonas aquatica	7	0%
Agromyces neolithicus	7	0%
unclassified Actinocorallia	7	0%
Pseudomonas lini	7	0%
Bosea lupini	7	0%
unclassified Stigmatella	7	0%
Devosia terrae	7	0%
unclassified Gallionella	7	0%
Allocatelliglobosispora scoriae	7	0%
Pedobacter wanjuense	7	0%
unclassified Chthonomonadales	7	0%
unclassified Planococcus	7	0%
unclassified Streptosporangium	7	0%
unclassified Thermomicrobium	7	0%
unclassified Thermomonosporaceae	7	0%
unclassified Beijerinckia	7	0%
Jatrophihabitans endophyticus	7	0%
Candidatus Nitrospira	7	0%
Stenotrophomonas acidaminiphila	7	0%
Microbacterium thalassium	7	0%
Lysinibacillus sphaericus	7	0%
Flavobacterium columnare	7	0%
unclassified Armatimonadaceae	7	0%

Species	Number of reads	Relative abundance
Hyphomicrobium vulgare	7	0%
unclassified Krasilnikovia	7	0%
Cryocola poae	7	0%
Rhodococcus fascians	7	0%
Mycobacterium szulgai	7	0%
Terrimonas lutea	7	0%
Ancylobacter dichloromethanicus	7	0%
Beijerinckia indica	7	0%
unclassified Tetrasphaera	7	0%
Sphingobacterium faecium	7	0%
Clostridium gasigenes	7	0%
unclassified Herpetosiphon	7	0%
unclassified Actinomadura	7	0%
Nocardioides terrigena	7	0%
Brevundimonas lenta	7	0%
unclassified Haslea	7	0%
unclassified Alicyclobacillaceae	7	0%
unclassified Clavibacter	7	0%
Clavibacter michiganensis	7	0%
unclassified Aetherobacter	7	0%
Paenibacillus catalpae	7	0%
Brevibacillus thermoruber	6	0%
Aminobacter aminovorans	6	0%
Paenibacillus antarcticus	6	0%
unclassified Microbispora	6	0%
unclassified Porphyromonadaceae	6	0%
unclassified Kofleria	6	0%
Paenibacillus daejeonensis	6	0%
Williamsia muralis	6	0%
unclassified Cerasicoccales	6	0%
Pedobacter insulae	6	0%
Arthrobacter defluvii	6	0%
unclassified Sphingosinicella	6	0%
unclassified Smaragdicoccus	6	0%
Clostridium hungatei	6	0%
Actinoplanes abujensis	6	0%
unclassified Scenedesmaceae	6	0%
Halomonas phoceae	6	0%
Amaricoccus tamworthensis	6	0%
unclassified Firmicutes	6	0%
unclassified Rummeliibacillus	6	0%
Acetobacterium bakii	6	0%
Smaragdicoccus niigatensis	6	0%
unclassified Duganella	6	0%
Devosia geojensis	6	0%
unclassified Peptoclostridium	6	0%
unclassified mitochondria	6	0%
Actinoplanes friuliensis	6	0%
delta proteobacterium	6	0%
Bacillus mycoides	6	0%

Species	Number of reads	Relative abundance
Bacillus asahii	6	0%
Mycobacterium smegmatis	6	0%
unclassified Epulopiscium	6	0%
unclassified Lentzea	6	0%
Pantoea brenneri	6	0%
Acidovorax soli	6	0%
unclassified Ktedonobacterales	6	0%
Brevundimonas denitrificans	6	0%
unclassified Bacteroidia	6	0%
Cohnella lupini	6	0%
unclassified Ktedonobacter	6	0%
unclassified Desulfocapsa	6	0%
Agrobacterium sullae	6	0%
Pseudomonas syringae	6	0%
unclassified Noviherbaspirillum	6	0%
unclassified Coprococcus	6	0%
unclassified Parvarchaea	6	0%
Nocardia exalbida	6	0%
Frigoribacterium faeni	6	0%
unclassified Erythrobacteraceae	6	0%
unclassified Lacibacter	6	0%
Microbacterium deminutum	6	0%
Actinomadura vinacea	6	0%
Mesorhizobium septentrionale	6	0%
Pseudomonas rhizosphaerae	6	0%
unclassified Desulfuromonas	6	0%
unclassified Enterobacteriales	6	0%
Sphingomonas echinoides	6	0%
Bacillus pumilus	5	0%
unclassified Oligoflexaceae	5	0%
unclassified Rhodovastum	5	0%
Mycobacterium pyrenivorans	5	0%
unclassified Pelosinus	5	0%
Arthrobacter psychrolactophilus	5	0%
Streptomyces mashuensis	5	0%
unclassified Kaistia	5	0%
Mucilaginibacter calamicampi	5	0%
Mycobacterium celatum	5	0%
Mesorhizobium mediterraneum	5	0%
Polaromonas naphthalenivorans	5	0%
unclassified Pseudoclavibacter	5	0%
Pedobacter alluvionis	5	0%
Actinocorallia longicatena	5	0%
Nitrospira moscoviensis	5	0%
unclassified Desulfomonile	5	0%
unclassified Thermoactinomyces	5	0%
Clostridium tunisiense	5	0%
unclassified Uliginosibacterium	5	0%
Pseudonocardia xinjiangensis	5	0%
Pseudosporangium ferrugineum	5	0%

Species	Number of reads	Relative abundance
unclassified Balneimonas	5	0%
Pseudomonas fluorescens	5	0%
unclassified Ignavibacteriales	5	0%
Massilia brevitalea	5	0%
unclassified Caenimonas	5	0%
unclassified Anaerosporobacter	5	0%
Aridibacter famidurans	5	0%
Clostridium populeti	5	0%
unclassified Roseimicrobium	5	0%
unclassified Phycicola	5	0%
Shinella granuli	5	0%
unclassified Angustibacter	5	0%
Acinetobacter lwoffii	5	0%
unclassified Leadbetterella	5	0%
Nocardioides kribbensis	5	0%
unclassified Bacteroides	5	0%
unclassified IIb	5	0%
unclassified Thermococcales	5	0%
Mesorhizobium ciceri	5	0%
Pseudaminobacter defluvii	5	0%
unclassified Salibacter	5	0%
unclassified Jatrophihabitans	5	0%
unclassified Vasilyevaea	5	0%
unclassified Caldicoprobacteraceae	5	0%
unclassified Dyella	5	0%
Clostridium aurantibutyricum	5	0%
unclassified Thermodesulfovibrionaceae	5	0%
Flavisolibacter ginsengiterrae	5	0%
unclassified Curtobacterium	5	0%
unclassified Paucibacter	5	0%
Mycobacterium barrassiae	5	0%
unclassified Sandaracinaceae	5	0%
Rhizobium daejeonense	5	0%
unclassified Cryobacterium	5	0%
unclassified Ruminococcus	5	0%
Phyllobacterium ifriqiyense	5	0%
unclassified Prolixibacteraceae	5	0%
Pedobacter duraquae	5	0%
unclassified Halanaerobiales	5	0%
unclassified Methylophilales	5	0%
Kribbella flavida	5	0%
Phaeodactylum tricornutum	5	0%
unclassified Rhodovulum	5	0%
Polymorphospora rubra	5	0%
unclassified Candidatus Protochlamydia	5	0%
Terrabacter terrae	5	0%
Pedobacter boryungensis	4	0%
unclassified Syntrophomonadaceae	4	0%
unclassified Peptostreptococcaceae	4	0%
Bosea massiliensis	4	0%

Species	Number of reads	Relative abundance
unclassified Oxalobacter	4	0%
unclassified Candidatus Brocadiales	4	0%
unclassified Entotheonellales	4	0%
unclassified Fodinicola	4	0%
unclassified Williamsia	4	0%
Chitinophaga soli	4	0%
unclassified Oceanobacillus	4	0%
Devosia submarina	4	0%
unclassified Nitrosomonas	4	0%
Umezawaea tangerina	4	0%
Kribbella antibiotica	4	0%
Polaromonas ginsengisoli	4	0%
Rhizobium cellulosilyticum	4	0%
Corynebacterium variabile	4	0%
Acinetobacter johnsonii	4	0%
unclassified Negativicutes	4	0%
unclassified Polymorphospora	4	0%
Pseudoxanthomonas mexicana	4	0%
Streptomyces tauricus	4	0%
Thermoactinomyces intermedius	4	0%
Streptomyces tempisquensis	4	0%
Catenuloplanes japonicus	4	0%
Streptomyces aureofaciens	4	0%
Flavobacterium resistens	4	0%
Cryocola antiquus	4	0%
Naxibacter indica	4	0%
unclassified Gallionellaceae	4	0%
Stenotrophomonas retroflexus	4	0%
Geobacter psychrophilus	4	0%
unclassified Oceanospirillales	4	0%
unclassified Nostocales	4	0%
unclassified Terrabacter	4	0%
Mesorhizobium loti	4	0%
unclassified Xanthobacteraceae	4	0%
unclassified Bacteriovorax	4	0%
Flavobacterium glaciei	4	0%
Micromonospora purpureochromogenes	4	0%
Nocardioides albus	4	0%
unclassified Hyalangium	4	0%
unclassified Solitalea	4	0%
unclassified Limnohabitans	4	0%
Empedobacter brevis	4	0%
unclassified Alkaliphilus	4	0%
unclassified Candidatus Alysiosphaera	4	0%
Luteolibacter luojiensis	4	0%
Nocardioides caeni	4	0%
unclassified Haloplasmataceae	4	0%
unclassified Candidatus Entotheonella	4	0%
unclassified Jahnella	4	0%
Aeromicrobium erythreum	4	0%

Species	Number of reads	Relative abundance
unclassified Virgisporangium	4	0%
Paenibacillus sepulcri	4	0%
unclassified Methyloceanibacter	4	0%
unclassified Glycomyces	4	0%
unclassified Methylocella	4	0%
Micromonospora lupini	4	0%
Pseudomonas koreensis	4	0%
Cellvibrio ostraviensis	4	0%
unclassified Desmonostoc	4	0%
Kineosporia mesophila	4	0%
Lysobacter cookii	4	0%
Dyadobacter psychrophilus	4	0%
unclassified Cryptosporangiaceae	4	0%
Novosphingobium barchaimii	4	0%
unclassified Bogoriellaceae	4	0%
unclassified Chroococcales	4	0%
Chryseobacterium indoltheticum	4	0%
Actinomadura scrupuli	4	0%
Novosphingobium resinovorum	4	0%
unclassified Ureibacillus	4	0%
Cystobacter violaceus	4	0%
Luteimonas terricola	4	0%
Tumebacillus ginsengisoli	4	0%
unclassified Pantoea	4	0%
Geobacillus thermoglucosidasius	4	0%
unclassified Roseburia	4	0%
Actinoplanes liguriensis	4	0%
unclassified Paenirhodobacter	4	0%
unclassified Rickettsiaceae	4	0%
unclassified Alsobacter	4	0%
unclassified Euzebya	4	0%
Chryseolinea serpens	4	0%
Roseomonas lacus	4	0%
Microbispora rosea	4	0%
unclassified Methylacidiphilales	4	0%
Rhizobium nepotum	4	0%
Phenylobacterium haematophilum	4	0%
Clostridium beijerinckii	4	0%
unclassified Oerskovia	4	0%
Sphingobacteriaceae str.	4	0%
Agrobacterium rhizogenes	4	0%
Nostoc commune	4	0%
unclassified Glycomycetaceae	4	0%
Methylosinus sporium	4	0%
Aquabacterium citratiphilum	4	0%
Pedobacter bauzanensis	3	0%
unclassified Geodermatophilus	3	0%
Clostridium longisporum	3	0%
unclassified Anabaena	3	0%
Bacillus thermolactis	3	0%

Species	Number of reads	Relative abundance
Streptomyces kagoshimanus	3	0%
Pedobacter jejuensis	3	0%
unclassified Planifilum	3	0%
unclassified Sandaracinus	3	0%
Nocardia tenerifensis	3	0%
unclassified Laceyella	3	0%
unclassified Fusobacteriaceae	3	0%
unclassified Undibacterium	3	0%
unclassified Methylobacter	3	0%
Solirubrobacter soli	3	0%
unclassified Citreicella	3	0%
Amaricoccus macauensis	3	0%
unclassified Maricaulis	3	0%
Sphingobacterium mizutaii	3	0%
Clostridium akagii	3	0%
unclassified Thiobacterales	3	0%
Planifilum yunnanense	3	0%
Streptomyces lilacinus	3	0%
unclassified Melioribacteraceae	3	0%
Paenibacillus alginolyticus	3	0%
Rathayibacter caricis	3	0%
Massilia dura	3	0%
unclassified Pelomonas	3	0%
Bacillus licheniformis	3	0%
Clostridium papyrosolvens	3	0%
Intrasporangium mesophilum	3	0%
Sporosarcina thermotolerans	3	0%
Pedobacter nyackensis	3	0%
unclassified Chlorophyceae	3	0%
Gaiella occulta	3	0%
Mycobacterium intracellulare	3	0%
Arenimonas malthae	3	0%
unclassified Chlamydomonadales	3	0%
unclassified Fluoribacter	3	0%
Arthrobacter rhombi	3	0%
unclassified Comamonas	3	0%
Sanguibacter antarcticus	3	0%
unclassified Lactococcus	3	0%
Dyadobacter fermentans	3	0%
unclassified Chromatiaceae	3	0%
unclassified Thermomicrobiaceae	3	0%
unclassified Spirilliplanes	3	0%
unclassified Halomonas	3	0%
Clostridium algidicarnis	3	0%
Catenuloplanes castaneus	3	0%
unclassified Oscillatoriales	3	0%
Dactylosporangium siamense	3	0%
unclassified Ornithinibacillus	3	0%
unclassified Lysinimicrobium	3	0%
Pedobacter borealis	3	0%

Species	Number of reads	Relative abundance
Luteolibacter yonseiensis	3	0%
Alicyclobacillus disulfidooxidans	3	0%
Corynebacterium xerosis	3	0%
unclassified Arthrospira	3	0%
unclassified Dermatophilaceae	3	0%
Agromyces atrinae	3	0%
Promicromonospora aerolata	3	0%
Cystobacter fuscus	3	0%
unclassified Gemmobacter	3	0%
Actinopolymorpha pittospori	3	0%
unclassified Anaerovorax	3	0%
Phenylobacterium falsum	3	0%
Angustibacter luteus	3	0%
unclassified Nitriliruptoridae	3	0%
unclassified Pseudoduganella	3	0%
Phenylobacterium mobile	3	0%
Prevotella copri	3	0%
Massilia suwonensis	3	0%
Nocardia carnea	3	0%
unclassified Rudaea	3	0%
unclassified Zoogloea	3	0%
Brevundimonas diminuta	3	0%
Blastococcus jejuensis	3	0%
Lysobacter spongiicola	3	0%
Sporosarcina saromensis	3	0%
Paenibacillus turicensis	3	0%
Micromonospora peucetia	3	0%
unclassified Methylobacillus	3	0%
Streptococcus alactolyticus	3	0%
Herminiimonas fonticola	3	0%
unclassified Prevotellaceae	3	0%
Devosia limi	3	0%
Mucilaginibacter rigui	3	0%
Nocardioides hankookensis	3	0%
Mycobacterium litorale	3	0%
Flavobacterium johnsoniae	3	0%
Devosia soli	3	0%
Paenibacillus borealis	3	0%
Neorhizobium galegae	3	0%
Aeromicrobium kwangyangensis	3	0%
Rhizobium leguminosarum	3	0%
unclassified Acholeplasmataceae	3	0%
unclassified Ktedonobacteria	3	0%
Microvirga zambiensis	3	0%
Micromonospora hermanusense	3	0%
unclassified Schlesneria	3	0%
Arthrospira fusiformis	3	0%
Mycobacterium confluentis	3	0%
unclassified Vogesella	3	0%
unclassified Actinophytocola	3	0%

Species	Number of reads	Relative abundance
Microbacterium barkeri	3	0%
unclassified Catelliglobosispora	3	0%
Polaromonas aquatica	3	0%
Physcomitrella patens	3	0%
unclassified Janibacter	3	0%
unclassified Nitrosococcus	3	0%
unclassified Falsirhodobacter	3	0%
Paenibacillus sacheonensis	3	0%
unclassified Acidithiobacillales	3	0%
Clostridium fimetarium	3	0%
Mesorhizobium australicum	3	0%
unclassified Pinidae	3	0%
Clavisporangium rectum	3	0%
Micromonospora coriariae	3	0%
Ureibacillus thermosphaericus	3	0%
unclassified Selenomonadales	3	0%
actinomycete enrichment	3	0%
Mycobacterium fortuitum	3	0%
unclassified Shewanellaceae	3	0%
Lysobacter niastensis	3	0%
Microbispora corallina	3	0%
unclassified Synechococcales	3	0%
unclassified Actinospica	3	0%
unclassified Starria	2	0%
unclassified Geminicoccus	2	0%
Leucobacter komagatae	2	0%
unclassified Methylophilus	2	0%
Sphingobium aromaticiconvertens	2	0%
unclassified Reichenowia	2	0%
unclassified Shinella	2	0%
Paenibacillus koleovorans	2	0%
Ruminococcus flavefaciens	2	0%
unclassified Methylococcales	2	0%
Micromonospora chaiyaphumensis	2	0%
Achromobacter spanius	2	0%
Dyadobacter soli	2	0%
unclassified Coriobacteriales	2	0%
Paenibacillus agaridevorans	2	0%
unclassified Labilithrix	2	0%
unclassified Corynebacterium	2	0%
unclassified Moraxellaceae	2	0%
Mycobacterium insubricum	2	0%
unclassified Actinotalea	2	0%
Actinomycetospora straminea	2	0%
unclassified Umezawaea	2	0%
Cellulomonas uda	2	0%
Nodularia sphaerocarpa	2	0%
Fluviicola taffensis	2	0%
unclassified Nostocoida type II	2	0%
unclassified Fusobacteriia	2	0%

Species	Number of reads	Relative abundance
Gemmobacter changlensis	2	0%
unclassified Phaselicystis	2	0%
unclassified Nostocaceae	2	0%
Reyranella soli	2	0%
Clostridium frigidicarnis	2	0%
unclassified Nodularia	2	0%
Nannocystis pusilla	2	0%
Flavobacterium rivuli	2	0%
Sphingomonas xinjiangensis	2	0%
unclassified Aminobacter	2	0%
Nocardioidaceae str.	2	0%
Flavobacterium limnosediminis	2	0%
unclassified Oscillochloris	2	0%
Clostridium chartatabidum	2	0%
unclassified Enterobacteriaceae	2	0%
unclassified Methylophilaceae	2	0%
Luteimonas composti	2	0%
Acidovorax radicis	2	0%
unclassified Paenisporosarcina	2	0%
Paenibacillus telluris	2	0%
Pseudomonas moorei	2	0%
Bacillus cecembensis	2	0%
Paenibacillus cookii	2	0%
unclassified Elusimicrobium	2	0%
Stenotrophomonas koreensis	2	0%
Chryseobacterium piscium	2	0%
unclassified Faecalibacterium	2	0%
unclassified Ornatilinea	2	0%
unclassified Armatimonadia	2	0%
unclassified Haloplasmatales	2	0%
Pedobacter luteus	2	0%
Methylobacterium mesophilicum	2	0%
Brevundimonas kwangchunensis	2	0%
unclassified Sediminibacterium	2	0%
unclassified Luteimicrobium	2	0%
unclassified Pileolariaceae	2	0%
Hydrogenophaga palleronii	2	0%
unclassified Chloroplast	2	0%
unclassified Propionibacteriaceae	2	0%
unclassified Virgibacillus	2	0%
unclassified Geoalkalibacter	2	0%
Tumebacillus permanentifrigoris	2	0%
Clostridium magnum	2	0%
unclassified Cerasicoccaceae	2	0%
Gordonia caeni	2	0%
Paenibacillus polymyxa	2	0%
unclassified Dietzia	2	0%
Micromonospora pisi	2	0%
unclassified Erwinia	2	0%
Brevundimonas subvibrioides	2	0%

Species	Number of reads	Relative abundance
Lactobacillus sharpeae	2	0%
unclassified Hylemonella	2	0%
unclassified Spirochaetaceae	2	0%
unclassified Gloeobacterophycideae	2	0%
Vasilyevaea enhydra	2	0%
Nocardioides pyridinolyticus	2	0%
Bartonella elizabethae	2	0%
unclassified Syntrophomonas	2	0%
Bacillus humi	2	0%
Mycobacterium mucogenicum	2	0%
unclassified Oscillochloridaceae	2	0%
unclassified Kiloniellales	2	0%
Actinokineospora diospyrosa	2	0%
unclassified Pelotomaculum	2	0%
Clostridium botulinum	2	0%
unclassified Mycoplasmataceae	2	0%
unclassified Ammoniphilus	2	0%
unclassified Filobacillus	2	0%
Agromyces salentinus	2	0%
Lactobacillus similis	2	0%
unclassified Chroococcidiopsis	2	0%
unclassified Ruminiclostridium	2	0%
unclassified Physcomitrella	2	0%
Brevundimonas nasdae	2	0%
Pedobacter heparinus	2	0%
unclassified Ancylobacter	2	0%
Micropruina glycogenica	2	0%
Chryseobacterium aquaticum	2	0%
Kribbella sandramycini	2	0%
Clostridium saccharobutylicum	2	0%
unclassified Synechococcaceae	2	0%
Planctomycete enrichment	2	0%
Acinetobacter bouvetii	2	0%
Nocardia takedensis	2	0%
unclassified Dehalobacterium	2	0%
Sarcina ventriculi	2	0%
Dyadobacter beijingensis	2	0%
unclassified Curvibacter	2	0%
unclassified Eubacterium	2	0%
unclassified Blastopirellula	2	0%
unclassified Methylococcaceae	2	0%
Kroppenstedtia guangzhouensis	2	0%
Cellvibrio fibrivorans	2	0%
unclassified Methanosarcina	2	0%
unclassified Plantactinospora	2	0%
Symbiobacterium thermophilum	2	0%
Taibaiella koreensis	2	0%
Aquabacterium commune	2	0%
unclassified Bryobacter	2	0%
unclassified Intrasporangium	2	0%

Species	Number of reads	Relative abundance
unclassified Jiangella	2	0%
Tahibacter aquaticus	2	0%
Streptomyces ahygroscopicus	2	0%
unclassified Rathayibacter	2	0%
Agromyces terreus	2	0%
Geobacillus thermantarcticus	2	0%
unclassified Thermaerobacter	2	0%
Hyphomicrobium aestuarii	2	0%
unclassified Azospira	2	0%
unclassified Algoriphagus	2	0%
unclassified Aquimonas	2	0%
unclassified Vitellibacter	2	0%
unclassified Moheibacter	2	0%
Sphingopyxis baekryungensis	2	0%
unclassified Candidatus Brocadia	2	0%
unclassified Telmatobacter	2	0%
unclassified Fictibacillus	2	0%
Hyalangium minutum	2	0%
Arcobacter cryaerophilus	2	0%
unclassified Acidipila	2	0%
unclassified Ardenscatenaceae	2	0%
Verrucomicrobium spinosum	2	0%
Rhodobacter ovatus	2	0%
Microbacterium yannicii	2	0%
Paenibacillus rigui	2	0%
unclassified Piscinibacter	2	0%
Mucilaginibacter gynuensis	2	0%
unclassified Fibrobacterales	2	0%
unclassified Tahibacter	2	0%
unclassified Hamadaea	2	0%
Microlunatus phosphovorus	2	0%
Polaromonas vacuolata	2	0%
unclassified Fimbriimonadia	2	0%
Propionibacterium acnes	2	0%
unclassified Candidatus Phytoplasma	2	0%
unclassified Nitrososphaeraceae	2	0%
Pullulanibacillus naganoensis	2	0%
Mycobacterium chubuense	2	0%
Paradevosia shaoguanensis	2	0%
unclassified Propionicimonas	2	0%
Nocardioides exalbidus	2	0%
Shewanella algae	2	0%
unclassified Chloroidium	2	0%
Singulisphaera acidiphila	2	0%
Curtobacterium flaccumfaciens	2	0%
unclassified Chryseobacterium	2	0%
Enterococcus aquimarinus	2	0%
Acidovorax avenae	2	0%
Plantactinospora endophytica	2	0%
unclassified Elusimicrobiales	2	0%

Species	Number of reads	Relative abundance
unclassified Halanaerobiaceae	2	0%
unclassified Patulibacter	2	0%
Rhizobium smilacinae	2	0%
Rahnella aquatilis	2	0%
Arthrobacter crystallopoietes	2	0%
Nocardia jinanensis	2	0%
Pedobacter terrae	2	0%
unclassified Longispora	2	0%
Devosia chinhatensis	2	0%
Caenimonas terrae	2	0%
Massilia niastensis	2	0%
Micromonospora mirobrigensis	2	0%
denitrifying Fe-oxidizing	2	0%
Paenochrobactrum gallinarii	2	0%
unclassified Bacteroidetes	2	0%
Serratia proteamaculans	2	0%
Sorangium cellulosum	2	0%
unclassified Haliea	2	0%
Flavobacterium segetis	2	0%
Cytophaga hutchinsonii	2	0%
unclassified Verrucomicrobia	2	0%
Symbiobacterium turbinis	2	0%
unclassified Segetibacter	2	0%
unclassified Blautia	2	0%
Pedobacter daejeonensis	2	0%
Ilumatobacter fluminis	2	0%
Streptomyces olivoverticillatus	2	0%
Pseudoxanthomonas spadix	2	0%
unclassified Parasporobacterium	2	0%
unclassified Desulfobulbaceae	2	0%
unclassified Leptospiraceae	2	0%
Lysobacter concretionis	2	0%
Pseudomonas stutzeri	2	0%
unclassified Tuberaceae	2	0%
Telluria mixta	2	0%
unclassified Oxalicibacterium	2	0%
Parapusillimonas granuli	2	0%
unclassified Alkalibaculum	2	0%
Microlunatus panaciterrae	2	0%
unclassified Erysipelotrichia	2	0%
unclassified Symbiobacteriaceae	2	0%
Luteimonas aestuarii	2	0%
Cylindrospermum stagnale	2	0%
Citricoccus alkalitolerans	2	0%
Advenella faeciporci	2	0%
Aeromicrobium panaciterrae	2	0%
unclassified Marinilabiliaceae	2	0%
unclassified Flectobacillus	2	0%
Dolichospermum lemmermannii	2	0%
Empedobacter falsenii	2	0%

Species	Number of reads	Relative abundance
Streptomyces albus	2	0%
unclassified Eoetvoesia	2	0%
unclassified Schumannella	2	0%
Streptomyces carnosus	1	0%
Nocardia ignorata	1	0%
unclassified Anaerophaga	1	0%
Flavobacterium subsaxonicum	1	0%
unclassified Chromulinales	1	0%
Nocardia araoensis	1	0%
unclassified Rudaibacter	1	0%
Luteimonas mephitis	1	0%
Nocardioides fonticola	1	0%
unclassified Xiangella	1	0%
Phaeospirillum fulvum	1	0%
Devosia subaequoris	1	0%
unclassified Propioniferax	1	0%
Massilia jejuensis	1	0%
unclassified Elusimicrobiaceae	1	0%
Ferruginibacter lapsinanis	1	0%
Kribbella alba	1	0%
Bacillus polygoni	1	0%
unclassified Syntrophobacter	1	0%
Aureimonas phyllosphaerae	1	0%
Aeromicrobium flavum	1	0%
Nocardioides marinisabuli	1	0%
Streptomyces sioyaensis	1	0%
Cohnella yongneupensis	1	0%
Bacillus altitudinis	1	0%
Algoriphagus winogradskyi	1	0%
unclassified Anaerococcus	1	0%
Paenibacillus prosopidis	1	0%
Ancylobacter abiegnus	1	0%
Paenibacillus macerans	1	0%
unclassified Moorella	1	0%
unclassified Spirosoma	1	0%
unclassified Herminiimonas	1	0%
Weissella hellenica	1	0%
Agrococcus jenensis	1	0%
unclassified Euglenales	1	0%
Actinocorallia libanotica	1	0%
unclassified Neorhizobium	1	0%
unclassified Microcella	1	0%
Clostridium aciditolerans	1	0%
Mesorhizobium huakuii	1	0%
Arthrobacter oxydans	1	0%
unclassified Nocardiopsis	1	0%
Flavobacterium swingsii	1	0%
Blastococcus saxobsidens	1	0%
unclassified Methylorosula	1	0%
Streptomyces thermovulgaris	1	0%

Species	Number of reads	Relative abundance
Hyphomicrobium denitrificans	1	0%
Clostridium mangenotii	1	0%
unclassified Pseudaminobacter	1	0%
Pseudomonas helmanticensis	1	0%
unclassified Kineococcus	1	0%
Pedobacter kyungheensis	1	0%
unclassified Exiguobacterium	1	0%
Azotobacter beijerinckii	1	0%
Pelomonas soli	1	0%
Flavobacterium terrigena	1	0%
unclassified Rivibacter	1	0%
Pelomonas puraquae	1	0%
Microbacterium oxydans	1	0%
Anoxybacillus toebii	1	0%
Vampirovibrio chlorellavorus	1	0%
unclassified Roseococcus	1	0%
Eubacterium eligens	1	0%
Mesorhizobium sangaii	1	0%
unclassified Actinokineospora	1	0%
Actinomycetales str.	1	0%
unclassified Prevotella	1	0%
Pseudonocardia kunmingensis	1	0%
Streptomyces pulveraceus	1	0%
Chryseobacterium haifense	1	0%
unclassified Criblamydiaceae	1	0%
Arenimonas daejeonensis	1	0%
unclassified Ustilaginomycetes	1	0%
unclassified Methylocapsa	1	0%
unclassified Sulfurospirillum	1	0%
unclassified Turicibacteraceae	1	0%
unclassified Spiroplasma	1	0%
unclassified Lutibacterium	1	0%
Pseudomonas oryzihabitans	1	0%
Acinetobacter haemolyticus	1	0%
unclassified Paracraurococcus	1	0%
unclassified Proteiniborus	1	0%
Paenibacillus taichungensis	1	0%
unclassified Aquificae	1	0%
Pelosinus fermentans	1	0%
Clostridium sporogenes	1	0%
Massilia namucuonensis	1	0%
Curtobacterium herbarum	1	0%
Bacillus marisflavi	1	0%
Mycobacterium sphagni	1	0%
unclassified Dermacoccaceae	1	0%
Ochrobactrum pseudogrignonense	1	0%
Bacillus pocheonensis	1	0%
Bacillus farraginis	1	0%
Methylobacterium adhaesivum	1	0%
Microbacterium profundi	1	0%

Species	Number of reads	Relative abundance
unclassified Perlucidibaca	1	0%
Mycobacterium llatzerense	1	0%
unclassified Oligoflexus	1	0%
Cohnella fontinalis	1	0%
unclassified Sporolactobacillaceae	1	0%
Bacillus isronensis	1	0%
Flavisolibacter ginsengisoli	1	0%
Clostridium sartagoforme	1	0%
Nocardioides daejeonensis	1	0%
unclassified Bifidobacteriales	1	0%
unclassified Williamsiaceae	1	0%
Nostoc verrucosum	1	0%
Nocardioides hwasunensis	1	0%
Bacillus kokeshiiformis	1	0%
low G+C	1	0%
Psychrosinus fermentans	1	0%
unclassified Leifsonia	1	0%
Catellatospora methionotrophica	1	0%
Lysobacter gummosus	1	0%
Thermomonospora chromogena	1	0%
Methanosarcina mazei	1	0%
Phycicoccus dokdonensis	1	0%
unclassified Sinorhizobium	1	0%
Solitalea canadensis	1	0%
Ramlibacter henchirensis	1	0%
unclassified Sphaeropleales	1	0%
unclassified Sporolactobacillus	1	0%
Sedimentibacter hongkongensis	1	0%
Mucilaginibacter gracilis	1	0%
Paenibacillus taohuashanense	1	0%
Aquaspirillum arcticum	1	0%
Erwinia billingiae	1	0%
unclassified Ignatzschineria	1	0%
unclassified Olsenella	1	0%
Geobacillus vulcani	1	0%
unclassified Thermincola	1	0%
unclassified Raoultella	1	0%
unclassified Camelimonas	1	0%
unclassified Asticcacaulis	1	0%
Rhodococcus erythropolis	1	0%
Lactobacillus coryniformis	1	0%
Sphingomonas azotofornans	1	0%
unclassified Isoptericola	1	0%
Demequina oxidasica	1	0%
unclassified Anaerobacillus	1	0%
unclassified Armatimonadales	1	0%
unclassified Astrosporangium	1	0%
Paenibacillus graminis	1	0%
unclassified Yonghaparkia	1	0%
Tepidimicrobium xylanilyticum	1	0%

Species	Number of reads	Relative abundance
unclassified Procabacteriales	1	0%
Streptomyces sodiiphilus	1	0%
unclassified Azorhizobium	1	0%
unclassified Thermales	1	0%
Pseudomonas azotoformans	1	0%
unclassified Parvibaculum	1	0%
Rosenbergiella nectarea	1	0%
Paenibacillus terrigena	1	0%
unclassified Alteromonas	1	0%
unclassified Pseudorhodoferax	1	0%
unclassified Stramenopiles	1	0%
Serratia liquefaciens	1	0%
Nitrosospira briensis	1	0%
Luteolibacter algae	1	0%
Sphingomonas changbaiensis	1	0%
unclassified Actinosynnemataceae	1	0%
Streptomyces niger	1	0%
unclassified Geobacillus	1	0%
Lysobacter antibioticus	1	0%
unclassified Azoarcus	1	0%
unclassified Chishuiella	1	0%
Aequorivita capsosiphonis	1	0%
unclassified Bacillariophyceae	1	0%
Paenibacillus ginsengihumi	1	0%
Bacillus safensis	1	0%
unclassified Rarobacter	1	0%
unclassified Phormidiaceae	1	0%
Inquilinus limosus	1	0%
Chitinophaga arvensicola	1	0%
unclassified Kyrpidia	1	0%
unclassified Demequina	1	0%
Nocardiopsis dassonvillei	1	0%
Cupriavidus respiraculi	1	0%
Phyllobacterium trifolii	1	0%
Pigmentiphaga litoralis	1	0%
Leucobacter salsicius	1	0%
Corallococcus exiguus	1	0%
Thermopolyspora flexuosa	1	0%
Chlamydomonas applanata	1	0%
Blastomonas natatoria	1	0%
unclassified Peptostreptococcus	1	0%
Rufibacter tibetensis	1	0%
unclassified Sulfobacillaceae	1	0%
Tabrizicola aquatica	1	0%
Nocardioides lianchengensis	1	0%
Nocardia speluncae	1	0%
Cellvibrio mixtus	1	0%
Clostridium putrefaciens	1	0%
unclassified Gloeobacterales	1	0%
planctomycete str.	1	0%

Species	Number of reads	Relative abundance
unclassified Saccharothrix	1	0%
Bacillus neizhouensis	1	0%
Cytophaga aurantiaca	1	0%
unclassified Labrys	1	0%
Mycobacterium alsiensis	1	0%
Massilia varians	1	0%
Staphylococcus saprophyticus	1	0%
Streptococcus parauberis	1	0%
Ramlibacter tataouinensis	1	0%
unclassified Tepidimonas	1	0%
unclassified Candidatus Nardonella	1	0%
Lactobacillus graminis	1	0%
unclassified Alistipes	1	0%
Pedobacter ruber	1	0%
unclassified Carnobacteriaceae	1	0%
Mycobacterium chlorophenolicum	1	0%
unclassified Lewinella	1	0%
Ochrobactrum intermedium	1	0%
Massilia lurida	1	0%
Novosphingobium pentaromativorans	1	0%
Nocardioides plantarum	1	0%
Kaistia geumhonensis	1	0%
Anabaenopsis circularis	1	0%
Hymenobacter actinosclerus	1	0%
Lactobacillus manihotivorans	1	0%
unclassified Eukaryota	1	0%
Leucobacter aridicollis	1	0%
unclassified Carnobacterium	1	0%
Mycobacterium pallens	1	0%
Methylobacterium organophilum	1	0%
Devosia yakushimensis	1	0%
Mucilaginibacter dorajii	1	0%
Devosia glacialis	1	0%
Allokutzneria multivorans	1	0%
unclassified Winogradskyella	1	0%
unclassified Methylacidiphilae	1	0%
unclassified Sulfuricella	1	0%
Roseomonas aerophila	1	0%
unclassified Tabrizicola	1	0%
unclassified Edwardsiella	1	0%
unclassified Cryptosporangium	1	0%
Microbacterium arborescens	1	0%
Alicyclobacillus pomorum	1	0%
unclassified Oscillospira	1	0%
Kineococcus radiotolerans	1	0%
unclassified Trichormus	1	0%
unclassified Euzebyales	1	0%
unclassified Micavibrio	1	0%
Paracoccus aminovorans	1	0%
Pedobacter kwangyangensis	1	0%

Species	Number of reads	Relative abundance
unclassified Azotobacter	1	0%
Lactobacillus concavus	1	0%
Saccharomonospora viridis	1	0%
unclassified Vulgatibacteraceae	1	0%
Pseudolabrys taiwanensis	1	0%
unclassified Cryomorpha	1	0%
Afipia genosp.	1	0%
Paenibacillus pinisoli	1	0%
unclassified Diaminobutyricimonas	1	0%
Jeotgalibacillus soli	1	0%
Microbacterium murale	1	0%
unclassified Kibdelosporangium	1	0%
Polaromonas rhizosphaerae	1	0%
Pseudomonas kuykendallii	1	0%
Paenisporosarcina indica	1	0%
Arthrobacter chlorophenolicus	1	0%
unclassified Defluviimonas	1	0%
unclassified Marininema	1	0%
Devosia psychrophila	1	0%
Myxococcus virescens	1	0%
Wohlfahrtiimonas chitiniclastica	1	0%
unclassified Ardenscatena	1	0%
unclassified Formosa	1	0%
Novosphingobium stygium	1	0%
Clostridium aminovalericum	1	0%
Micromonospora pattaloongensis	1	0%
unclassified Catenuloplanes	1	0%
Geobacillus thermodenitrificans	1	0%
Paenibacillus barengoltzii	1	0%
Bartramia environmental	1	0%
Romboutsia lituseburensis	1	0%
Stenotrophomonas geniculata	1	0%
Comamonas testosteroni	1	0%
Ornithinibacter aureus	1	0%
Sphingomonas soli	1	0%
unclassified Starkeya	1	0%
Streptomyces glaucescens	1	0%
Bacillus ginsenggisoli	1	0%
Rickettsiella costelytrae	1	0%
Stenotrophomonas chelatiphaga	1	0%
Lysobacter soli	1	0%
Streptomyces griseus	1	0%
Ammoniphilus oxalivorans	1	0%
unclassified Tomitella	1	0%
unclassified Sphingopyxis	1	0%
Roseomonas aquatica	1	0%
Nocardioides daphniae	1	0%
Clostridium thermopalmarium	1	0%
unclassified Gaetbulibacter	1	0%
Geobacter bremensis	1	0%

Species	Number of reads	Relative abundance
Pedobacter ginsengisoli	1	0%
unclassified Nocardiopsaceae	1	0%
unclassified Dolichospermum	1	0%
Microvirga guangxiensis	1	0%
unclassified Trebouxiophyceae	1	0%
unclassified Sporanaerobacter	1	0%
unclassified Tepidimicrobium	1	0%
unclassified Proteiniclasticum	1	0%
Chitinophaga pinensis	1	0%
unclassified Sorangium	1	0%
unclassified Gelidibacter	1	0%
Aeromicrobium fastidiosum	1	0%
Devosia epidermidihirudinis	1	0%
Halomonas meridiana	1	0%
Methylobacter luteus	1	0%
Mycobacterium madagascariense	1	0%
unclassified Clostridiales Family XI. Incertae Sedis	1	0%
Nocardioides ginsengisegetis	1	0%
Clostridium thermosuccinogenes	1	0%
unclassified Brucellaceae	1	0%
unclassified Bifidobacterium	1	0%
Flavobacterium xinjiangense	1	0%
unclassified Collimonas	1	0%
Flavobacterium sinopsychrotolerans	1	0%
unclassified Rhodocytophaga	1	0%
Halomonas axialensis	1	0%
Alkalibaculum bacchi	1	0%
Bacillus oceanisediminis	1	0%
unclassified Reyranella	1	0%
Bacillus azotoformans	1	0%
Paenibacillus chitinolyticus	1	0%
Achromobacter insuavis	1	0%
unclassified Xylophilus	1	0%
unclassified Shewanella	1	0%
unclassified Planotetraspora	1	0%
unclassified Zygnemophyceae	1	0%
Roseimicrobium gellanilyticum	1	0%
unclassified Neisseriales	1	0%
Nostoc carneum	1	0%
unclassified Roseospira	1	0%
Micromonospora siamensis	1	0%
Nocardioides panacisoli	1	0%
Caldibacillus debilis	1	0%
Arthrobacter niigatensis	1	0%
Pseudomonas litoralis	1	0%
unclassified Oligoflexales	1	0%
unclassified Oligoflexia	1	0%
Bacillus infantis	1	0%
unclassified Acetobacterium	1	0%
Pseudomonas fragi	1	0%

Species	Number of reads	Relative abundance
Cellulosilyticum lentocellum	1	0%
Pedobacter koreensis	1	0%
Bacillus acidicola	1	0%
Tomitella biformata	1	0%
Devosia neptuniae	1	0%
Pelomonas saccharophila	1	0%
unclassified Lechevalieria	1	0%
unclassified Luteimonas	1	0%
Weissella paramesenteroides	1	0%
unclassified Thermopolyspora	1	0%
Shinella kummerowiae	1	0%
Zhihengliuella salsuginis	1	0%
Bacillus simplex	1	0%
Arthrobacter sulfureus	1	0%
Bdellovibrio bacteriovorus	1	0%
Herbaspirillum autotrophicum	1	0%
Chryseobacterium piscicola	1	0%
Mesorhizobium alhagi	1	0%
unclassified Chlorobia	1	0%
Arthrobacter nicotianae	1	0%
unclassified Corynebacteriaceae	1	0%
Bacillus coagulans	1	0%
Streptomyces diastaticus	1	0%
Comamonas denitrificans	1	0%
Clostridium algidixylanolyticum	1	0%
Chryseobacterium carnis	1	0%
Bacillus cytotoxicus	1	0%
Williamsia phyllosphaerae	1	0%
Ureibacillus thermophilus	1	0%
unclassified Lactobacillus	1	0%
unclassified Symbiobacterium	1	0%
unclassified Pimelobacter	1	0%
unclassified Owenweeksia	1	0%
Arthrobacter globiformis	1	0%
Streptomyces malaysiensis	1	0%
Mycobacterium fluoranthenivorans	1	0%
unclassified Lactobacillaceae	1	0%
Agromyces albus	1	0%
Bacillus plakortidis	1	0%
Saccharothrix texasensis	1	0%
Trichormus variabilis	1	0%
Chlamydiales endosymbiont	1	0%
Verrucosispora sediminis	1	0%
Sugarcane phytoplasma	1	0%
Hydrogenophaga taeniospiralis	1	0%
Vaccinium corymbosum	1	0%
unclassified Sufflavibacter	1	0%
unclassified Chlamydomonadaceae	1	0%
unclassified Pigmentiphaga	1	0%
Promicromonospora umidemergens	1	0%

Species	Number of reads	Relative abundance
unclassified Achromobacter	1	0%
Chloroidium saccharophilum	1	0%
unclassified Parasegetibacter	1	0%
unclassified Cellulosimicrobium	1	0%
Luteibacter rhizovicinus	1	0%
Marmoricola korecus	1	0%
unclassified Brevibacillus	1	0%
unclassified Methanomicrobiaceae	1	0%
unclassified Christensenellaceae	1	0%
Clostridium subterminale	1	0%
Noviherbaspirillum aurantiacum	1	0%
unclassified Coriobacteriia	1	0%
Pseudomonas borealis	1	0%
Adhaeribacter terreus	1	0%
Terrimonas ferruginea	1	0%
Blastocatella fastidiosa	1	0%
Candidatus Halomonas	1	0%
Methylobacter marinus	1	0%
Bacillus thermoamylovorans	1	0%
Lelliottia amnigena	1	0%
Pseudomonas jessenii	1	0%
Bacillus anthracis	1	0%
Rhodobacter sphaeroides	1	0%
unclassified Pelagibacterium	1	0%
Microbacterium takaoensis	1	0%
unclassified Marispirillum	1	0%
Clostridium senegalense	1	0%
Cohnella arctica	1	0%
Achromobacter xylosoxidans	1	0%
Cohnella soli	1	0%
Arenimonas oryziterrae	1	0%
Turicella otitidis	1	0%
unclassified Rufibacter	1	0%
Comamonas terrigena	1	0%
unclassified Oceanicola	1	0%
Caryophanon latum	1	0%
Solibacillus silvestris	1	0%
unclassified Coriobacteriaceae	1	0%
unclassified Blechnaceae	1	0%
Chryseobacterium aahli	1	0%
Thermomonas brevis	1	0%
Mycobacterium arupense	1	0%
unclassified Aquamicrobium	1	0%
Actinocorallia aurantiaca	1	0%
Streptomyces ferralitis	1	0%
unclassified Fontibacillus	1	0%
unclassified Aequorivita	1	0%
Bacillus acidiceler	1	0%
Thelypteris environmental	1	0%
unclassified Gluconacetobacter	1	0%

Species	Number of reads	Relative abundance
unclassified Methanomassiliicoccus	1	0%
unclassified Actinocatenispora	1	0%
unclassified Candidatus Lumbricincola	1	0%
Bacillus lentus	1	0%
Labrys methylaminiphilus	1	0%
Sporosarcina ureae	1	0%
unclassified Frigoribacterium	1	0%
Chryseobacterium anthropi	1	0%
Faecalibacterium prausnitzii	1	0%
unclassified Sanguibacteraceae	1	0%
Telmatobacter bradus	1	0%
	15904 (93212 total)	
