# Supplementary material for: Microbial Diversity and Antimicrobial Resistance Profile in Microbiota From Soils of Conventional and Organic Farming Systems
Source: Front Microbiol. 2019 Apr 26;10:892. doi: 10.3389/fmicb.2019.00892 (PMC6498881; doi:10.3389/fmicb.2019.00892)
Supplement: Supplementary file 5 [file Data_Sheet_2.PDF]

All bacterial species from the soil of conventional farming

Species	Number of reads	Relative abundance
unclassified Acidobacterium	6889	3.57%
unclassified Gemmatimonas	3702	1.91%
unclassified Candidatus Saccharibacteria	2866	1.48%
unclassified Betaproteobacteria	2852	1.47%
unclassified Bacteria	2708	1.4%
unclassified Acidobacteriaceae	2682	1.39%
unclassified Bacillus	2548	1.32%
unclassified Alphaproteobacteria	2539	1.31%
unclassified Deltaproteobacteria	2191	1.13%
Bacillus longiquaesitum	2181	1.13%
unclassified Nitrospira	1910	0.98%
unclassified Planctomycetales	1866	0.96%
unclassified Hyphomicrobium	1807	0.93%
unclassified Gammaproteobacteria	1758	0.91%
unclassified Holophaga	1693	0.87%
unclassified Gemmatimonadales	1553	0.8%
unclassified Actinobacteria	1528	0.79%
unclassified Pirellulaceae	1496	0.77%
unclassified Chitinophagaceae	1470	0.76%
unclassified Arthrobacter	1466	0.75%
unclassified Longilinea	1436	0.74%
Arthrobacter pascens	1378	0.71%
unclassified Sphaerobacteridae	1343	0.69%
unclassified Myxococcales	1261	0.65%
unclassified Geobacter	1252	0.64%
unclassified Verrucomicrobiales	1228	0.63%
unclassified Gemmatimonadaceae	1193	0.61%
unclassified Sphingomonas	1177	0.61%
unclassified Agrobacterium	1157	0.59%
unclassified Chitinophaga	1141	0.59%
unclassified Flavobacterium	1069	0.55%
unclassified Rhizobiales	1067	0.55%
unclassified Nocardioides	1052	0.54%
unclassified Ferruginibacter	1029	0.53%
unclassified Holophagaceae	1008	0.52%
Bradyrhizobium elkanii	995	0.51%
unclassified Acidobacteriales	945	0.48%
unclassified Actinomycetales	924	0.47%
unclassified Rhizobium	880	0.45%
unclassified Cytophagaceae	874	0.45%
alpha proteobacterium	834	0.43%
unclassified Rhodoplanes	830	0.43%
unclassified Chthoniobacteraceae	810	0.41%
unclassified Actinobacteridae	789	0.4%
unclassified Devosia	788	0.4%
unclassified Pedobacter	768	0.39%
unclassified Candidatus Solibacter	763	0.39%
unclassified Anaerolineae	760	0.39%
Flavobacterium succinicans	757	0.39%
unclassified Ilumatobacter	757	0.39%

Species	Number of reads	Relative abundance
unclassified Paenibacillus	737	0.38%
unclassified Planctomycetaceae	736	0.38%
unclassified Chloroflexus	728	0.37%
Unclassified	721	0.37%
unclassified Rubrobacteridae	713	0.36%
unclassified Clostridiales	703	0.36%
unclassified Terrimonas	700	0.36%
unclassified Citricoccus	697	0.36%
unclassified Nitrosospira	694	0.35%
unclassified Anaerolinaceae	667	0.34%
metal-contaminated soil	659	0.34%
unclassified Conexibacteraceae	652	0.33%
unclassified Chlamydiales	650	0.33%
unclassified Mycobacterium	640	0.33%
Clostridium bowmanii	631	0.32%
unclassified Holophagales	621	0.32%
unclassified Desulfuromonadales	613	0.31%
unclassified rosids	609	0.31%
unclassified Pirellula	607	0.31%
unclassified Nordella	601	0.31%
unclassified Sphingobacteriales	591	0.3%
unclassified Streptomyces	588	0.3%
unclassified Pedomicrobium	583	0.3%
Bacillus muralis	572	0.29%
unclassified Dehalococcoidaceae	563	0.29%
unclassified Planctomyces	558	0.28%
unclassified Solirubrobacter	551	0.28%
unclassified Gemmatimonadetes	538	0.27%
unclassified Clostridia	534	0.27%
unclassified Sphingobacteriaceae	534	0.27%
unclassified Iamia	530	0.27%
unclassified Chloroflexi	516	0.26%
unclassified Bacillales	515	0.26%
unclassified Plectosphaerellaceae	515	0.26%
unclassified Candidatus Microthrix	511	0.26%
unclassified Conexibacter	505	0.26%
unclassified Niastella	501	0.25%
unclassified Opitutus	498	0.25%
unclassified Cellvibrio	495	0.25%
Devosia ginsengisoli	493	0.25%
Streptomyces reticuliscabiei	491	0.25%
unclassified Dehalococcoidales	491	0.25%
unclassified Clostridium	491	0.25%
unclassified Bacilli	488	0.25%
unclassified Frankia	466	0.24%
unclassified Burkholderiales	462	0.23%
unclassified Fibrobacteria	459	0.23%
unclassified Roseiflexus	454	0.23%
unclassified Anaerolineales	445	0.23%
unclassified Levilinea	443	0.22%

Species	Number of reads	Relative abundance
unclassified Thiorhodospira	441	0.22%
unclassified Xanthomonadaceae	439	0.22%
unclassified Hydrogenophaga	437	0.22%
unclassified Pseudomonadaceae	431	0.22%
unclassified Gaiella	428	0.22%
unclassified Rhodocyclales	425	0.22%
unclassified Sporosarcina	424	0.21%
Sphingomonas jaspsi	423	0.21%
unclassified Geobacteraceae	421	0.21%
unclassified Verrucomicrobiaceae	420	0.21%
Knoellia subterranea	415	0.21%
unclassified Ferrimicrobium	414	0.21%
unclassified Nitrosomonadaceae	413	0.21%
unclassified Chloroflexales	413	0.21%
unclassified Burkholderia	410	0.21%
unclassified Flavobacteriales	407	0.21%
Massilia aurea	406	0.21%
Salinibacterium amurskyense	404	0.2%
unclassified Mucilaginibacter	397	0.2%
unclassified Flexibacter	392	0.2%
unclassified Pseudolabrys	386	0.2%
unclassified Acidobacteriia	385	0.19%
unclassified Pseudomonadales	381	0.19%
unclassified Syntrophobacteraceae	379	0.19%
unclassified Sphaerobacter	376	0.19%
beta proteobacterium	374	0.19%
Pedobacter panaciterrae	372	0.19%
unclassified Chondromyces	364	0.18%
unclassified Roseiflexales	364	0.18%
unclassified Caldilinea	364	0.18%
unclassified Rhizobiaceae	362	0.18%
unclassified Gaiellaceae	353	0.18%
unclassified Massilia	352	0.18%
unclassified Gemmataceae	349	0.18%
unclassified Aciditerrimonas	348	0.18%
unclassified Burkholderiaceae	344	0.17%
unclassified Acidimicrobidae	343	0.17%
unclassified Gemmata	335	0.17%
unclassified Pseudonocardia	332	0.17%
unclassified Steroidobacter	332	0.17%
unclassified Janthinobacterium	329	0.17%
unclassified Cytophagales	326	0.16%
unclassified Rhodospirillaceae	323	0.16%
unclassified Xanthomonadales	323	0.16%
unclassified Planctomycetia	322	0.16%
Rhodococcus fascians	322	0.16%
unclassified Luteolibacter	311	0.16%
unclassified Arenimonas	311	0.16%
unclassified Mesorhizobium	310	0.16%
unclassified Pirellulales	309	0.16%

Species	Number of reads	Relative abundance
unclassified Hypocreomycetidae	305	0.15%
unclassified Flavobacteriaceae	304	0.15%
unclassified Hyphomicrobiaceae	303	0.15%
unclassified Herbaspirillum	303	0.15%
unclassified Nitrospirales	300	0.15%
unclassified Caldilineaceae	300	0.15%
unclassified Knoellia	298	0.15%
unclassified Kouleothrix	295	0.15%
Sphingomonas faeni	290	0.15%
unclassified Bacillaceae	288	0.14%
unclassified Oxalobacteraceae	285	0.14%
unclassified Cyanobacterium	281	0.14%
unclassified Dongia	281	0.14%
unclassified Sphingomonadales	280	0.14%
unclassified Methylosinus	277	0.14%
unclassified Vitaceae	275	0.14%
unclassified Methylibium	274	0.14%
unclassified Prolixibacter	270	0.13%
unclassified Haliangiaceae	270	0.13%
Streptomyces mirabilis	267	0.13%
unclassified Sphingobacteriia	262	0.13%
unclassified Bdellovibrio	262	0.13%
unclassified Comamonadaceae	260	0.13%
Pseudoxanthomonas yeongjuensis	255	0.13%
Nitrospira cf.	251	0.13%
unclassified Saccharibacillus	249	0.12%
unclassified Adhaeribacter	247	0.12%
unclassified Bradyrhizobium	245	0.12%
unclassified Phycisphaerales	244	0.12%
unclassified Haliangium	242	0.12%
unclassified Lysinibacillus	241	0.12%
unclassified Legionellales	240	0.12%
unclassified Neisseriaceae	240	0.12%
unclassified Acidimicrobiaceae	239	0.12%
unclassified Dokdonella	238	0.12%
unclassified Syntrophobacterales	236	0.12%
unclassified Nocardioidaceae	234	0.12%
unclassified Iamiaceae	232	0.12%
unclassified Ruminococcaceae	230	0.11%
unclassified Terriglobus	226	0.11%
unclassified Polyangiaceae	225	0.11%
unclassified Verticillium	222	0.11%
Pseudomonas putida	222	0.11%
unclassified Rhodococcus	221	0.11%
Aeromicrobium ginsengisoli	221	0.11%
Nakamurella flavida	220	0.11%
Phycicola gilvus	218	0.11%
Sporosarcina globispora	218	0.11%
unclassified Paenibacillaceae	216	0.11%
unclassified Sinobacteraceae	215	0.11%

Species	Number of reads	Relative abundance
unclassified Bosea	213	0.11%
unclassified Flavisolibacter	213	0.11%
unclassified Coxiellaceae	210	0.1%
Rhodanobacter spathiphylli	210	0.1%
Fluviicola taffensis	206	0.1%
Hyphomicrobium facile	205	0.1%
Lysinibacillus sphaericus	204	0.1%
Variovorax paradoxus	202	0.1%
unclassified Nitrosomonadales	199	0.1%
unclassified Solirubrobacteraceae	197	0.1%
unclassified Flavitalea	197	0.1%
unclassified Solirubrobacterales	197	0.1%
unclassified Pelobacter	196	0.1%
Psychrobacillus psychrodurans	195	0.1%
unclassified Ohtaekwangia	195	0.1%
unclassified Nitrospiraceae	194	0.1%
Mycobacterium goodii	194	0.1%
unclassified Rikenellaceae	192	0.09%
Pseudomonas veronii	190	0.09%
unclassified Pedosphaerales	190	0.09%
Kaistobacter terrae	190	0.09%
unclassified Haliscomenobacter	189	0.09%
unclassified Proteiniclasticum	189	0.09%
unclassified Bdellovibrionales	189	0.09%
unclassified Anaerolineaceae	185	0.09%
unclassified Pedosphaeraceae	185	0.09%
unclassified Kaistobacter	183	0.09%
unclassified Byssovorax	183	0.09%
unclassified Acidothermus	182	0.09%
unclassified Epsilonproteobacteria	182	0.09%
unclassified Lysobacter	180	0.09%
unclassified Opitutaceae	180	0.09%
unclassified Cyclobacteriaceae	178	0.09%
unclassified Marmoricola	178	0.09%
unclassified Methylobacterium	174	0.09%
unclassified Pseudomonas	174	0.09%
Herbaspirillum autotrophicum	173	0.08%
unclassified Rhodospirillales	173	0.08%
unclassified Alcaligenaceae	172	0.08%
unclassified Gemmatales	170	0.08%
unclassified Variovorax	170	0.08%
Bosea genosp.	170	0.08%
Arthrobacter psychrolactophilus	170	0.08%
gamma proteobacterium	169	0.08%
unclassified Nakamurella	169	0.08%
Sporosarcina soli	169	0.08%
Bacillus fumarioli	168	0.08%
unclassified Dehalococcoidetes	167	0.08%
Nocardioides mesophilus	166	0.08%
unclassified Anaerolinea	166	0.08%

Species	Number of reads	Relative abundance
unclassified Myxococcaceae	165	0.08%
unclassified Bacteriovoracaceae	165	0.08%
unclassified Acidobacteria	164	0.08%
unclassified Kouleothrixaceae	164	0.08%
unclassified Actinomyces	164	0.08%
Sphingomonas asaccharolytica	164	0.08%
unclassified Clostridiaceae	163	0.08%
unclassified Thiobacillus	161	0.08%
unclassified Methylocystis	160	0.08%
unclassified Alteromonadales	160	0.08%
Mycobacterium vaccae	160	0.08%
unclassified Thermomicrobia	159	0.08%
unclassified Sphingomonadaceae	159	0.08%
unclassified Bacteroidales	158	0.08%
unclassified Cyanobacteria	155	0.08%
unclassified Ornithinicoccus	154	0.07%
unclassified Singulisphaera	154	0.07%
unclassified Geodermatophilaceae	153	0.07%
unclassified Solibacterales	153	0.07%
unclassified Nitrosovibrio	153	0.07%
unclassified Saprospirales	151	0.07%
unclassified Saprospiraceae	151	0.07%
Pseudoxanthomonas ginsengisoli	150	0.07%
Chelatococcus asaccharovorans	149	0.07%
Devosia limi	148	0.07%
Lysobacter oligotrophicus	147	0.07%
unclassified Sphaerobacteraceae	147	0.07%
unclassified Microvirga	145	0.07%
Streptosporangium amethystogenes	145	0.07%
Bacillus niacini	142	0.07%
unclassified Proteobacteria	141	0.07%
Rhodanobacter thiooxydans	141	0.07%
unclassified Spartobacteria	141	0.07%
Cryocola poae	141	0.07%
Kaistia granuli	140	0.07%
unclassified Sporichthya	140	0.07%
unclassified Rhodanobacter	139	0.07%
Ensifer adhaerens	139	0.07%
Pedobacter duraquae	139	0.07%
Bacillus thuringiensis	138	0.07%
Nocardioides islandensis	138	0.07%
unclassified Afifella	137	0.07%
Chryseobacterium gregarium	137	0.07%
unclassified Lachnoclostridium	137	0.07%
unclassified Chlamydiia	136	0.07%
unclassified Fimbriimonas	135	0.06%
unclassified Rubrobacteria	135	0.06%
unclassified Rubrobacterales	135	0.06%
Sanguibacter inulinus	134	0.06%
unclassified Erythromicrobium	134	0.06%

Species	Number of reads	Relative abundance
unclassified Thermoleophilales	132	0.06%
unclassified Desulfovibrio	132	0.06%
Janthinobacterium agaricidamnosum	131	0.06%
unclassified Planctomycetes	131	0.06%
Brevundimonas nasdae	131	0.06%
Methylobacter luteus	130	0.06%
Sporosarcina ginsengisoli	130	0.06%
unclassified Acidimicrobiales	127	0.06%
unclassified Bdellovibrionaceae	126	0.06%
unclassified Flavobacteriia	125	0.06%
unclassified Syntrophaceae	125	0.06%
Arthrobacter crystallopoietes	123	0.06%
unclassified Parachlamydiaceae	123	0.06%
Luteimonas terricola	122	0.06%
unclassified Salinibacterium	122	0.06%
unclassified Pezizales	122	0.06%
unclassified Caldilineales	121	0.06%
unclassified Acidovorax	120	0.06%
unclassified Aridibacter	120	0.06%
unclassified Nannocystis	119	0.06%
unclassified Cryomorphaceae	119	0.06%
unclassified Blastochloris	119	0.06%
unclassified Acetobacteraceae	119	0.06%
unclassified Staphylococcus	119	0.06%
unclassified Micrococcaceae	118	0.06%
unclassified Lachnospiraceae	118	0.06%
unclassified Thiobacter	117	0.06%
unclassified Thermoleophilum	116	0.06%
Clavibacter michiganensis	116	0.06%
Rhizobium leguminosarum	116	0.06%
unclassified Cupriavidus	114	0.05%
unclassified Pasteuriaceae	113	0.05%
unclassified Thermomonas	113	0.05%
unclassified Candidatus Nitrososphaera	113	0.05%
unclassified Micromonosporaceae	112	0.05%
Nitrospira japonica	111	0.05%
Rhodococcus globerulus	111	0.05%
unclassified Polyangium	111	0.05%
unclassified Brevundimonas	110	0.05%
unclassified Taibaiella	110	0.05%
unclassified Methylobacteriaceae	109	0.05%
unclassified Rhodothermales	108	0.05%
unclassified Beggiatoa	108	0.05%
unclassified Anaeromyxobacter	108	0.05%
Blastococcus aggregatus	106	0.05%
Devosia yakushimensis	106	0.05%
unclassified Fimbriimonadaceae	106	0.05%
unclassified Synechococcophycideae	104	0.05%
unclassified Candidatus Koribacter	104	0.05%
Nocardioides oleivorans	104	0.05%

Species	Number of reads	Relative abundance
unclassified Micromonospora	103	0.05%
unclassified Derxia	102	0.05%
Microbacterium chocolatum	101	0.05%
unclassified Glycomyces	100	0.05%
Mycobacterium frederiksbergense	100	0.05%
unclassified Bauldia	99	0.05%
Cellulomonas xylanilytica	99	0.05%
unclassified Desulfosporosinus	99	0.05%
unclassified Campylobacterales	99	0.05%
Paenibacillus chondroitinus	99	0.05%
unclassified Sphingobium	98	0.05%
Pedobacter steynii	98	0.05%
unclassified Leptothrix	98	0.05%
unclassified Cystobacter	97	0.05%
unclassified Legionella	96	0.04%
Aquabacterium commune	96	0.04%
unclassified Rubrivivax	96	0.04%
unclassified Cystobacteraceae	95	0.04%
unclassified Dehalococcoides	95	0.04%
Mesorhizobium chacoense	95	0.04%
Nitrosovibrio tenuis	94	0.04%
unclassified Thiotrichaceae	94	0.04%
unclassified Fusobacterium	93	0.04%
unclassified Sedimentibacter	93	0.04%
unclassified Microbacterium	93	0.04%
Rhodanobacter lindaniclasticus	93	0.04%
unclassified Spirobacillales	92	0.04%
Nocardioides iriomotensis	92	0.04%
Lysobacter pocheonensis	92	0.04%
Sphingopyxis alaskensis	92	0.04%
unclassified Cytophagia	91	0.04%
Mesorhizobium amorphae	91	0.04%
unclassified Myxococcus	91	0.04%
Bacillus nealsonii	90	0.04%
unclassified Asticcacaulis	90	0.04%
unclassified Prosthecobacter	88	0.04%
unclassified Chthoniobacter	88	0.04%
Marmoricola aequoreus	87	0.04%
unclassified Rhizomicrobium	87	0.04%
unclassified Acidimicrobium	86	0.04%
unclassified Chloroflexaceae	86	0.04%
Pseudomonas mandelii	85	0.04%
unclassified Streptomycetaceae	85	0.04%
Desulfosporosinus meridiei	85	0.04%
unclassified Sporichthyaceae	84	0.04%
unclassified Dyadobacter	84	0.04%
Lysinibacillus boronitolerans	83	0.04%
Pedobacter agri	83	0.04%
unclassified Rhodocyclaceae	83	0.04%
unclassified Ectothiorhodospiraceae	83	0.04%

Species	Number of reads	Relative abundance
unclassified Verrucosispora	83	0.04%
unclassified Phaselicystidaceae	82	0.04%
unclassified Aquabacterium	82	0.04%
unclassified Novosphingobium	82	0.04%
Clostridium ruminantium	82	0.04%
Nonomuraea candida	82	0.04%
unclassified Actinoplanes	81	0.04%
unclassified Solibacillus	81	0.04%
unclassified Thermoleophilaceae	81	0.04%
unclassified Nannocystaceae	81	0.04%
unclassified Povalibacter	80	0.04%
unclassified Saprospirae	80	0.04%
Micromonospora saelicesensis	80	0.04%
unclassified Hymenobacter	79	0.04%
Pedobacter caeni	79	0.04%
unclassified Flammeovirgaceae	79	0.04%
Lentzea albidocapillata	79	0.04%
unclassified Thermoanaerobacterales	79	0.04%
Nonomuraea maheshkhaliensis	79	0.04%
unclassified Oxobacter	79	0.04%
unclassified Streptophyta	79	0.04%
Phyllobacterium myrsinacearum	79	0.04%
unclassified Aeromicrobium	79	0.04%
Flavobacterium tiangeerense	78	0.04%
unclassified Gracilibacter	78	0.04%
unclassified Pseudoxanthomonas	78	0.04%
unclassified Alicyclobacillus	78	0.04%
Paenibacillus amylolyticus	77	0.03%
Rhodoferax ferrireducens	77	0.03%
Pseudomonas viridiflava	77	0.03%
unclassified Fluviicola	77	0.03%
unclassified Phyllobacteriaceae	77	0.03%
unclassified Chthoniobacterales	77	0.03%
unclassified Rhodomicrobium	76	0.03%
unclassified Chromatiales	76	0.03%
unclassified Lactobacillales	76	0.03%
Pedobacter borealis	76	0.03%
Nocardioides halotolerans	76	0.03%
Flavobacterium psychrolimnae	76	0.03%
Paenibacillus castaneae	75	0.03%
unclassified Georgfuchsia	75	0.03%
Bacillus firmus	73	0.03%
Lysobacter ginsengisoli	73	0.03%
Povalibacter uvarum	73	0.03%
Pseudomonas syringae	73	0.03%
Pseudomonas umsongensis	72	0.03%
Rhodanobacter fulvus	72	0.03%
Catellatospora citrea	72	0.03%
unclassified Pasteuria	72	0.03%
unclassified Tissierella	71	0.03%

Species	Number of reads	Relative abundance
Skermanella aerolata	71	0.03%
Mesorhizobium septentrionale	70	0.03%
unclassified Sordariomycetes	70	0.03%
unclassified Bradyrhizobiaceae	70	0.03%
unclassified Geothrix	69	0.03%
unclassified Porphyrobacter	69	0.03%
unclassified Dechloromonas	69	0.03%
Cryobacterium psychrophilum	69	0.03%
unclassified Gallionella	69	0.03%
unclassified Vitis	69	0.03%
unclassified Legionellaceae	68	0.03%
unclassified Zavarzinella	68	0.03%
unclassified Xanthomonas	68	0.03%
unclassified Segetibacter	68	0.03%
unclassified Rubrobacteraceae	68	0.03%
actinobacterium SCGC	67	0.03%
unclassified Kribbella	67	0.03%
unclassified Chloracidobacteria	67	0.03%
unclassified Phenylobacterium	67	0.03%
Ammoniphilus oxalaticus	66	0.03%
unclassified Desulfobacterales	65	0.03%
unclassified Microthrixaceae	65	0.03%
unclassified Solibacteraceae	64	0.03%
Marmoricola bigeumensis	64	0.03%
unclassified Bacteriovorax	64	0.03%
unclassified Rickettsiales	64	0.03%
unclassified Oscillatoriales	64	0.03%
unclassified Peptococcaceae	63	0.03%
unclassified Serpens	63	0.03%
unclassified Pedosphaera	63	0.03%
Rubrivivax gelatinosus	63	0.03%
Bosea thiooxidans	63	0.03%
unclassified Bellilinea	63	0.03%
unclassified Blastococcus	63	0.03%
Sphingomonas wittichii	63	0.03%
unclassified Dehalococcoidia	62	0.03%
Bacillus asahii	62	0.03%
unclassified Desulfovirga	62	0.03%
Psychrobacillus psychrotolerans	62	0.03%
unclassified Methylocystaceae	61	0.03%
unclassified Filomicrobium	61	0.03%
unclassified Modestobacter	60	0.03%
unclassified Psychrobacillus	60	0.03%
unclassified Pelobacteraceae	60	0.03%
unclassified Sphingobacterium	60	0.03%
unclassified Azohydromonas	59	0.03%
Bacillus drentensis	59	0.03%
Bacillus murimartini	59	0.03%
unclassified Syntrophus	59	0.03%
unclassified Amaricoccus	58	0.03%

Species	Number of reads	Relative abundance
unclassified Nocardiaceae	58	0.03%
unclassified Phycisphaerae	58	0.03%
Janthinobacterium lividum	58	0.03%
unclassified Sporocytophaga	58	0.03%
Nocardioides jensenii	58	0.03%
Arthrobacter defluvii	57	0.02%
unclassified Prolixibacteraceae	57	0.02%
unclassified Cryocola	57	0.02%
Agromyces ramosus	57	0.02%
unclassified Microbacteriaceae	57	0.02%
Spirilliplanes yamanashiensis	57	0.02%
unclassified Ochrobactrum	56	0.02%
unclassified Methylotenera	56	0.02%
unclassified Candidatus Xiphinematobacter	56	0.02%
Cytophaga hutchinsonii	56	0.02%
Candidatus Nitrospira	56	0.02%
unclassified Caldilineae	56	0.02%
Stenotrophomonas rhizophila	56	0.02%
Nordella oligomobilis	55	0.02%
unclassified Caulobacterales	55	0.02%
unclassified Thiohalospira	55	0.02%
Phenylobacterium haematophilum	55	0.02%
unclassified Methylobacter	55	0.02%
unclassified Caldanaerobacter	55	0.02%
unclassified Porphyromonadaceae	55	0.02%
unclassified Kineosporia	54	0.02%
unclassified Veillonellaceae	54	0.02%
unclassified Hirschia	54	0.02%
unclassified Caulobacter	54	0.02%
Pedobacter nyackensis	54	0.02%
unclassified Cellulomonas	54	0.02%
unclassified Desulfobacteraceae	53	0.02%
unclassified Entotheonellaceae	53	0.02%
unclassified Desulforegula	53	0.02%
Hyphomicrobium vulgare	53	0.02%
unclassified Piscirickettsiaceae	53	0.02%
unclassified Verrucomicrobiae	53	0.02%
unclassified Niabella	53	0.02%
Streptomyces atratus	53	0.02%
unclassified Mycobacteriaceae	53	0.02%
Sphingomonas echinoides	52	0.02%
unclassified Koribacteraceae	52	0.02%
unclassified Shinella	52	0.02%
Solirubrobacter ginsenosidimutans	52	0.02%
unclassified Pilimelia	52	0.02%
unclassified Ferrithrix	52	0.02%
unclassified Bartonella	52	0.02%
unclassified Lentzea	51	0.02%
unclassified Phaselicystis	51	0.02%
Flavobacterium psychrophilum	51	0.02%

Species	Number of reads	Relative abundance
unclassified Bacteroidia	51	0.02%
unclassified Desulfovibrionaceae	50	0.02%
Mycobacterium pyrenivorans	50	0.02%
unclassified Microlunatus	50	0.02%
unclassified Cohnella	50	0.02%
unclassified Actinomycetaceae	50	0.02%
unclassified Clavibacter	50	0.02%
unclassified Geothermobacter	50	0.02%
unclassified Tissierella_Soehngenia	50	0.02%
Rhodanobacter umsongensis	49	0.02%
Microlunatus aurantiacus	49	0.02%
unclassified Chlorellaceae	49	0.02%
unclassified Ignavibacteria	49	0.02%
unclassified Kofleriaceae	48	0.02%
unclassified Sphingosinicella	48	0.02%
unclassified Cyanobacteriaceae	48	0.02%
unclassified Thermoanaerobacteraceae	48	0.02%
Bacillus circulans	48	0.02%
unclassified Nocardiopsis	48	0.02%
unclassified Gallionellales	47	0.02%
unclassified Isosphaeraceae	47	0.02%
unclassified Holophagae	47	0.02%
unclassified Chlorellales	47	0.02%
unclassified Phycisphaeraceae	47	0.02%
Microvirga aerilata	47	0.02%
Couchioplanes caeruleus	46	0.02%
Luteimonas aestuarii	46	0.02%
Nakamurella multipartita	46	0.02%
unclassified Hyphomonadaceae	46	0.02%
unclassified Crocinitomix	45	0.02%
unclassified Caulobacteraceae	45	0.02%
unclassified Rhodocista	45	0.02%
Streptomyces rubrolavendulae	45	0.02%
unclassified Oligoflexaceae	45	0.02%
unclassified Azospirillum	45	0.02%
unclassified Rhodobacterales	45	0.02%
Rhodococcus wratislaviensis	45	0.02%
unclassified Phaeodactylum	45	0.02%
unclassified Oscillatoriophycideae	44	0.02%
unclassified Fimbriimonadales	44	0.02%
unclassified Psychrosinus	44	0.02%
unclassified Ramlibacter	44	0.02%
unclassified Chthonomonadetes	44	0.02%
Solitalea koreensis	44	0.02%
unclassified Polaromonas	44	0.02%
Nocardioides furvisabuli	44	0.02%
unclassified Caloramator	44	0.02%
unclassified Turicibacter	44	0.02%
Pseudaminobacter defluvii	44	0.02%
unclassified Neorhizobium	43	0.02%

Species	Number of reads	Relative abundance
unclassified Acidisphaera	43	0.02%
unclassified Roseiflexaceae	43	0.02%
unclassified Rhizobacter	43	0.02%
unclassified Opitutales	43	0.02%
Chryseolinea serpens	43	0.02%
unclassified Herminiimonas	43	0.02%
unclassified Lishizhenia	43	0.02%
unclassified Archangium	43	0.02%
unclassified Roseomonas	43	0.02%
Streptomyces radiopugnans	42	0.02%
unclassified Desulfitobacterium	42	0.02%
Flavitalea populi	42	0.02%
Methylobacterium adhaesivum	42	0.02%
unclassified Erythrobacter	42	0.02%
unclassified Aquicella	41	0.02%
unclassified Flavihumibacter	41	0.02%
Chitinophaga oryziterrae	41	0.02%
Sporosarcina luteola	40	0.02%
Aliihoeflea aestuarii	40	0.02%
Arthrobacter nicotianae	40	0.02%
Bacillus gibsonii	40	0.02%
Nocardioides kribbensis	40	0.02%
unclassified Rubrobacter	40	0.02%
Massilia lurida	39	0.02%
unclassified Acetivibrio	39	0.02%
Mesorhizobium plurifarium	39	0.02%
unclassified Planococcaceae	39	0.02%
Devosia epidermidihirudinis	39	0.02%
Paenibacillus pectinilyticus	39	0.02%
unclassified Verrucomicrobium	39	0.02%
unclassified Pontibacter	39	0.02%
unclassified Thermoflavimicrobium	39	0.02%
Kribbella karoonensis	38	0.01%
Labrys wisconsinensis	38	0.01%
unclassified Coprococcus	38	0.01%
Eubacterium tenue	38	0.01%
Kineosporia rhamnosa	37	0.01%
unclassified Thermomicrobium	37	0.01%
unclassified Blastopirellula	37	0.01%
Microbacterium maritypicum	37	0.01%
unclassified Herpetosiphonales	37	0.01%
Mycobacterium hodleri	37	0.01%
unclassified Luteimonas	36	0.01%
unclassified Solibacteres	36	0.01%
Agrobacterium sullae	36	0.01%
unclassified Luedemannella	36	0.01%
Flavobacterium frigidarium	36	0.01%
unclassified Elusimicrobiales	36	0.01%
unclassified Thermoleophilia	36	0.01%
unclassified Intrasporangiaceae	36	0.01%

Species	Number of reads	Relative abundance
Asticcacaulis biprosthecium	35	0.01%
Bosea lupini	35	0.01%
unclassified Sanguibacter	35	0.01%
unclassified Catellatospora	35	0.01%
Sphingomonas kaistensis	34	0.01%
Kribbella catacumbae	34	0.01%
unclassified Malonomonas	34	0.01%
unclassified Caldicoprobacteraceae	34	0.01%
Mesorhizobium mediterraneum	34	0.01%
Bosea massiliensis	34	0.01%
Nocardioides koreensis	34	0.01%
Devosia psychrophila	34	0.01%
unclassified Oceanicella	34	0.01%
Kribbella swartbergensis	34	0.01%
Mycobacterium monacense	34	0.01%
unclassified Acholeplasmataceae	33	0.01%
unclassified Galbibacter	33	0.01%
unclassified Planococcus	33	0.01%
unclassified Rhodobium	33	0.01%
Rhodococcus tukisamuensis	33	0.01%
unclassified Terrabacter	33	0.01%
unclassified Bacteroidaceae	33	0.01%
unclassified Firmicutes	33	0.01%
unclassified Patulibacteraceae	33	0.01%
unclassified Microbispora	33	0.01%
Actinoplanes friuliensis	33	0.01%
Dyadobacter psychrophilus	33	0.01%
Microbacterium aurum	33	0.01%
unclassified Elusimicrobia	32	0.01%
unclassified Phycisphaera	32	0.01%
Pedobacter cryoconitis	32	0.01%
Chryseobacterium aahli	32	0.01%
unclassified Gracilibacteraceae	32	0.01%
unclassified Undibacterium	32	0.01%
Glycomyces harbinensis	32	0.01%
Galbibacter marinus	32	0.01%
unclassified Isosphaera	32	0.01%
unclassified Blautia	32	0.01%
Umezawaea tangerina	32	0.01%
Arenimonas oryziterrae	32	0.01%
unclassified Couchioplanes	32	0.01%
unclassified Armatimonadaceae	31	0.01%
unclassified Propionivibrio	31	0.01%
unclassified Rhodoligotrophos	31	0.01%
unclassified Chelatococcus	31	0.01%
unclassified Stella	31	0.01%
Methylobacter tundripaludum	31	0.01%
Dokdonella soli	31	0.01%
Lysobacter dokdonensis	31	0.01%
unclassified Ruminococcus	31	0.01%

Species	Number of reads	Relative abundance
Nocardioides terrigena	31	0.01%
unclassified Eubacteriaceae	31	0.01%
unclassified Anoxybacillus	31	0.01%
Pseudoduganella violaceinigra	31	0.01%
unclassified Alicyclobacillaceae	31	0.01%
Terrabacter terrae	31	0.01%
Mycobacterium cosmeticum	30	0.01%
unclassified Alteromonadaceae	30	0.01%
unclassified Polymorphospora	30	0.01%
unclassified Chthonomonadaceae	30	0.01%
unclassified Rhodothermi	30	0.01%
unclassified Filimonas	30	0.01%
Cellulosimicrobium funkei	30	0.01%
Cryocola antiquus	30	0.01%
unclassified Caldicoprobacter	30	0.01%
unclassified Rhodobiaceae	30	0.01%
Brevundimonas lenta	30	0.01%
Pedobacter boryungensis	29	0.01%
unclassified Chroococcales	29	0.01%
Microbacterium lacticum	29	0.01%
Rhodococcus kunmingensis	29	0.01%
unclassified Ensifer	29	0.01%
Nocardioides ganghwensis	29	0.01%
Devosia submarina	29	0.01%
unclassified Altererythrobacter	29	0.01%
unclassified Fusobacteriaceae	29	0.01%
unclassified Duganella	28	0.01%
unclassified Paradevosia	28	0.01%
unclassified Candidatus Chloracidobacterium	28	0.01%
unclassified Patulibacter	28	0.01%
unclassified Paludibacter	28	0.01%
unclassified Paracoccus	28	0.01%
unclassified Pedosphaerae	28	0.01%
Hyphomicrobium aestuarii	28	0.01%
unclassified Virgibacillus	28	0.01%
Devosia glacialis	28	0.01%
unclassified Kaistia	28	0.01%
Rhizocola hellebori	27	0.01%
Paenibacillus sacheonensis	27	0.01%
unclassified Cytophaga	27	0.01%
unclassified Peptoclostridium	27	0.01%
Clostridium pasteurianum	27	0.01%
Chitinophaga pinensis	27	0.01%
Cohnella lupini	27	0.01%
unclassified Gallionellaceae	27	0.01%
Mycobacterium anthracenicum	27	0.01%
unclassified Desulfuromonadaceae	27	0.01%
unclassified Krasilnikovia	26	0.01%
unclassified Parapusillimonas	26	0.01%
Aeromicrobium panaciterrae	26	0.01%

Species	Number of reads	Relative abundance
unclassified Dactylosporangium	26	0.01%
Dactylosporangium darangshiense	26	0.01%
Sphaerisporangium melleum	26	0.01%
unclassified Prosthecomicrobium	26	0.01%
unclassified Desulfovibrionales	26	0.01%
Streptomyces guanduensis	26	0.01%
unclassified Beijerinckiaceae	26	0.01%
Microlunatus panaciterrae	26	0.01%
Paenibacillus sepulcri	25	0.01%
unclassified Agrococcus	25	0.01%
Methylotenera mobilis	25	0.01%
Elbe River	25	0.01%
Mesorhizobium australicum	25	0.01%
unclassified Thermobacula	25	0.01%
unclassified Thermoactinomycetaceae	25	0.01%
unclassified Nakamurellaceae	25	0.01%
Agromyces iriomotensis	25	0.01%
Polaromonas naphthalenivorans	25	0.01%
Acetivibrio cellulolyticus	25	0.01%
Nocardia cummidelens	25	0.01%
unclassified Thermoplasmata	25	0.01%
unclassified Rivibacter	25	0.01%
Trichococcus patagoniensis	25	0.01%
unclassified Blastomonas	25	0.01%
unclassified Candidatus Nitrotoga	25	0.01%
unclassified Acidothermaceae	25	0.01%
Oryzihumus leptocrescens	25	0.01%
unclassified Streptosporangiaceae	25	0.01%
Sanguibacter antarcticus	24	0.01%
Caulobacter henricii	24	0.01%
Verrucosispora gifhornensis	24	0.01%
unclassified Desulfocaldus	24	0.01%
Mycobacterium rhodesiae	24	0.01%
unclassified Agromyces	24	0.01%
unclassified Methanomassiliicoccus	24	0.01%
unclassified Marinicellaceae	24	0.01%
unclassified Chlorobi	24	0.01%
unclassified Aetherobacter	24	0.01%
unclassified Faecalibacterium	24	0.01%
unclassified Sphingopyxis	24	0.01%
Phenylobacterium immobile	24	0.01%
Leifsonia bigeumensis	24	0.01%
unclassified Pseudonocardiaceae	24	0.01%
Paenisporosarcina indica	24	0.01%
Dokdonella ginsengisoli	24	0.01%
Arthrobacter sulfureus	24	0.01%
unclassified Hydrogenophilaceae	23	0.01%
unclassified Geodermatophilus	23	0.01%
Peptoclostridium difficile	23	0.01%
Methylibium petroleiphilum	23	0.01%

Species	Number of reads	Relative abundance
Corallococcus exiguus	23	0.01%
Aquabacterium citratiphilum	23	0.01%
unclassified Syntrophomonadaceae	23	0.01%
Rhizobium loessense	23	0.01%
Caulobacter mirabilis	23	0.01%
unclassified Zhihengliuella	23	0.01%
Turicibacter sanguinis	23	0.01%
Desmonostoc muscorum	23	0.01%
Mesorhizobium loti	23	0.01%
unclassified Armatimonadetes	23	0.01%
unclassified Herpetosiphon	23	0.01%
unclassified Xanthobacteraceae	23	0.01%
Mesorhizobium ciceri	23	0.01%
Trichococcus pasteurii	22	0.01%
Faecalibacterium prausnitzii	22	0.01%
unclassified Nostocales	22	0.01%
unclassified Methylophilaceae	22	0.01%
unclassified Parvarchaea	22	0.01%
unclassified Chryseobacterium	22	0.01%
unclassified Rhodoferax	22	0.01%
Stenotrophomonas retroflexus	22	0.01%
unclassified Blastocatella	22	0.01%
Citricoccus alkalitolerans	22	0.01%
Massilia timonae	22	0.01%
unclassified Curtobacterium	22	0.01%
Kaistia terrae	22	0.01%
Nitrosospira briensis	21	0.01%
Nocardioides aestuarii	21	0.01%
unclassified Dyella	21	0.01%
unclassified Jishengella	21	0.01%
Devosia geojensis	21	0.01%
unclassified Frankiaceae	21	0.01%
Mycobacterium barrassiae	21	0.01%
unclassified Oryzihumus	21	0.01%
unclassified Leptospiraceae	21	0.01%
delta proteobacterium	21	0.01%
Paenibacillus alginolyticus	21	0.01%
unclassified Tumebacillus	21	0.01%
Rhizobacter fulvus	21	0.01%
Sphingobium vulgare	21	0.01%
Clostridium maritimum	21	0.01%
Thermoactinomyces vulgaris	21	0.01%
Cellvibrio fibrivorans	21	0.01%
Dyadobacter alkalitolerans	21	0.01%
Rhodococcus opacus	21	0.01%
Tumebacillus ginsengisoli	21	0.01%
unclassified Opitutae	20	0.01%
unclassified Phycicola	20	0.01%
unclassified Pelotomaculum	20	0.01%
unclassified Gaiellales	20	0.01%

Species	Number of reads	Relative abundance
Chitinophaga soli	20	0.01%
unclassified Schlegelella	20	0.01%
unclassified Phycicoccus	20	0.01%
Frigoribacterium faeni	20	0.01%
Bacillus flexus	20	0.01%
unclassified Cryobacterium	20	0.01%
unclassified Vogesella	20	0.01%
unclassified Desulfobulbus	20	0.01%
Hymenobacter soli	20	0.01%
Archangium gephyra	20	0.01%
Phenylobacterium muchangponense	20	0.01%
unclassified Nonomuraea	20	0.01%
unclassified Rhodobacteraceae	20	0.01%
unclassified Stenotrophomonas	20	0.01%
Bacillus humi	20	0.01%
unclassified Allocatelliglobosispora	19	0%
Nocardioides sediminis	19	0%
unclassified Kineosporiaceae	19	0%
unclassified Labilitrichaceae	19	0%
Leptothrix ginsengisoli	19	0%
Actinoplanes abujensis	19	0%
unclassified Litorilinea	19	0%
Brevundimonas diminuta	19	0%
Bacillus lentus	19	0%
Vasilyevaea mishustinii	19	0%
unclassified Skermanella	19	0%
unclassified Ignavibacteriales	19	0%
Brevundimonas denitrificans	19	0%
unclassified Thiobacteraceae	19	0%
unclassified Chloroflexia	19	0%
unclassified Granulicella	19	0%
unclassified Oxalicibacterium	19	0%
proteobacterium enrichment	19	0%
Methylosinus trichosporium	19	0%
unclassified Sporolactobacillaceae	19	0%
Clostridium cellulovorans	19	0%
Neorhizobium galegae	19	0%
unclassified Chloroidium	19	0%
Ochrobactrum intermedium	19	0%
unclassified Oceanospirillales	18	0%
unclassified Klebsormidium	18	0%
Mesorhizobium albiziae	18	0%
unclassified Bacteroides	18	0%
unclassified Chthonomonadales	18	0%
Microbacterium barkeri	18	0%
unclassified Telluria	18	0%
Tumebacillus permanentifrigoris	18	0%
unclassified Pusillimonas	18	0%
Paenibacillus barengoltzii	18	0%
Sphingomonas oryziterrae	18	0%

Species	Number of reads	Relative abundance
Mesorhizobium huakuii	18	0%
Kribbella albertanoniae	18	0%
unclassified Candidatus Protochlamydia	18	0%
Erythromicrobium ramosum	18	0%
Pedobacter insulae	18	0%
unclassified Chlorella	18	0%
marine actinobacterium	18	0%
unclassified Leifsonia	18	0%
Hyphomicrobium zavarzinii	17	0%
unclassified Ralstonia	17	0%
unclassified Vasilyevaea	17	0%
Angustibacter luteus	17	0%
unclassified Afipia	17	0%
Rhizobium daejeonense	17	0%
Micromonospora lupini	17	0%
Clostridium papyrosolvens	17	0%
unclassified Vulgatibacteraceae	17	0%
Mycobacterium sphagni	17	0%
Clostridium perfringens	17	0%
Psychrosinus fermentans	17	0%
Nocardioides ginsengisegetis	17	0%
unclassified Aquamicrobium	17	0%
Dyadobacter hamtensis	17	0%
Shinella kummerowiae	17	0%
unclassified Gloeobacterales	17	0%
unclassified Demequina	17	0%
Mycobacterium madagascariense	17	0%
unclassified Ignavibacteriaceae	17	0%
unclassified Phormidium	16	0%
Variovorax soli	16	0%
unclassified Ktedonobacteria	16	0%
Pseudomonas frederiksbergensis	16	0%
Sporosarcina newyorkensis	16	0%
Agromyces neolithicus	16	0%
Pseudomonas fluorescens	16	0%
unclassified Ustilaginomycetes	16	0%
Mycobacterium murale	16	0%
Cohnella arctica	16	0%
Pedomicrobium australicum	16	0%
Mucilaginibacter calamicampi	16	0%
unclassified Propionicimonas	16	0%
unclassified Thiotrichales	16	0%
unclassified Plesiocystis	16	0%
unclassified Sphaerisporangium	16	0%
Alloactinosynnema album	16	0%
Microbispora rosea	16	0%
Pedobacter alluvionis	16	0%
Bacillus cecembensis	16	0%
Paenibacillus daejeonensis	16	0%
Pusillimonas noertemannii	16	0%

Species	Number of reads	Relative abundance
Blastococcus jejuensis	16	0%
Beijerinckia indica	16	0%
unclassified Elusimicrobium	16	0%
unclassified Hydrogenophilales	16	0%
unclassified Moorella	16	0%
unclassified Denitratisoma	15	0%
Laceyella sacchari	15	0%
Vitellibacter soesokkakensis	15	0%
unclassified Rubritalea	15	0%
Aridibacter famidurans	15	0%
unclassified Hydrogenedentes	15	0%
unclassified Caryophanon	15	0%
unclassified Candidatus Scalindua	15	0%
Mycobacterium insubricum	15	0%
Pedobacter heparinus	15	0%
unclassified Hyphomonas	15	0%
Terrisporobacter glycolicus	15	0%
unclassified Tahibacter	15	0%
Bartramia environmental	15	0%
Shinella granuli	15	0%
unclassified Shimazuella	15	0%
unclassified Tepidimonas	15	0%
unclassified Catelliglobosispora	15	0%
unclassified Methyloceanibacter	15	0%
Clostridium bifermentans	15	0%
unclassified Dehalogenimonas	15	0%
unclassified Carnobacterium	15	0%
Paenisporosarcina macmurdoensis	15	0%
unclassified Pelosinus	14	0%
unclassified Fibrobacterales	14	0%
Microbacterium murale	14	0%
unclassified Erysipelotrichales	14	0%
unclassified Ruminiclostridium	14	0%
Bacillus aminovorans	14	0%
Pedobacter luteus	14	0%
unclassified Scenedesmaceae	14	0%
unclassified Methylococcales	14	0%
Agromyces ulmi	14	0%
unclassified Gloeobacterophycideae	14	0%
Gaiella occulta	14	0%
Flectobacillus lacus	14	0%
unclassified Desulfomonile	14	0%
Mycobacterium celatum	14	0%
Paenisporosarcina quisquiliarum	14	0%
Agrobacterium rhizogenes	14	0%
Mycobacterium smegmatis	14	0%
Agromyces humatus	14	0%
Devosia soli	14	0%
unclassified Roseburia	14	0%
Tomitella biformata	14	0%

Species	Number of reads	Relative abundance
Mycobacterium sediminis	14	0%
Cellvibrio ostraviensis	14	0%
Rummeliibacillus pycnus	14	0%
unclassified Leucobacter	14	0%
unclassified Fusobacteriales	14	0%
Pelomonas saccharophila	14	0%
Microbacterium foliorum	14	0%
Mycobacterium fortuitum	14	0%
unclassified Acidimicrobiia	14	0%
Blastocatella fastidiosa	13	0%
unclassified Cryptosporangiaceae	13	0%
Clostridium populeti	13	0%
unclassified Tetracoccus	13	0%
unclassified Sporotomaculum	13	0%
unclassified Synechococcaceae	13	0%
unclassified Spirilliplanes	13	0%
unclassified Rickettsiaceae	13	0%
Adhaeribacter terreus	13	0%
unclassified Propionibacteriaceae	13	0%
unclassified mitochondria	13	0%
Sphingomonas azotofornans	13	0%
unclassified	13	0%
unclassified Tetrasphaera	13	0%
Sorangium cellulosum	13	0%
Nostoc punctiforme	13	0%
unclassified Syntrophomonas	13	0%
Singulisphaera rosea	13	0%
Bacillus foraminis	13	0%
unclassified Thioalkalivibrio	13	0%
unclassified Christensenellaceae	13	0%
unclassified Sporomusa	13	0%
Massilia plicata	13	0%
unclassified Leptospira	13	0%
Sphingopyxis baekryungensis	13	0%
Rhodococcus erythropolis	13	0%
Clostridium sulfidigenes	13	0%
Promicromonospora sukumoe	13	0%
unclassified Aminobacter	13	0%
Cellvibrio gandavensis	13	0%
unclassified Beijerinckia	13	0%
unclassified Ammoniphilus	13	0%
Nocardioides alpinus	13	0%
Terrimonas lutea	12	0%
Chryseobacterium balustinum	12	0%
unclassified Actinocorallia	12	0%
Sporosarcina saromensis	12	0%
unclassified Oceanobacillus	12	0%
unclassified Winogradskyella	12	0%
unclassified Entotheonellales	12	0%
Virgisporangium ochraceum	12	0%

Species	Number of reads	Relative abundance
unclassified Erythrobacteraceae	12	0%
unclassified Actinoallomurus	12	0%
unclassified Tepidamorphus	12	0%
Flavobacterium aquidurense	12	0%
unclassified Thermaerobacter	12	0%
unclassified Gordonibacter	12	0%
unclassified Nitrosomonas	12	0%
cf. Chryseobacterium	12	0%
Paradevosia shaoguanensis	12	0%
Singulisphaera acidiphila	12	0%
unclassified Thermoactinomyces	12	0%
unclassified Synechococcales	12	0%
unclassified Trebouxiophyceae	12	0%
unclassified Pinaceae	12	0%
unclassified Kurthia	12	0%
unclassified Haslea	12	0%
Afipia genosp.	12	0%
Flavobacterium segetis	12	0%
unclassified Acidocella	11	0%
unclassified Elusimicrobiaceae	11	0%
Sporacetigenium mesophilum	11	0%
Acidobacterium capsulatum	11	0%
unclassified Simplicispira	11	0%
Micromonospora hermanusense	11	0%
Skermanella stibiiresistens	11	0%
Agromyces cerinus	11	0%
unclassified Rhodovulum	11	0%
unclassified Ktedonobacter	11	0%
Amycolatopsis balhimycina	11	0%
Phormidium autumnale	11	0%
unclassified Caldimonas	11	0%
Nocardioides dilutus	11	0%
unclassified Phyllobacterium	11	0%
Achromobacter xylosoxidans	11	0%
unclassified Enhygromyxa	11	0%
Arctic sea	11	0%
unclassified Luteimicrobium	11	0%
Phaeodactylum tricornutum	11	0%
unclassified Methylococcaceae	11	0%
Agrobacterium larrymoorei	11	0%
unclassified Eubacterium	11	0%
Polaromonas aquatica	11	0%
unclassified Dehalobacterium	11	0%
unclassified Ustilaginales	11	0%
Arthrobacter humicola	11	0%
Aeromicrobium erythreum	10	0%
unclassified Candidimonas	10	0%
Cystobacter ferrugineus	10	0%
Solirubrobacter soli	10	0%
unclassified Pigmentiphaga	10	0%

Species	Number of reads	Relative abundance
unclassified Spirochaetaceae	10	0%
Microvirga guangxiensis	10	0%
unclassified Eoetvoesia	10	0%
unclassified Telmatobacter	10	0%
Mucilaginibacter gracilis	10	0%
Dactylosporangium siamense	10	0%
unclassified Bifidobacterium	10	0%
unclassified Methylophilus	10	0%
Kribbella sandramycini	10	0%
Syntrichia environmental	10	0%
unclassified Longispora	10	0%
Pedobacter bauzanensis	10	0%
unclassified Caenimonas	10	0%
unclassified Saccharothrix	10	0%
unclassified Janibacter	10	0%
unclassified Kineococcus	10	0%
Phenylobacterium mobile	10	0%
Mycobacterium bacteremicum	10	0%
unclassified Aliihoeflea	10	0%
Roseomonas aerophila	10	0%
Lysobacter soli	10	0%
unclassified Paenisporosarcina	10	0%
unclassified Euryarchaeota	10	0%
Aetherobacter rufus	10	0%
unclassified Armatimonadales	10	0%
unclassified Lacibacter	10	0%
unclassified Frigoribacterium	10	0%
Cystobacter violaceus	10	0%
Agromyces fucosus	10	0%
unclassified Nitriliruptoridae	10	0%
unclassified Noviherbaspirillum	10	0%
Burkholderia glathei	10	0%
Thermoactinomyces intermedius	9	0%
Mesorhizobium sangaii	9	0%
Duganella phyllosphaerae	9	0%
unclassified Methanomassiliicoccaceae	9	0%
unclassified Carnobacteriaceae	9	0%
unclassified Amycolatopsis	9	0%
Achromobacter spanius	9	0%
Pseudomonas koreensis	9	0%
Dyadobacter ginsengisoli	9	0%
unclassified Fodinicola	9	0%
Pseudonocardia alaniniphila	9	0%
unclassified Uliginosibacterium	9	0%
Eoetvoesia caeni	9	0%
unclassified Sorangium	9	0%
Flavitalea gansuensis	9	0%
Duganella zoogloeoides	9	0%
Nocardioides plantarum	9	0%
Pedobacter ginsengisoli	9	0%

Species	Number of reads	Relative abundance
Actinoplanes liguriensis	9	0%
Bauldia consociata	9	0%
Flavobacterium limicola	9	0%
unclassified Chryseolinea	9	0%
unclassified Corallococcus	9	0%
Catellatospora methionotrophica	9	0%
Actinoallomurus iriomotensis	9	0%
unclassified Sediminibacterium	9	0%
unclassified Oligoflexales	9	0%
unclassified Alkaliphilus	9	0%
unclassified Pseudaminobacter	9	0%
unclassified Chlamydomonadales	9	0%
Sphingobium aromaticiconvertens	9	0%
unclassified Oligoflexus	9	0%
Nocardioides daphniae	9	0%
Brevundimonas basaltis	9	0%
unclassified Chlorophyta	9	0%
Sphingomonas changbaiensis	9	0%
Sphingobacteriaceae str.	9	0%
Nocardia exalbida	8	0%
Cystobacter fuscus	8	0%
unclassified Angustibacter	8	0%
Sporosarcina thermotolerans	8	0%
Pedobacter wanjuense	8	0%
Arenimonas daejeonensis	8	0%
unclassified Clostridiales Family XVII. Incertae Sedis	8	0%
unclassified Dehalobacteriaceae	8	0%
unclassified Parasegetibacter	8	0%
unclassified Sideroxydans	8	0%
unclassified Methanomicrobiaceae	8	0%
Brevibacillus thermoruber	8	0%
Methylobacter marinus	8	0%
Edaphobacter modestum	8	0%
Amycolatopsis bullii	8	0%
Massilia brevitalea	8	0%
unclassified Streptosporangium	8	0%
unclassified Desulfocurvus	8	0%
unclassified Xenophilus	8	0%
Roseomonas lacus	8	0%
unclassified Nocardia	8	0%
Novosphingobium aromaticivorans	8	0%
Methylobacterium soli	8	0%
Paenibacillus catalpae	8	0%
Oceanobacillus chironomi	8	0%
Aeromicrobium fastidiosum	8	0%
Clostridium sartagoforme	8	0%
Hymenobacter norwichensis	8	0%
Actinomycetospora straminea	8	0%
unclassified Chroococcidiopsis	8	0%
Flavisolibacter ginsengiterrae	8	0%

Species	Number of reads	Relative abundance
Clostridium hungatei	8	0%
Pseudomonas borealis	8	0%
Geobacillus thermodenitrificans	8	0%
Geodermatophilus obscurus	8	0%
unclassified Promicromonospora	8	0%
unclassified Sanguibacteraceae	8	0%
unclassified Oscillospira	8	0%
Clostridium magnum	8	0%
Bacillus isronensis	8	0%
unclassified Sphaerotilus	8	0%
Aeromicrobium kwangyangensis	8	0%
Flavobacterium xinjiangense	8	0%
unclassified Labrys	8	0%
unclassified Plantactinospora	8	0%
Nocardioides pyridinolyticus	8	0%
unclassified IIb	8	0%
unclassified Rhodopirellula	8	0%
Intrasporangium mesophilum	8	0%
Rummeliibacillus stabekisii	8	0%
Stenotrophomonas acidaminiphila	8	0%
Arthrobacter kerguelensis	7	0%
unclassified Cellulomonadaceae	7	0%
unclassified Acinetobacter	7	0%
Thermomonas brevis	7	0%
Clostridium vincentii	7	0%
unclassified Cerasicoccales	7	0%
Mucilaginibacter sabulilitoris	7	0%
Brevundimonas aurantiaca	7	0%
Staphylococcus saprophyticus	7	0%
unclassified Chlamydomonadaceae	7	0%
unclassified Thermodesulfovibrionaceae	7	0%
Planctomycete enrichment	7	0%
unclassified Pseudosporangium	7	0%
unclassified Spirochaeta	7	0%
unclassified Diaminobutyricimonas	7	0%
Blastomonas natatoria	7	0%
Prevotella copri	7	0%
unclassified Thermus	7	0%
Carnobacterium viridans	7	0%
Cohnella fontinalis	7	0%
Rhodococcus maanshanensis	7	0%
unclassified Aurantimonas	7	0%
unclassified Nitrosopumilales	7	0%
unclassified Xylophilus	7	0%
unclassified Trichococcus	7	0%
Mycobacterium crocinum	7	0%
unclassified Beutenbergia	7	0%
Pseudomonas lini	7	0%
unclassified Cystobacterineae	7	0%
unclassified Pseudoclavibacter	7	0%

Species	Number of reads	Relative abundance
Polymorphospora rubra	7	0%
Thermobacillus composti	7	0%
unclassified Thermanaerothrix	7	0%
unclassified Rudaea	7	0%
Streptomyces scabrisporus	7	0%
unclassified Symbiobacteriaceae	7	0%
unclassified Pinus	7	0%
unclassified Fulvimarina	7	0%
unclassified Alsobacter	7	0%
unclassified Sinorhizobium	7	0%
unclassified Candidatus Brocadiales	7	0%
Geodermatophilus terrae	7	0%
Streptomyces pulveraceus	7	0%
Paenibacillus algorifonticola	7	0%
Flavobacterium myungsuense	7	0%
unclassified Tomitella	7	0%
unclassified Aquificae	7	0%
unclassified Dehalobacter_Syntrophobotulus	7	0%
Aminobacter niigataensis	7	0%
unclassified Methylophilales	7	0%
Devosia subaequoris	7	0%
unclassified Roseimicrobium	7	0%
Epilithonimonas tenax	7	0%
Bdellovibrio bacteriovorus	7	0%
unclassified Rhodothermaceae	7	0%
Clostridium favososporum	7	0%
unclassified Lutispora	7	0%
unclassified Sterolibacterium	6	0%
unclassified Thermales	6	0%
Flavobacterium glaciei	6	0%
Nocardioides fonticola	6	0%
unclassified Petrimonas	6	0%
Nannocystis pusilla	6	0%
unclassified Chloroplast	6	0%
unclassified Arthrospira	6	0%
Chloroidium saccharophilum	6	0%
Polaromonas vacuolata	6	0%
unclassified Aquimonas	6	0%
Smaragdicoccus niigatensis	6	0%
unclassified Anaerobranca	6	0%
unclassified Reichenowia	6	0%
Leptothrix mobilis	6	0%
Lysobacter niabensis	6	0%
unclassified Thermomicrobiaceae	6	0%
Nitzschia frustulum	6	0%
Ochrobactrum pseudogrignonense	6	0%
unclassified Nocardiopsaceae	6	0%
unclassified Achromobacter	6	0%
unclassified Labilithrix	6	0%
unclassified Tissierellaceae	6	0%

Species	Number of reads	Relative abundance
Clostridium neonatale	6	0%
Streptomyces ahygroscopicus	6	0%
unclassified Halanaerobiales	6	0%
unclassified Phormidiaceae	6	0%
Luteimonas mephitis	6	0%
Mycobacterium tuberculosis	6	0%
Paenibacillus turicensis	6	0%
Reyranella soli	6	0%
unclassified Actinokineospora	6	0%
Shewanella algae	6	0%
Arthrobacter antarcticus	6	0%
Luteibacter rhizovicinus	6	0%
unclassified Camelimonas	6	0%
Alicyclobacillus pomorum	6	0%
unclassified Mycetocola	6	0%
unclassified Sulfuricella	6	0%
Pedobacter kwangyangensis	6	0%
unclassified Thaumarchaeota	6	0%
unclassified Rhabdochlamydiaceae	6	0%
Flavobacterium columnare	6	0%
unclassified Haloplasmatales	6	0%
unclassified Oxalobacter	6	0%
unclassified Estrella	6	0%
unclassified Coriobacteriaceae	6	0%
Physcomitrella patens	6	0%
unclassified Anaerostipes	6	0%
Mycobacterium litorale	6	0%
Rhizobium cellulosilyticum	6	0%
Devosia chinhatensis	6	0%
Microcoleus vaginatus	6	0%
unclassified Aquincola	6	0%
unclassified Kofleria	6	0%
unclassified Geminicoccus	6	0%
Dyadobacter fermentans	6	0%
Brevundimonas staleyi	6	0%
unclassified Brucellaceae	6	0%
Paenibacillus polymyxa	6	0%
Blautia obeum	6	0%
Aminobacter aminovorans	6	0%
unclassified Trueperaceae	6	0%
Microbacterium thalassium	6	0%
Clostridium peptidivorans	6	0%
unclassified Desulfuromonas	6	0%
Paenibacillus prosopidis	6	0%
Ilumatobacter fluminis	6	0%
unclassified Anaerophaga	5	0%
unclassified Lysinimonas	5	0%
Actinoplanes xinjiangensis	5	0%
Sporosarcina aquimarina	5	0%
Brevundimonas mediterranea	5	0%

Species	Number of reads	Relative abundance
Sporocytophaga myxococcoides	5	0%
Nitrospira moscoviensis	5	0%
unclassified Haloplasmataceae	5	0%
unclassified Candidatus Nitrosoarchaeum	5	0%
Dyella ginsengisoli	5	0%
Kineococcus radiotolerans	5	0%
unclassified Lechevalieria	5	0%
Thiobacillus thioparus	5	0%
unclassified Verrucomicrobia	5	0%
Tabrizicola aquatica	5	0%
Mycobacterium neoaurum	5	0%
unclassified Paenirhodobacter	5	0%
unclassified Actinomadura	5	0%
Blautia wexlerae	5	0%
Pedobacter daejeonensis	5	0%
unclassified Starria	5	0%
Aquaspirillum arcticum	5	0%
unclassified Ornithinibacillus	5	0%
unclassified Sandaracinaceae	5	0%
Demequina oxidasica	5	0%
Bacillus pocheonensis	5	0%
Paenibacillus odorifer	5	0%
Devosia neptuniae	5	0%
Sphingomonas panni	5	0%
unclassified Ktedonobacterales	5	0%
unclassified Crenarchaeota	5	0%
Paenibacillus hordei	5	0%
unclassified Brevibacillus	5	0%
Marmoricola korecus	5	0%
unclassified Fimbriimonadia	5	0%
Rudaea cellulosilytica	5	0%
Candidatus Nitrososphaera gargensis	5	0%
unclassified Bryocella	5	0%
Actinomycetales str.	5	0%
unclassified Intrasporangium	5	0%
unclassified Tatlockia	5	0%
Nitrosomonas communis	5	0%
unclassified Aequorivita	5	0%
unclassified Pseudogracilibacillus	5	0%
unclassified Umezawaea	5	0%
unclassified Larkinella	5	0%
Rhodanobacter glycinis	5	0%
Arthrobacter scleromae	5	0%
Flavobacterium soli	5	0%
unclassified Pinidae	5	0%
unclassified Miniimonas	5	0%
Micropruina glycogenica	5	0%
unclassified Collimonas	5	0%
Rhodococcus triatomae	5	0%
unclassified Eukaryota	5	0%

Species	Number of reads	Relative abundance
Microbacterium esteraromaticum	5	0%
Methylocaldum tepidum	5	0%
Achromobacter insuavis	5	0%
Pseudosporangium ferrugineum	5	0%
unclassified Thermopolyspora	5	0%
unclassified Armatimonadia	5	0%
Clostridium cellulolyticum	5	0%
unclassified Virgisporangium	5	0%
Stenotrophomonas geniculata	5	0%
unclassified Negativicutes	5	0%
Pelosinus propionicus	5	0%
Nocardioides szechwanensis	5	0%
unclassified Staphylococcaceae	5	0%
unclassified Thermoflexia	5	0%
Massilia niabensis	5	0%
unclassified Stigmatella	5	0%
Mycobacterium arupense	5	0%
Hydrogenophaga defluvii	5	0%
Clostridium aldrichii	5	0%
unclassified Paucimonas	5	0%
unclassified Oerskovia	5	0%
Raoultella terrigena	5	0%
unclassified Methylacidiphilae	5	0%
Planifilum yunnanense	5	0%
Sphingomonas astaxanthinifaciens	5	0%
Rhodanobacter terrae	5	0%
unclassified Thiobacterales	5	0%
Dyadobacter soli	5	0%
unclassified Pelomonas	5	0%
Cupriavidus basilensis	5	0%
unclassified Thermoanaerobacter	5	0%
unclassified Fusobacteriia	5	0%
Brevundimonas subvibrioides	5	0%
Streptomyces sodiiphilus	5	0%
unclassified Thermomonosporaceae	5	0%
Nitrospira marina	5	0%
unclassified Thalassiosira	5	0%
unclassified Thiorhodovibrio	5	0%
unclassified Roseospira	5	0%
Modestobacter versicolor	5	0%
Kinneretia asaccharophila	5	0%
Microlunatus ginsengisoli	5	0%
unclassified Selenomonadales	5	0%
unclassified Ideonella	4	0%
Actinomadura vinacea	4	0%
Polaromonas ginsengisoli	4	0%
unclassified Acidipila	4	0%
unclassified Enterobacteriales	4	0%
Labrys methylaminiphilus	4	0%
unclassified Sulfuritalea	4	0%

Species	Number of reads	Relative abundance
Sporosarcina ureae	4	0%
Arthrospira fusiformis	4	0%
unclassified Cymatopleura	4	0%
Oceanobacillus oncorhynchi	4	0%
Bacillus mycoides	4	0%
unclassified Haloactinobacterium	4	0%
unclassified Cenarchaeaceae	4	0%
unclassified Mogibacteriaceae	4	0%
Flavobacterium hibernum	4	0%
Rhodobacter ovatus	4	0%
Polaromonas jejuensis	4	0%
unclassified Desulfoglaeba	4	0%
Paenibacillus curdlanolyticus	4	0%
Lentzea violacea	4	0%
Hymenobacter actinosclerus	4	0%
Nocardia tenerifensis	4	0%
Massilia dura	4	0%
Reyranella massiliensis	4	0%
unclassified Thermoplasmatales	4	0%
unclassified Methylovorus	4	0%
Auxenochlorella protothecoides	4	0%
unclassified Aquaspirillum	4	0%
unclassified Woodsholea	4	0%
Modestobacter marinus	4	0%
Jatrophihabitans endophyticus	4	0%
Solibacillus silvestris	4	0%
Paenibacillus glacialis	4	0%
unclassified Edaphobacter	4	0%
Parapedobacter pyrenivorans	4	0%
Streptomyces thermovulgaris	4	0%
unclassified Marinilabiliaceae	4	0%
Pseudomonas azotoformans	4	0%
Halomonas axialensis	4	0%
unclassified Nostoc	4	0%
Sphingomonas alpina	4	0%
Acidovorax radicis	4	0%
unclassified Kopriimonadales	4	0%
Agromyces salentinus	4	0%
Kribbella antibiotica	4	0%
Sphingomonas xinjiangensis	4	0%
Microbacterium takaoensis	4	0%
Microbacterium profundi	4	0%
Acinetobacter lwoffii	4	0%
Xiangella phaseoli	4	0%
unclassified Bifidobacteriaceae	4	0%
Bacillus graminis	4	0%
unclassified Mollicutes	4	0%
Paenibacillus aestuarii	4	0%
unclassified Lachnospira	4	0%
unclassified Thermincola	4	0%

Species	Number of reads	Relative abundance
Nocardioides caeni	4	0%
Geobacter bremensis	4	0%
Anaerosalibacter bizertensis	4	0%
Anaerostipes hadrus	4	0%
unclassified Nitrosopumilaceae	4	0%
unclassified Solitalea	4	0%
unclassified Clostridiales Family XI. Incertae Sedis	4	0%
unclassified Gelidibacter	4	0%
Paenibacillus taohuashanense	4	0%
Halomonas phoceae	4	0%
Kribbella flavida	4	0%
unclassified Limnohabitans	4	0%
Microbacterium phyllosphaerae	4	0%
Paenibacillus taichungensis	4	0%
unclassified Candidatus Alysiosphaera	4	0%
Nakamurella lactea	4	0%
unclassified Pseudoduganella	4	0%
Williamsia faeni	4	0%
Mycobacterium haemophilum	4	0%
unclassified Asteroleplasma	4	0%
Streptomyces prasinopilosus	4	0%
unclassified Pezizomycetes	4	0%
Devosia terrae	4	0%
Blastococcus saxobsidens	4	0%
unclassified Anaeroplasmatales	4	0%
unclassified Chromulinales	4	0%
Micromonospora pattaloongensis	4	0%
Rhodococcus phenolicus	4	0%
crenarchaeote enrichment	4	0%
Herpetosiphon aurantiacus	4	0%
Saccharothrix longispora	4	0%
unclassified Oscillochloris	4	0%
Paenibacillus ginsengisoli	4	0%
Rathayibacter caricis	4	0%
Cylindrospermum muscicola	4	0%
Mesorhizobium alhagi	4	0%
Beutenbergia cavernae	4	0%
Telluria mixta	4	0%
Gordonia malaquae	4	0%
unclassified Leptospirales	4	0%
unclassified Pseudochrobactrum	4	0%
Streptomyces kagoshimanus	4	0%
unclassified Vulgatibacter	4	0%
unclassified Jatrophihabitans	4	0%
Thermopolyspora flexuosa	4	0%
Sphingopyxis litoris	4	0%
unclassified Actinosynnemataceae	4	0%
unclassified Rickettsiella	4	0%
Bifidobacterium adolescentis	4	0%
Plantactinospora endophytica	4	0%

Species	Number of reads	Relative abundance
Allocatelliglobosispora scoriae	4	0%
unclassified Actinomycetospora	4	0%
Nocardioides tritolerans	4	0%
Phenylobacterium koreense	4	0%
Paenibacillus agaridevorans	4	0%
Candidatus Devosia	4	0%
unclassified Jeotgalibacillus	4	0%
Streptomyces mashuensis	4	0%
Chryseobacterium indologenes	4	0%
Segetibacter koreensis	4	0%
unclassified Acidithiobacillales	4	0%
Acetobacterium bakii	4	0%
Corallococcus macrosporus	4	0%
unclassified Rhizocola	4	0%
unclassified Vitellibacter	4	0%
unclassified Thermanaeromonas	4	0%
unclassified Anaerobacterium	4	0%
Nocardioides caricicola	4	0%
Mucilaginibacter rigui	4	0%
unclassified Methylocella	4	0%
Mucilaginibacter ximonensis	4	0%
Tsukamurella strandjordii	4	0%
unclassified Pimelobacter	4	0%
Coprococcus eutactus	4	0%
Methanosarcina mazei	3	0%
Micromonospora siamensis	3	0%
Burkholderia terrestris	3	0%
Streptomyces lilacinus	3	0%
Tahibacter aquaticus	3	0%
Enterobacter aerogenes	3	0%
unclassified Chlorophyceae	3	0%
Sphingomonas canadensis	3	0%
unclassified Schlesneria	3	0%
Proteiniclasticum ruminis	3	0%
Achromobacter pulmonis	3	0%
Actinoallomurus spadix	3	0%
unclassified Ardenscatenaceae	3	0%
Mesorhizobium camelthorni	3	0%
Leucobacter salsicius	3	0%
Pseudomonas punonensis	3	0%
unclassified Fluoribacter	3	0%
Taibaiella koreensis	3	0%
Pedobacter terrae	3	0%
Chryseobacterium indoltheticum	3	0%
Vasilyevaea enhydra	3	0%
Mycobacterium brisbanense	3	0%
unclassified Bryopsida	3	0%
Nonomuraea fastidiosa	3	0%
Paenibacillus cookii	3	0%
unclassified Pelagibacterium	3	0%

Species	Number of reads	Relative abundance
unclassified Azorhizobium	3	0%
unclassified Ulvales	3	0%
Thermomonospora chromogena	3	0%
unclassified Bacteroidetes	3	0%
Arthrobacter sulfonivorans	3	0%
unclassified Viridibacillus	3	0%
Paenibacillus antarcticus	3	0%
Pseudonocardia endophytica	3	0%
Saccharothrix texasensis	3	0%
unclassified Brocadiales	3	0%
unclassified Aquisphaera	3	0%
Nesterenkonia halotolerans	3	0%
unclassified Alistipes	3	0%
Bacillus acidicola	3	0%
unclassified Sufflavibacter	3	0%
Asanoa iriomotensis	3	0%
Haslea ostrearia	3	0%
unclassified Syntrophobacter	3	0%
Aureimonas phyllosphaerae	3	0%
Clostridium frigidicarnis	3	0%
unclassified Oligoflexia	3	0%
Massilia niastensis	3	0%
unclassified Cellulosimicrobium	3	0%
Ureibacillus suwonensis	3	0%
unclassified Rhodobacter	3	0%
Dyadobacter beijingensis	3	0%
unclassified Fusicatenibacter	3	0%
unclassified Symbiobacterium	3	0%
Massilia aerilata	3	0%
Thermobispora bispora	3	0%
unclassified Maricaulis	3	0%
Nocardioides exalbidus	3	0%
unclassified Auxenochlorella	3	0%
Methanosarcina barkeri	3	0%
unclassified Asanoa	3	0%
Starkeya novella	3	0%
Acidovorax avenae	3	0%
Porphyrobacter tepidarius	3	0%
Sphingomonas humi	3	0%
unclassified Paracraurococcus	3	0%
unclassified Collinsella	3	0%
Kaistibacter ginsenosidimutans	3	0%
unclassified Ardenscatenales	3	0%
unclassified Acholeplasmatales	3	0%
unclassified Synechococcus	3	0%
Mycobacterium chlorophenolicum	3	0%
Cohnella yongneupensis	3	0%
unclassified Dorea	3	0%
Agromyces atrinae	3	0%
Fictibacillus phosphorivorans	3	0%

Species	Number of reads	Relative abundance
unclassified Kutzneria	3	0%
unclassified Balneimonas	3	0%
Clostridium tunisiense	3	0%
Bacillus koreensis	3	0%
Bacillus pumilus	3	0%
Paenibacillus ginsengihumi	3	0%
Akkermansia muciniphila	3	0%
Agrobacterium tumefaciens	3	0%
unclassified Glycomycetaceae	3	0%
Nocardioidaceae str.	3	0%
unclassified Humibacillus	3	0%
unclassified Ancylobacter	3	0%
Arcticibacter svalbardensis	3	0%
unclassified Methylosarcina	3	0%
Pedomicrobium manganicum	3	0%
Leucobacter aerolatus	3	0%
Chryseobacterium aquaticum	3	0%
Microbispora corallina	3	0%
Burkholderia phytofirmans	3	0%
unclassified Leptospirae	3	0%
Paracoccus aminovorans	3	0%
Caldibacillus debilis	3	0%
unclassified Ktedonobacteraceae	3	0%
unclassified Nitratireductor	3	0%
Roseomonas aquatica	3	0%
unclassified Methylobacillus	3	0%
unclassified Sporacetigenium	3	0%
Sphingobium czechense	3	0%
Salinispora pacifica	3	0%
Phyllobacterium ifriqiyense	3	0%
unclassified Chromatiaceae	3	0%
Paenibacillus terrigena	3	0%
unclassified Vampirovibrio	3	0%
Bacillus cereus	3	0%
Clostridium beijerinckii	3	0%
Sphingopyxis rigui	3	0%
unclassified Algoriphagus	3	0%
Nonomuraea harbinensis	3	0%
Cohnella luojiensis	3	0%
Dietzia lutea	3	0%
Pseudomonas jessenii	3	0%
Phycicoccus aerophilus	3	0%
unclassified Sphingoterrabacterium	3	0%
Streptomyces olivoverticillatus	3	0%
Alkalibaculum bacchi	3	0%
Flavisolibacter ginsengisoli	3	0%
Nostoc commune	3	0%
Arthrobacter agilis	3	0%
unclassified Albidovulum	3	0%
Massilia varians	3	0%

Species	Number of reads	Relative abundance
unclassified Schumannella	3	0%
unclassified Anaerosporobacter	3	0%
Bacillus azotoformans	3	0%
Ammoniphilus oxalivorans	3	0%
Hyphomicrobium methylovorum	2	0%
Lechevalieria aerocolonigenes	2	0%
unclassified Methylothermus	2	0%
Kurthia gibsonii	2	0%
Pseudomonas oryzihabitans	2	0%
unclassified Methylocapsa	2	0%
Janibacter limosus	2	0%
Devosia riboflavina	2	0%
Pseudoxanthomonas wuyuanensis	2	0%
Mycobacterium szulgai	2	0%
Clostridium botulinum	2	0%
Angustibacter peucedani	2	0%
Marinobacter bryozoorum	2	0%
Bacillus galactosidilyticus	2	0%
Clostridium mangenotii	2	0%
Labilithrix luteola	2	0%
Noviherbaspirillum soli	2	0%
Actinocorallia libanotica	2	0%
Cellulomonas hominis	2	0%
Sporosarcina psychrophila	2	0%
Pedobacter kribbensis	2	0%
unclassified Jahnella	2	0%
Bacillus persicus	2	0%
Sporosarcina ginsengi	2	0%
Dongia mobilis	2	0%
unclassified Hyalangium	2	0%
Pseudonocardia xinjiangensis	2	0%
unclassified Pottiaceae	2	0%
Microbacterium binotii	2	0%
Pseudonocardia petroleophila	2	0%
Luteimonas composti	2	0%
unclassified Methanosarcina	2	0%
unclassified Reyranella	2	0%
Promicromonospora aerolata	2	0%
Paenibacillus pocheonensis	2	0%
unclassified Laceyella	2	0%
unclassified Desulfotomaculum	2	0%
Caryophanon latum	2	0%
unclassified Nitrosococcus	2	0%
Herminiimonas arsenicoxydans	2	0%
Streptomyces prasinus	2	0%
Streptomyces melanosporofaciens	2	0%
unclassified Nostocaceae	2	0%
Pseudorhodoferax aquiterrae	2	0%
Paracoccus marcusii	2	0%
unclassified Salana	2	0%

Species	Number of reads	Relative abundance
Hymenobacter antarcticus	2	0%
unclassified Sphingomicrobium	2	0%
Catenulispora cavernae	2	0%
Naxibacter indica	2	0%
unclassified Microcella	2	0%
unclassified Nostocoida type II	2	0%
Arthrobacter mysorens	2	0%
Serratia plymuthica	2	0%
unclassified Promicromonosporaceae	2	0%
Solitalea canadensis	2	0%
unclassified Lactobacillaceae	2	0%
unclassified Sandaracinus	2	0%
unclassified Terrisporobacter	2	0%
unclassified Nitrobacter	2	0%
Ureibacillus thermosphaericus	2	0%
Amycolatopsis xylanica	2	0%
unclassified Candidatus Nardonella	2	0%
Roseomonas frigidaquae	2	0%
Ochrobactrum grignonense	2	0%
Ruminococcus gnavus	2	0%
unclassified Tsukamurella	2	0%
Sphingomonas yunnanensis	2	0%
Micromonospora chaiyaphumensis	2	0%
Bacillus horneckiae	2	0%
Cylindrospermum alatosporum	2	0%
Pseudomonas rhizosphaerae	2	0%
unclassified Georgenia	2	0%
unclassified Deinococci	2	0%
Pseudomonas fragi	2	0%
unclassified Leptolyngbya	2	0%
Cohnella suwonensis	2	0%
unclassified Pseudoruegeria	2	0%
Phascolarctobacterium succinatutens	2	0%
unclassified Terracoccus	2	0%
Agrococcus jenensis	2	0%
Methylobacterium extorquens	2	0%
Candidatus Koribacter versatilis	2	0%
Clostridium cellulosi	2	0%
unclassified Ruaniaceae	2	0%
Flavobacterium macrobrachii	2	0%
Phenylobacterium falsum	2	0%
unclassified Colwelliaceae	2	0%
Rhodomicrobium udaipurense	2	0%
unclassified Defluviicoccus	2	0%
Mycobacterium chelonae	2	0%
unclassified Luteibacter	2	0%
Mycobacterium minnesotense	2	0%
unclassified Desulfocapsa	2	0%
Flavihumibacter petaseus	2	0%
Phormidium murrayii	2	0%

Species	Number of reads	Relative abundance
unclassified Nitrososphaeraceae	2	0%
Pullulanibacillus naganoensis	2	0%
Nocardioides hwasunensis	2	0%
Arthrobacter rhombi	2	0%
Angustibacter aerolatus	2	0%
Ramlibacter henchirensis	2	0%
unclassified Bifidobacteriales	2	0%
unclassified Epulopiscium	2	0%
Paenibacillus graminis	2	0%
unclassified Herbiconiux	2	0%
Methylotenera versatilis	2	0%
Geobacter bemidjiensis	2	0%
unclassified Parvibaculum	2	0%
unclassified Pseudorhodoferax	2	0%
unclassified Sulfurisoma	2	0%
Flavobacterium limnosediminis	2	0%
unclassified Criblamydia	2	0%
Mycobacterium confluentis	2	0%
Aeromonas hydrophila	2	0%
Paenibacillus lautus	2	0%
Hymenobacter algoricola	2	0%
unclassified Wandonia	2	0%
Paenibacillus illinoisensis	2	0%
Caenimonas terrae	2	0%
Arenimonas malthae	2	0%
Bdellovibrio exovorus	2	0%
Streptomyces sulfonofaciens	2	0%
unclassified Gloeobacteraceae	2	0%
Parasegetibacter luojiensis	2	0%
unclassified Naviculales	2	0%
denitrifying Fe-oxidizing	2	0%
Amaricoccus macauensis	2	0%
Mycobacterium moriokaense	2	0%
Paenochrobactrum gallinarii	2	0%
unclassified Cryptosporangium	2	0%
Catenulispora graminis	2	0%
unclassified Dethiobacter	2	0%
unclassified Planifilum	2	0%
Bacillus thermolactis	2	0%
Saccharomonospora viridis	2	0%
Mucilaginibacter dorajii	2	0%
Brevibacillus brevis	2	0%
unclassified Chlamydiaceae	2	0%
unclassified Melioribacteraceae	2	0%
unclassified Defluviimonas	2	0%
unclassified Ardenscatena	2	0%
Hephaestia caeni	2	0%
unclassified Bogoriellaceae	2	0%
Burkholderia bryophila	2	0%
unclassified Comamonas	2	0%

Species	Number of reads	Relative abundance
Pedobacter arcticus	2	0%
unclassified Catenulispora	2	0%
Burkholderia hospita	2	0%
unclassified Parvarchaeota	2	0%
Actinomadura scrupuli	2	0%
Halomonas meridiana	2	0%
Alicyclobacillus disulfidooxidans	2	0%
unclassified Rudanella	2	0%
Pseudomonas lutea	2	0%
Starria zimbabweensis	2	0%
Legionella quateirensis	2	0%
unclassified Vibrio	2	0%
Pseudomonas stutzeri	2	0%
unclassified Tuberaceae	2	0%
Modestobacter multiseptatus	2	0%
Bacillus marisflavi	2	0%
Chryseobacterium haifense	2	0%
Prosthecobacter debontii	2	0%
Saccharothrix espanaensis	2	0%
unclassified Candidatus Amoebophilus	2	0%
Brevibacillus limnophilus	2	0%
Stella humosa	2	0%
Corynebacterium stationis	2	0%
unclassified Rummeliibacillus	2	0%
unclassified Flectobacillus	2	0%
Lysobacter capsici	2	0%
Microvirga flocculans	2	0%
unclassified Sneathiellales	2	0%
unclassified Nostocophycideae	2	0%
Streptomyces albus	2	0%
unclassified Parastreptomyces	2	0%
humic substances	2	0%
Arthrobacter tumbae	2	0%
Pedobacter ruber	2	0%
unclassified Cryptanaerobacter	2	0%
Hymenobacter perfusus	2	0%
unclassified Curvibacter	2	0%
unclassified Ethanoligenens	2	0%
unclassified Epilithonimonas	2	0%
unclassified Methanomicrobiales	2	0%
unclassified Erysipelotrichi	2	0%
unclassified Bartramiaceae	2	0%
unclassified Fibrobacteraceae	2	0%
unclassified Spirochaetales	2	0%
Clostridium chartatabidum	2	0%
unclassified Chloracidobacteraceae	2	0%
Pantoea vagans	2	0%
unclassified HTCC	2	0%
unclassified Carboxydothermus	2	0%
unclassified Phaeodactylaceae	2	0%

Species	Number of reads	Relative abundance
Rhodococcus corynebacterioides	2	0%
Polaromonas rhizosphaerae	2	0%
Ruminococcus bicirculans	2	0%
unclassified Chromobacteriaceae	2	0%
Sphingobacterium multivorum	2	0%
Burkholderia soli	2	0%
unclassified Erysipelotrichaceae	2	0%
unclassified Anabaena	2	0%
unclassified Naviculaceae	2	0%
unclassified Desmonostoc	2	0%
unclassified Micropruina	2	0%
Coprococcus catus	2	0%
Hydrogenophaga palleronii	2	0%
unclassified Candidatus Brocadia	2	0%
unclassified Smaragdicoccus	2	0%
Nocardioides lianchengensis	2	0%
Novosphingobium barchaimii	2	0%
unclassified Xiangella	2	0%
unclassified Stackebrandtia	2	0%
Bacteroides dorei	2	0%
Actinocorallia aurantiaca	2	0%
unclassified Herpetosiphonaceae	2	0%
unclassified Alkalibaculum	2	0%
Patulibacter ginsengiterrae	2	0%
unclassified Euzebya	2	0%
Sphingopyxis taejonensis	2	0%
unclassified Desulfobulbaceae	2	0%
unclassified Starkeya	2	0%
Bacillus safensis	2	0%
unclassified Ignavibacterium	2	0%
Legionella-like amoebal	2	0%
Paenibacillus mendelii	2	0%
unclassified Serratia	2	0%
unclassified Dermabacteraceae	2	0%
unclassified Ottowia	2	0%
unclassified Mitsuaria	2	0%
unclassified Pseudanabaenaceae	2	0%
Micromonospora purpureochromogenes	2	0%
Microbacterium lemovicicum	2	0%
Eubacterium eligens	2	0%
unclassified Roseococcus	2	0%
Novosphingobium lentum	2	0%
unclassified Thermacetogenium	2	0%
unclassified Bartonellaceae	2	0%
unclassified Fusibacter	2	0%
unclassified Cylindrospermum	2	0%
unclassified FBP	2	0%
Pilimelia terevasa	2	0%
Propionivibrio militaris	1	0%
Promicromonospora cymbopogonis	1	0%

Species	Number of reads	Relative abundance
Nannocystis aggregans	1	0%
Streptomyces clavuligerus	1	0%
unclassified Acidithiobacillaceae	1	0%
Clostridium aminobutyricum	1	0%
Algoriphagus terrigena	1	0%
Arthrobacter arilaitensis	1	0%
Kaistia hirudinis	1	0%
unclassified Stramenopiles	1	0%
Massilia suwonensis	1	0%
unclassified Procabacteriales	1	0%
Bifidobacterium longum	1	0%
Gemmata-like str.	1	0%
Sphingobacterium nematocida	1	0%
Simplicispira psychrophila	1	0%
Geobacter psychrophilus	1	0%
Cupriavidus campinensis	1	0%
Bacillus idriensis	1	0%
Actinomycetales bacteriuum	1	0%
Streptomyces diastaticus	1	0%
Pedobacter jejuensis	1	0%
Streptosporangium vulgare	1	0%
Tissierella creatinini	1	0%
Agaricicola taiwanensis	1	0%
Sphingomonas indica	1	0%
Blautia faecis	1	0%
unclassified Cerasibacillus	1	0%
Curtobacterium flaccumfaciens	1	0%
Paenibacillus pinisoli	1	0%
unclassified Rhodopila	1	0%
Methylobacterium organophilum	1	0%
Saccharomonospora glauca	1	0%
Aurantimonas coralicida	1	0%
Nostoc ellipsosporum	1	0%
Hymenobacter rigui	1	0%
Pseudolabrys taiwanensis	1	0%
Azoarcus toluclasticus	1	0%
Microbacterium aoyamense	1	0%
Paenibacillus shirakamiensis	1	0%
Paenibacillus macerans	1	0%
Sphingomonas mali	1	0%
unclassified Euglenales	1	0%
Mesorhizobium thiogangeticum	1	0%
unclassified Methanoculleus	1	0%
Aeromicrobium halocynthiae	1	0%
Nocardioides marinisabuli	1	0%
Herminiimonas contaminans	1	0%
unclassified Thermobacillus	1	0%
Phycicoccus dokdonensis	1	0%
Nostoc insulare	1	0%
Roseimicrobium gellanilyticum	1	0%

Species	Number of reads	Relative abundance
Jishengella endophytica	1	0%
Pseudomonas anguilliseptica	1	0%
Eubacterium biforme	1	0%
Mycobacterium intermedium	1	0%
Streptomyces omiyaensis	1	0%
Nostocaceae cyanobacterium	1	0%
Streptomyces glaucescens	1	0%
Aeromicrobium flavum	1	0%
Brevundimonas variabilis	1	0%
Bacillus kokeshiiformis	1	0%
Kribbella alba	1	0%
unclassified Bacteroidetes Order II. Incertae sedis	1	0%
unclassified Micrococcus	1	0%
Aquamicrobium ahrensii	1	0%
Mycobacterium chubuense	1	0%
Thiobacillus aquaesulis	1	0%
Phaeospirillum fulvum	1	0%
unclassified Lactobacillus	1	0%
Marmoricola aurantiacus	1	0%
unclassified Scytonemataceae	1	0%
Emticicia ginsengisoli	1	0%
unclassified Thermomicrobiales	1	0%
unclassified Gemmobacter	1	0%
Rhodococcus jostii	1	0%
Dactylosporangium tropicum	1	0%
Nocardia ignorata	1	0%
unclassified Deinococcus	1	0%
unclassified Gluconacetobacter	1	0%
Arthrobacter alpinus	1	0%
Promicromonospora citrea	1	0%
unclassified Pleurocapsales	1	0%
Pseudoxanthomonas sacheonensis	1	0%
unclassified Beutenbergiaceae	1	0%
Amycolatopsis equina	1	0%
Pedobacter koreensis	1	0%
Leucobacter albus	1	0%
unclassified Methylovirgula	1	0%
unclassified Isoptericola	1	0%
unclassified Lutibacterium	1	0%
Sphingobium lactosutens	1	0%
Nocardia carnea	1	0%
Aureimonas rubiginis	1	0%
unclassified Coscinodiscophyceae	1	0%
Labrys monachus	1	0%
Flavobacterium fluvii	1	0%
Ruminococcus faecis	1	0%
Micromonospora peucetia	1	0%
Pantoea brenneri	1	0%
Flavobacterium terrigena	1	0%
Sphingobium suberifaciens	1	0%

Species	Number of reads	Relative abundance
Enhydrobacter aerosaccus	1	0%
Actinoplanes deccanensis	1	0%
unclassified Lelliottia	1	0%
Amaricoccus tamworthensis	1	0%
Bacillus nitritophilus	1	0%
unclassified Marinicellales	1	0%
Aneurinibacillus danicus	1	0%
Chryseobacterium lathyri	1	0%
Dolichospermum affine	1	0%
unclassified Spirochaetes	1	0%
Bacillus selenatarsenatis	1	0%
unclassified Neisseriales	1	0%
Alsobacter metallidurans	1	0%
Sphingobium chlorophenolicum	1	0%
Luteolibacter yonseiensis	1	0%
unclassified Piscinibacter	1	0%
Bacillus licheniformis	1	0%
Flavobacterium sinopsychrotolerans	1	0%
Bacillus clausii	1	0%
Alkaliphilus halophilus	1	0%
Paenibacillus glycanilyticus	1	0%
Sphingopyxis flavimaris	1	0%
unclassified Pseudohaliea	1	0%
Salana multivorans	1	0%
Rhizobium giardinii	1	0%
Rhodococcus koreensis	1	0%
Curtobacterium ammoniigenes	1	0%
unclassified Catenulisporaceae	1	0%
Paenibacillus telluris	1	0%
Ancylobacter dichloromethanicus	1	0%
unclassified Barnesiellaceae	1	0%
Acinetobacter johnsonii	1	0%
Porphyrobacter cryptus	1	0%
unclassified Pullulanibacillus	1	0%
Astrosporangium hypotensionis	1	0%
Stenotrophomonas humi	1	0%
Clostridium sufflavum	1	0%
Lactobacillus rogosae	1	0%
Aquipuribacter hungaricus	1	0%
Koliella planctonica	1	0%
unclassified Actinosynnema	1	0%
unclassified Pseudobacteroides	1	0%
unclassified Turicibacteraceae	1	0%
Microbacterium trichothecenolyticum	1	0%
Streptomyces erringtonii	1	0%
Nocardia testacea	1	0%
Tetrasphaera elongata	1	0%
unclassified Marinifilum	1	0%
Dyella kyungheensis	1	0%
Cupriavidus gilardii	1	0%

Species	Number of reads	Relative abundance
unclassified Methylophaga	1	0%
unclassified Francisellaceae	1	0%
unclassified Candidatus Entotheonella	1	0%
Nonomuraea jabiensis	1	0%
Pseudoxanthomonas mexicana	1	0%
Paracoccus alcaliphilus	1	0%
Oceanobacillus profundus	1	0%
Ralstonia solanacearum	1	0%
unclassified Lysinimicrobium	1	0%
Brevibacillus reuszeri	1	0%
Bacillus acidiceler	1	0%
Flavobacterium hauense	1	0%
Melghirimyces thermohalophilus	1	0%
Brevundimonas alba	1	0%
unclassified Belnapia	1	0%
unclassified Jiangellaceae	1	0%
unclassified Neochlamydia	1	0%
unclassified Eustigmatales	1	0%
unclassified Helicobacteraceae	1	0%
Aureimonas jatrophae	1	0%
unclassified Propioniferax	1	0%
Citrobacter youngae	1	0%
Bosea lathyri	1	0%
Verrucomicrobium spinosum	1	0%
unclassified Fictibacillus	1	0%
unclassified Thermogemmatisporales	1	0%
unclassified Crenothrix	1	0%
Brevibacillus ginsengisoli	1	0%
unclassified Tabrizicola	1	0%
unclassified Ilyobacter	1	0%
Ochrobactrum anthropi	1	0%
Cohnella soli	1	0%
unclassified Bryobacteraceae	1	0%
Sphingobium yanoikuyae	1	0%
unclassified Parapedobacter	1	0%
Streptomyces ferralitis	1	0%
unclassified Hephaestia	1	0%
unclassified Barnesiella	1	0%
unclassified Robinsoniella	1	0%
unclassified Moheibacter	1	0%
unclassified Delftia	1	0%
anaerobic thermophile	1	0%
Pseudomonas synxantha	1	0%
Atopostipes suicloacalis	1	0%
Granulicella arctica	1	0%
Brevundimonas kwangchunensis	1	0%
Mesorhizobium abyssinicae	1	0%
unclassified Yersinia	1	0%
Pedobacter kyungheensis	1	0%
unclassified Haemophilus	1	0%

Species	Number of reads	Relative abundance
Pseudomonas migulae	1	0%
unclassified Coriobacteriales	1	0%
Paenibacillus anaericanus	1	0%
Brachybacterium conglomeratum	1	0%
unclassified Prototheca	1	0%
unclassified Alloactinosynnema	1	0%
unclassified Rubellimicrobium	1	0%
Lysobacter niastensis	1	0%
unclassified Monodopsidaceae	1	0%
Cytophaga aurantiaca	1	0%
unclassified Moraxellaceae	1	0%
unclassified Tsukamurellaceae	1	0%
Hydrogenophaga taeniospiralis	1	0%
Aquamicrobium aerolatum	1	0%
Bacillus neizhouensis	1	0%
planctomycete str.	1	0%
unclassified Demequinaceae	1	0%
unclassified Helicobacter	1	0%
Tsukamurella spumae	1	0%
Streptomyces sedi	1	0%
Clostridium jejuense	1	0%
Pelomonas puraquae	1	0%
Bacillus hunanensis	1	0%
unclassified Enterobacter	1	0%
Cupriavidus necator	1	0%
Stenotrophomonas maltophilia	1	0%
Agrococcus jejuensis	1	0%
Flavobacterium subsaxonicum	1	0%
Hyphomicrobium denitrificans	1	0%
Vampirovibrio chlorellavorus	1	0%
unclassified Peptostreptococcaceae	1	0%
Virgibacillus halotolerans	1	0%
Chryseobacterium piscicola	1	0%
Streptomyces violaceusniger	1	0%
unclassified Natranaerobiales	1	0%
Saccharothrix xinjiangensis	1	0%
unclassified Tychonema	1	0%
Niastella koreensis	1	0%
Streptomyces sioyaensis	1	0%
unclassified Saccharospirillaceae	1	0%
Chitinophaga arvensicola	1	0%
unclassified Citrobacter	1	0%
Streptomyces thermocarboxydus	1	0%
unclassified Nautiliales	1	0%
unclassified Phaeophyceae	1	0%
unclassified Rickettsia	1	0%
Rubellimicrobium aerolatum	1	0%
Clostridium algidixylanolyticum	1	0%
unclassified Methylocaldum	1	0%
Cryobacterium psychrotolerans	1	0%

Species	Number of reads	Relative abundance
Streptomyces tempisquensis	1	0%
Halomonas venusta	1	0%
unclassified Acidithiobacillus	1	0%
unclassified Prevotella	1	0%
Mycobacterium fluoranthenivorans	1	0%
Glycomyces illinoisensis	1	0%
Curtobacterium plantarum	1	0%
unclassified Proteiniborus	1	0%
Nocardioides hankookensis	1	0%
Sugarcane phytoplasma	1	0%
unclassified Nitzschia	1	0%
Paenibacillus marinisediminis	1	0%
unclassified Pantoea	1	0%
Paenibacillus borealis	1	0%
Promicromonospora umidemergens	1	0%
unclassified Saccharomonospora	1	0%
unclassified Prochlorococcus	1	0%
Comamonas jiangduensis	1	0%
unclassified Cerasicoccaceae	1	0%
Hymenobacter psychrophilus	1	0%
Lactuca sativa	1	0%
unclassified Erysipelotrichia	1	0%
Bacillus trypoxylicola	1	0%
Rhodoferax antarcticus	1	0%
Paucisalibacillus globulus	1	0%
unclassified Candidatus Phytoplasma	1	0%
Nocardioides ginsengagri	1	0%
unclassified Coriobacteriia	1	0%
unclassified Procabacteriaceae	1	0%
unclassified Roseateles	1	0%
Microvirga zambiensis	1	0%
unclassified Actinoalloteichus	1	0%
Nocardioides panacisoli	1	0%
Streptosporangium carneum	1	0%
Sporosarcina pasteurii	1	0%
Lelliottia amnigena	1	0%
Alistipes putredinis	1	0%
Oxalicibacterium solurbis	1	0%
unclassified Puniceicoccales	1	0%
unclassified Parasporobacterium	1	0%
Clostridium senegalense	1	0%
Clostridium tertium	1	0%
unclassified Dicranidae	1	0%
Fusicatenibacter saccharivorans	1	0%
Ornithinibacter aureus	1	0%
unclassified Acanthamoeba	1	0%
unclassified Chloracidobacterales	1	0%
unclassified Amoebophilaceae	1	0%
Nocardioides nitrophenolicus	1	0%
Phaselicystis flava	1	0%

Species	Number of reads	Relative abundance
unclassified Heliobacteriaceae	1	0%
unclassified Ureibacillus	1	0%
Noviherbaspirillum canariense	1	0%
Clostridium aminovalericum	1	0%
Nostoc carneum	1	0%
unclassified Paucibacter	1	0%
Streptomyces lannensis	1	0%
Nocardioides salarius	1	0%
Dechloromonas hortensis	1	0%
Fodinibacter luteus	1	0%
Paenibacillus timonensis	1	0%
unclassified Tenericutes	1	0%
Clostridium sphenoides	1	0%
Ramlibacter tataouinensis	1	0%
Arthrobacter roseus	1	0%
Kroppenstedtia guangzhouensis	1	0%
Marchantia environmental	1	0%
Clostridium septicum	1	0%
Serratia liquefaciens	1	0%
Psychrobacter urativorans	1	0%
Bacillus shackletonii	1	0%
Micromonospora mirobrigensis	1	0%
unclassified Eggerthella	1	0%
Mycobacterium arabiense	1	0%
Corynebacterium sputi	1	0%
unclassified Micavibrio	1	0%
Rhizobium nepotum	1	0%
unclassified Inquilinus	1	0%
Bartonella elizabethae	1	0%
Sporichthya brevicatena	1	0%
Streptomyces lanatus	1	0%
Herminiimonas glaciei	1	0%
Nostocoida aromativora	1	0%
Roseburia faecis	1	0%
Rickettsiella costelytrae	1	0%
unclassified Hahella	1	0%
Bacteroides vulgatus	1	0%
Georgenia soli	1	0%
Coprococcus comes	1	0%
unclassified Anaeroplasmataceae	1	0%
unclassified Methylopila	1	0%
unclassified Thermomonospora	1	0%
unclassified Geoalkalibacter	1	0%
Saccharomonospora xinjiangensis	1	0%
unclassified Sutterella	1	0%
unclassified Brachybacterium	1	0%
Pilimelia anulata	1	0%
unclassified Rhodothermus	1	0%
Actinoplanes garbadinensis	1	0%
Streptomyces hygroscopicus	1	0%

Species	Number of reads	Relative abundance
unclassified Acidaminobacteraceae	1	0%
Arthrobacter creatinolyticus	1	0%
Acinetobacter guillouiae	1	0%
Streptomyces roseofulvus	1	0%
unclassified Friedmanniella	1	0%
unclassified Salinispora	1	0%
Nocardia endophytica	1	0%
Epilithonimonas lactis	1	0%
Blautia luti	1	0%
unclassified Yonghaparkia	1	0%
unclassified Weeksellaceae	1	0%
Streptomyces griseus	1	0%
Bacillus ginsenggisoli	1	0%
Sphingomonas azotifigens	1	0%
Planifilum fimeticola	1	0%
Candidatus Paracaedibacter	1	0%
unclassified Aurantimonadaceae	1	0%
unclassified Pileolariaceae	1	0%
Novosphingobium stygium	1	0%
unclassified Dialister	1	0%
Parabacteroides distasonis	1	0%
Lysobacter gummosus	1	0%
Sphingomonas soli	1	0%
Polaromonas cryoconiti	1	0%
Sphingobium estrogenivorans	1	0%
unclassified Dehalobacter	1	0%
Arthrobacter oryzae	1	0%
unclassified Actinospica	1	0%
Nocardia soli	1	0%
unclassified Haliea	1	0%
unclassified Kitasatospora	1	0%
unclassified Oscillochloridaceae	1	0%
unclassified Proteus	1	0%
Yersinia kristensenii	1	0%
unclassified Planomicrobium	1	0%
Streptomyces aculeolatus	1	0%
Nocardioides agariphilus	1	0%
unclassified Solwaraspora	1	0%
unclassified Euzebyales	1	0%
Anaerosporobacter mobilis	1	0%
Clostridium gasigenes	1	0%
unclassified Lachnobacterium	1	0%
Arthrobacter chlorophenolicus	1	0%
Actinopolymorpha pittospori	1	0%
Mycobacterium pallens	1	0%
Actinokineospora diospyrosa	1	0%
unclassified Aureimonas	1	0%
Rickettsiaceae endosymbiont	1	0%
unclassified Anaerovorax	1	0%
Mucilaginibacter myungsuensis	1	0%

Species	Number of reads	Relative abundance
Tissierella creatinophila	1	0%
Flavobacterium saliperosum	1	0%
unclassified Phascolarctobacterium	1	0%
Actinoplanes regularis	1	0%
Clostridium thermopalmarium	1	0%
Ruminococcus bromii	1	0%
Clostridium thermosuccinogenes	1	0%
Shimazuella kribbensis	1	0%
Pseudonocardia zijingensis	1	0%
unclassified Sutterellaceae	1	0%
Novosphingobium resinovorum	1	0%
Vitis hybrid	1	0%
unclassified Bacillariophyceae	1	0%
Pseudogracilibacillus auburnensis	1	0%
Rhizobium halophytocola	1	0%
Bacillus infantis	1	0%
unclassified Candidatus Kuenenia	1	0%
unclassified Physcomitrella	1	0%
Aeribacillus pallidus	1	0%
unclassified Geobacillus	1	0%
unclassified Filibacter	1	0%
	31158 (192939 total)	
